# Optimization of ATP Synthase c–Rings for Oxygenic Photosynthesis

**DOI:** 10.3389/fpls.2019.01778

**Published:** 2020-01-30

**Authors:** Geoffry A. Davis, David M. Kramer

**Affiliations:** ^1^ Department of Energy Plant Research Laboratory, Michigan State University, East Lansing, MI, United States; ^2^ Department of Biochemistry and Molecular Biology, Michigan State University, East Lansing, MI, United States

**Keywords:** photosynthesis, adenosine triphosphate synthase, proton motive force, singlet oxygen, electron transfer, bioenergetics

## Abstract

The conversion of sunlight into useable cellular energy occurs *via* the proton–coupled electron transfer reactions of photosynthesis. Light is absorbed by photosynthetic pigments and transferred to photochemical reaction centers to initiate electron and proton transfer reactions to store energy in a redox gradient and an electrochemical proton gradient (proton motive force, *pmf*), composed of a concentration gradient (ΔpH) and an electric field (Δ*ψ*), which drives the synthesis of ATP through the thylakoid F_o_F_1_-ATP synthase. Although ATP synthase structure and function are conserved across biological kingdoms, the number of membrane–embedded ion–binding *c* subunits varies between organisms, ranging from 8 to 17, theoretically altering the H^+^/ATP ratio for different ATP synthase complexes, with profound implications for the bioenergetic processes of cellular metabolism. Of the known *c*–ring stoichiometries, photosynthetic *c*–rings are among the largest identified stoichiometries, and it has been proposed that decreasing the c-stoichiometry could increase the energy conversion efficiency of photosynthesis. Indeed, there is strong evidence that the high H^+^/ATP of the chloroplast ATP synthase results in a low ATP/nicotinamide adenine dinucleotide phosphate (NADPH) ratio produced by photosynthetic linear electron flow, requiring secondary processes such as cyclic electron flow to support downstream metabolism. We hypothesize that the larger *c* subunit stoichiometry observed in photosynthetic ATP synthases was selected for because it allows the thylakoid to maintain *pmf* in a range where ATP synthesis is supported, but avoids excess Δ*ψ* and ΔpH, both of which can lead to production of reactive oxygen species and subsequent photodamage. Numerical kinetic simulations of the energetics of chloroplast photosynthetic reactions with altered *c*–ring size predicts the energy storage of *pmf* and its effects on the photochemical reaction centers strongly support this hypothesis, suggesting that, despite the low efficiency and suboptimal ATP/NADPH ratio, a high H^+^/ATP is favored to avoid photodamage. This has important implications for the evolution and regulation of photosynthesis as well as for synthetic biology efforts to alter photosynthetic efficiency by engineering the ATP synthase.

## Introduction

Oxygenic photosynthetic membranes use light to excite electrons on special chlorophyll molecules to store energy in two forms. Redox energy is stored by light-driven extraction of electrons from water to reduce NADP^+^ to nicotinamide adenine dinucleotide phosphate (NADPH). Phosphorylation potential is stored by a chemiosmotic mechanism (Mitchell, 1961; Mitchell, 1966), coupling the light driven electron transfer reactions to the generation of a proton electrochemical gradient (proton motive force, *pmf*), which in turn drives the synthesis of ATP from ADP + P_i_ through an F-type ATP synthase (reviewed in (Boyer, 1997; Junge and Nelson, 2015).

In green algae and higher plant chloroplasts, *pmf* is stored across the thylakoid membrane in both transmembrane electric field (Δ*ψ*) and a proton concentration gradient (ΔpH) (Cruz et al., 2001), differing from mitochondrial respiratory membranes and most plama membranes, across which the *pmf* is primarily composed of Δ*ψ* (Kashket, 1981; Booth, 1985). Both components are thermodynamically interchangeable driving forces for the chloroplast ATP synthase (Hangarter and Good, 1982; Gräber et al., 1984) so that the total driving force for ATP synthesis can be described as:

(1) $$pmf=\;\Delta \psi_{i-o}+\frac{2.3RT}{F}\Delta pH_{o-i}$$

where Δ*ψ*_i-o_ and ΔpH_o-i_ represent the electric field and proton gradient calculated as the difference in concentrations between the inside (lumen) and outside (stroma), *R* is the universal gas constant, and *F* is Faraday’s constant.

During steady-state photosynthesis, *pmf* is generated by light-driven proton translocation and subsequently consumed by H^+^ efflux from the lumen through the ATP synthase, which are regulated in interdependent ways. The formation of *pmf* is also governed by electron transfer rates, which in turn are controlled by “photosynthetic control,” i.e., the slowing of plastoquinol (PQH_2_) oxidation ten–fold from pH 7.5 to 5.5 at the cytochrome *b_6_f* complex as lumen pH decreases below about 6.5 (Nishio and Whitmarsh, 1993; Hope et al., 1994; Takizawa et al., 2007). In effect, the formation of ΔpH and acidification of the lumen is self-controlled.

The consumption of *pmf* is regulated by control of ATP synthase activity (Kanazawa and Kramer, 2002; Cruz et al., 2005a; Takizawa et al., 2008), which at least in green algae and plants responds to decreases in the capacity of the cell to use photosynthetic energy by restricting the efflux of protons, resulting in buildup of *pmf* and subsequent acidification of the lumen, leading to increased photosynthetic control (reviewed in (Strand and Kramer, 2014) and activation of q_E_, the rapidly reversible form of nonphotochemical quenching (NPQ) (Niyogi and Truong, 2013). As will be discussed below, the impact and mechanisms of *pmf* regulation may be different in other photosynthetic lineages, as in cyanobacteria in which some evidence suggests that pH may not regulate NPQ (Kirilovsky and Kerfeld, 2012).

The buildup of thylakoid ΔpH can have additional effects on the photosynthetic machinery. Strong lumen acidification has been shown, *in vitro*, to release Ca^2+^ from the photosystem II (PSII) oxygen evolving complex (OEC) (Krieger and Weis, 1993), as well as slowing the release of protons from the OEC during PSII turnover (Zaharieva et al., 2011). Based on surveys of experimental data on both pH–mediated regulation and damage to photosynthetic proteins, it was proposed that *pmf* is regulated so that the lumen pH remains above about 5.5 except under environmental stresses (Kramer et al., 1999). In this case, maintaining sufficient free energy in ATP (ΔG_ATP_) requires the storage of at least part of *pmf* in the form of Δ*ψ* (Kramer et al., 2004; Cruz et al., 2005b; Zhang et al., 2009; Davis et al., 2016). This requirement has been validated by observations that the Δ*ψ*/ΔpH ratio is sensitive to environmental stresses (Avenson et al., 2004; Zhang et al., 2009; Davis et al., 2016) and is controlled by specific ion transporters (Checchetto et al., 2012; Armbruster et al., 2014; Kunz et al., 2014; Duan et al., 2016; Herdean et al., 2016a; Herdean et al., 2016b; Schneider et al., 2016; Hohner et al., 2019).

While Δ*ψ* can support ATP synthesis in thylakoids with only moderate or no lumen acidification (Hangarter and Good, 1982; Gräber et al., 1984), a large amplitude Δ*ψ* also has important secondary effects, most importantly in decreasing the free energy barrier for charge recombination in photosynthetic reaction centers (Crofts et al., 1971; Vos et al., 1991; Davis et al., 2016), thus enhancing the production of reactive oxygen species (ROS), particularly singlet oxygen (^1^O_2_) by PSII (Davis et al., 2016). Substantial rates of ^1^O_2_ production can be observed even in wild-type plants during rapid fluctuations in actinic light, which generate large, transient amplitudes of Δ*ψ*, which occur more rapidly than feedback regulation of the light reactions (Davis et al., 2017). We surmise that, because the core electron transfer protein complexes of photosynthesis (PSII, cytochrome *b*_6_ *f*, and PSI) are highly conserved across all oxygenic photosynthetic organisms (Hasan and Cramer, 2012; Cardona, 2015), the effects of *pmf* composition on these reactions are likely to represent a common (if not universal) constraint on photosynthetic energy storage, and that evolution will have selected for systems that can adequately balance the storage of *pmf* in Δ*ψ* and ΔpH to balance the needs for efficient ATP synthesis, homeostasis, and the avoidance of excess ROS production.

### Natural Variation in the Stoichiometry of F_0_ *c*-Subunits

**Figure 1:**
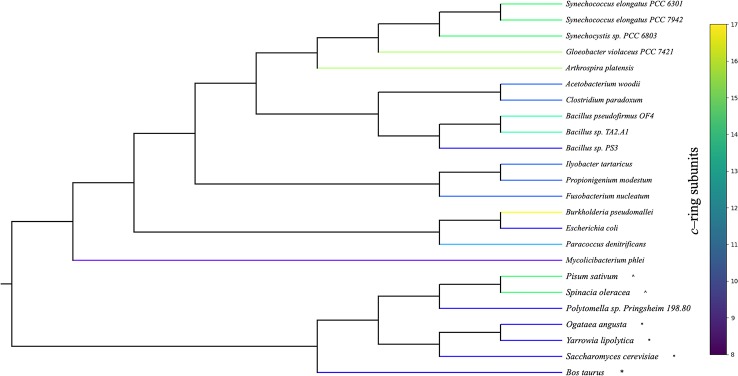
Phylogenetic organization of organisms with known adenosine triphosphate (ATP) synthase *c–ring* stoichiometries. Rooted phylogeny of organisms with experimentally determined *c*–ring stoichiometries retrieved from the National Center for Biotechnology Information (NCBI) taxonomy database. Organism branches colored according to the number of *c* subunits found in the ATP synthase *c*–ring. Cyanobacterial stoichiometries were determined from photosynthetic membranes, and stoichiometries from mitochondria (*) and chloroplasts (^) are indicated for eukaryotic organisms.

ATP synthesis in F_0_F_1_ ATP synthases is thought to involve rotational movement of the membrane embedded F_0_ portion driven by a single ion binding by each *c*–subunit, which is coupled to the catalytic turnover of the F_1_ α_3_β_3_ hexamer to release three ATP molecules per full turnover of the complex (reviewed in Junge and Nelson, 2015). In this mechanism, a full turnover of the F_1_ enzyme is coupled to complete 360° rotation of the *c*–ring, generating three ATP per *c*-subunits, dictating that the number of protons required to generate three ATPs (*n* H^+^/ATP) will be equal to the number of *c*-subunits (Watt et al., 2010). It should be noted that the actual H^+^/ATP stoichiometries have not yet been validated by direct experimentation, and some measurements, based on thermodynamics of *pmf* and ΔG_ATP_, suggest H^+^/ATP of about 4 for both spinach and *Escherichia coli*, independent of the *c* subunit stoichiometry (Turina et al., 2003; Steigmiller et al., 2008). On the other hand, the stoichiometries of bioenergetic processes are notoriously difficult to measure [see e.g., (Steigmiller et al., 2008; Ferguson, 2010)] and while we consider the actual stoichiometry as yet unresolved, the majority of evidence suggests that it should be predominantly controlled by the *c* subunit stoichiometry (Steigmiller et al., 2008). Despite differences in the number of *c* subunits in different organisms, the rotational catalysis mechanism appears to be conserved across biological kingdoms (von Ballmoos et al., 2009; Kuhlbrandt, 2019) and various bioenergetic membranes (Koumandou and Kossida, 2014). While the mechanisms of regulation and the absence/presence of certain peripheral subunits vary between species (Walker, 2013), the core subunits of both F_0_ and F_1_ portions are highly conserved, with the striking exception that the number of *c* subunits that compose the F_0_ ring varies from 8 to 17 (**Figure 1**) (Pogoryelov et al., 2012; Kuhlbrandt, 2019), implying that different species evolved to have widely different H^+^/ATP ratios.

The determinants of *c* subunit stoichiometry are not yet fully understood (Ferguson, 2000; Ferguson, 2010). Each *c* subunit forms two membrane–spanning α–helices embedded within the membrane connected by an F_1_ facing loop (Vonck et al., 2002). Based on structural data from intact *c*–rings, amino acid differences near the N-terminal glycine motif may alter steric and chemical interactions between adjacent α–helices (Vonck et al., 2002), mediating how closely the *c* subunits can pack together (MacKenzie et al., 1997; Watt et al., 2010), with smaller amino acids providing closer packing and smaller *c–*ring stoichiometries (Pogoryelov et al., 2012). While the exact determinants for the *c*–ring stoichiometry is not yet evident, the size does appear to be genetically encoded, resulting from the *c* subunit primary sequence (Muller et al., 2001; Arechaga et al., 2002).

Expressing non–native *c* subunits, either from a different organism or by mutations, results in the assembly of functional chimeric ATP synthases (Laubinger et al., 1990; Suzuki et al., 2007; Liu et al., 2011; Pogoryelov et al., 2012). Supporting the role of *c* subunit primary sequence in determining ring size, replacing only the endogenous *c* subunit gene in *E. coli* ATP synthase with genes from other organisms resulted in *c*–ring stoichiometries matching the organism from which the exogenous *c* subunit was derived, rather than the host *E. coli* stoichiometry (Meier et al., 2005; Matthies et al., 2009). Importantly, the *c*–ring size appears to be determined solely by the sequence of the *c* subunits, and thus remains constant in a species, and does not vary with physiological state (Meyer Zu Tittingdorf et al., 2004; Ballhausen et al., 2009).

### Adenosine Triphosphate Synthesis Energetics Are Impacted by *c*–Ring Stoichiometry

During catalytic turnover of the assembled ATP synthase, the overall rate limitation occurs in the F_1_ portion (Feniouk et al., 2004) and has been attributed to nucleotide binding and exchange (Panke and Rumberg, 1996). However, in thylakoids, the rate of ATP synthase turnover is strongly dependent on the amplitude of *pmf* (Kramer and Crofts, 1989). The ATP synthase is inactive at low *pmf* (Kaplan et al., 1967; Smith et al., 1976), but above the threshold *pmf* required to activate the complex, essentially linear (ohmic) with *pmf* (Smith et al., 1976; Hangarter and Good, 1982; Kramer and Crofts, 1989; Junesch and Graber, 1991). This implies that the rate-limiting step *in vivo* requires *pmf*, and thus changing the *c* stoichiometry should alter not only the thermodynamics (von Ballmoos et al., 2009; Watt et al., 2010; Silverstein, 2014) but also the kineics of ATP synthesis. Indeed, ATP synthases with different *c*–ring stoichiometries have different *pmf* activation thresholds, with larger *c*–ring complexes becoming active (Kaim and Dimroth, 1999) and having higher turnover rates at lower *pmf* amplitudes (Pogoryelov et al., 2012). It has been postulated that the kinetic effects of larger rings are due to increased torque resulting from the smaller step-wise rotation imposed by each proton translocation (von Ballmoos et al., 2008; von Ballmoos et al., 2009), much as shifting to a lower gear on a bicycle allows a rider to mount a steeper hill but at the cost of more energy input per distance traveled.

 It is intriguing that the number of *c* subunits in photosynthetic organisms are all on the high end (13–15 subunits) of the determined *c*–ring stoichiometries (Seelert et al., 2000; Pogoryelov et al., 2005; Pogoryelov et al., 2007), including *Gloeobacter violaceus* PCC 7421, a phylogenetically ancestral, low–light requiring cyanobacterium originally isolated from calcareous rocks (Rippka et al., 1974; Nakamura et al., 2003), which was found to contain a *c*_15_ ring (Pogoryelov et al., 2007).

The energy required to catalyze the synthesis of ATP (ΔG_ATP_) is given by:

(2) $$\Delta G_{ATP}=n*\;\Delta \mu_{H+}$$

where *n* is the H^+^/ATP ratio required to generate each molecule of ATP dictated by the number of *c*–subunits. Assuming the same ΔG_ATP_ between organisms, larger *c*–rings should overcome the energetic barrier for ATP production with a smaller *pmf*, but with a higher overall energy (H^+^) cost. The apparent high H^+^/ATP ratio in chloroplasts (*n*=4.67) decreases the *pmf* required to overcome ΔG_ATP_, allowing photosynthesis to produce ATP at a lower relative *pmf* (Kaim and Dimroth, 1999), reducing the requirement to maintain a large *pmf* (either ΔpH or Δ*ψ*) during steady–state photosynthesis.

However, a higher H^+^/ATP implies that the output of ATP/NADPH for linear electron flow (LEF) will be lower, and in the case of chloroplasts, should result in 2.57 ATP/2 NADPH, below that needed to support the assimilatory reactions of the Calvin-Benson-Bassham (CBB) cycle (Allen, 2003). The resulting energy imbalance requires that chloroplasts activate processes to make up the differences in response to photosynthetic output capacity (Kramer and Evans, 2011). These include cyclic electron flow (reviewed in Strand et al., 2016), the water-water-cycle (Asada, 1999), the malate valve (Scheibe, 2004), as well as balancing the adenylate and electron (either ferredoxin or NADPH) requirements of other metabolic processes (Noctor and Foyer, 2000; Walker et al., 2014; Morales et al., 2018) all of which consume (directly or indirectly) photosynthetic energy. Thus, the large *c* stoichiometries in chloroplasts decrease energy efficiency both at the ATP synthase itself and in imposing a need for additional ATP producing reactions that decrease overall quantum efficiency of photosynthesis.

### The Role of the Adenosine Triphosphate Synthase in Feedback Regulation of Photosynthesis

Whereas mitochondria have been found to store *pmf* primarily in Δ*ψ*, chloroplasts store a fraction of *pmf* as ΔpH; partly as a means of feedback regulation of the light reactions, chloroplasts have evolved mechanisms to alter the partitioning of *pmf* into ΔpH, probably to allow for lumen pH-induced regulation of light capture and electron flow to coordinate with downstream metabolic reactions and avoid over-reduction of PSI cofactors (Kanazawa et al., 2017), while maintaining sufficient Δ*ψ* to avoid over–acidification of the lumen (Kramer et al., 1999; Cruz et al., 2001). This has led some to hypothesize that the large *c*–ring stoichiometry in chloroplasts is required to accommodate a smaller Δ*ψ* (von Ballmoos et al., 2008). Using isolated ATP synthases incorporated into liposomes, the Δ*ψ* required to activate ATP synthesis activity was found to be inversely proportional to the *c*–ring size (Kaim and Dimroth, 1999), so that systems with larger stoichiometries should be able to produce ATP at lower *pmf* values. However, while this observation may explain a benefit of larger *c*–rings during induction, this fails to address why a large steady-state Δ*ψ* is not maintained by chloroplasts. Would the large *pmf* required for ATP synthesis with smaller *c* stoichiometries result in deleterious side reactions in the photosynthetic membrane, and if so, could this contribute to an apparent selection for larger *c*–rings?

### Can *c*–Subunit Stoichiometry Be Tuned to Optimize the Thermodynamic Efficiency of Proton–Coupled Adenosine Triphosphate Synthesis?

Based on a flux model from available *pmf* and ATP substrate parameters, Silverstein (Silverstein, 2014) estimated that, with similar previous experimentally measured *pmf* and ΔG_ATP_ levels, the *E. coli* and bovine mitochondrial ATP synthases (*c*_10_ and *c*_8_, respectively) should convert *pmf* to ΔG_ATP_ with about 25% higher efficiency compared to the chloroplast ATP synthase (*c*_14_), and speculated that because photosynthetic organisms have access to readily available sunlight as an energy source, there may have been less evolutionary selection pressure to maximize the thermodynamic efficiency for ATP synthesis compared to organisms that rely on more scarce energy sources (fixed organic molecules). However, it is well known that photosynthetic organisms have adapted to grow in light-limiting conditions (Judd et al., 1964; Stomp et al., 2007; Scanlan et al., 2009), including low light requiring cyanobacteria which have been shown to also have large *c*-rings (Pogoryelov et al., 2007).

Here, we consider alternative reasons for why photosyntehtic organisms have evolved larger *c*-rings. As discussed elsewhere (Takizawa et al., 2007; Strand and Kramer, 2014), acidifying the thylakoid lumen can lead to pH–mediated downregulation of photosynthesis at least in plants and algae, or damage to photosynthetic components (Kramer et al., 1999), and robust mechanisms for maintaining pH homeostasis have evolved to maintain *pmf* predominantly as Δ*ψ* across other bioenergetic membranes (Kashket, 1981; Booth, 1985; Moore et al., 1985). Our previous work, however, showed that high Δ*ψ* can have deleterious effects on photosynthetic machinery (Davis et al., 2016). We therefore propose that a high H^+^/ATP stoichiometry was selected for because it allows photosynthesis to occur at high *pmf* while maintaining low Δ*ψ* and low ΔpH, thus preventing deleterious side reactions.

## Methods

### Computational Kinetic Simulations of Photosynthetic Light Reactions with Altered *c*–Ring Sizes

To explore the impact of ATP synthase *c–*ring stoichiometry, the photosynthetic light reactions were modelled using a previously published model for the basic photosynthetic light reactions of C3 plants (Davis et al., 2017). Briefly, the model includes ordinary differential equations (ODE) with defined rate constants for electron and proton transfer reactions for the light reactions of photosynthesis, including those that generate and affect the thylakoid *pmf*, as well as the biophysical properties of the thylakoid membrane, and the impacts of *pmf* storage as Δ*ψ* and ΔpH on the rate of electron transfer *via* regulation of *b*_6_ *f* turnover, activation of the q_E_ component of NPQ, and the influence of Δ*ψ* on PSII electron recombination. The code allows simulations over time during different conditions. The underlying code and expanded descriptions for all ODE can be found online at Github (https://github.com/protonzilla/Delta_Psi_Py). As the *c*–ring stoichiometry does not appear to change within an organism (Ballhausen et al., 2009), the simulations treat the H^+^/ATP ratio of the ATP synthase as constant for a given simulation, but can be changed between simulations. To investigate how the *c*–ring architecture impacts the light reactions, only the H^+^/ATP ratio was altered as a constant throughout each simulation. The resulting simulations are available as **Supplementary Data Sheet 1**, as well as an interactive Jupyter notebook available online at Github in which the simulations can be recreated.

## Results and Discussion

### Small *c*–Ring Architecture Limits Proton Motive Force Storage and Composition

**Figure 2:**
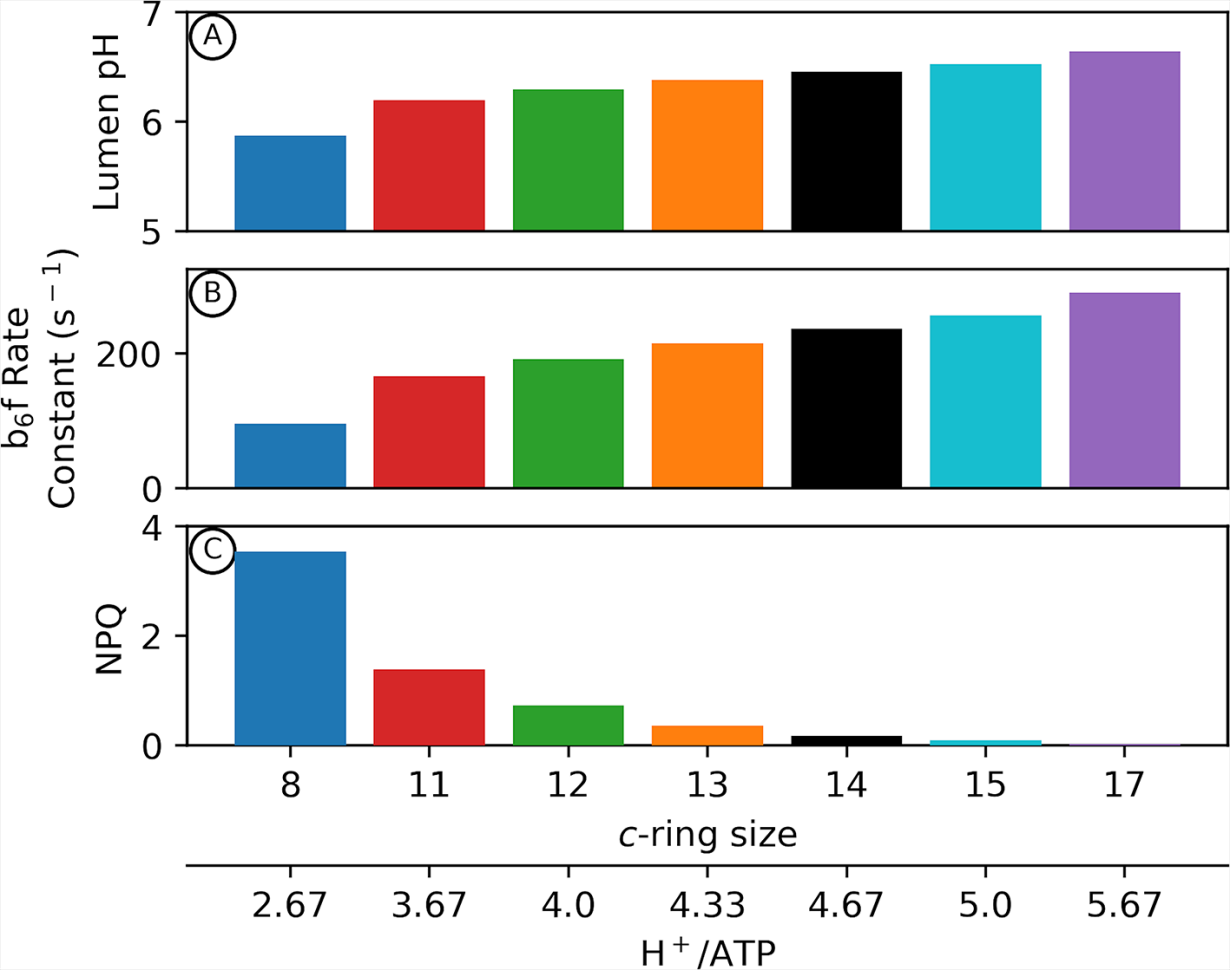
Adenosine triphosphate (ATP) synthase *c*–ring size impacts photosynthetic physiology in the dark. Kinetic modelling of photosynthetic light reactions with altered ATP synthase *c* subunit stoichiometry. Simulated responses of the light reactions were performed as in Davis et al., 2017, with all standard conditions held constant except for the number of ATP synthase c subunits. The *pmf* required to maintain equilibrium with ΔG_ATP_ in the dark is variable depending upon the number of *c* subunits in the ATP synthase *c*–ring (Eq. 2). Changes in lumen pH in the dark due to alterations in *c*–ring size **(A)** can decrease cytochrome *b*_6_ *f* turnover rate **(B)** as well as activate pH–dependent nonphotochemical quenching (NPQ) in higher plants **(C)**.

**Figure 2** shows outputs of our kinetic/thermodynamic simulations under conditions where the ATP synthase is active, but with no or very low net changes in proton flux, so that *pmf* approached equilibrium with ΔG_ATP_ over the course of the simulations. Physiologically this condition should occur at very low light, or when the chloroplast is placed in darkness but the ATP synthase regulatory thiols have not yet become fully oxidized. Thus, these estimates represent the minimum *pmf* needed to sustain ΔG_ATP_.

Assuming a ΔG_ATP_ of 40 kJ/mol (Giersch et al., 1980), a decrease in the number of *c* subunits from 14 (chloroplast) (Seelert et al., 2000) to 8 (*Bos taurus* mitochondria) (Watt et al., 2010) results in an increase in the *pmf* required to maintain ΔG_ATP_ equilibrium in the dark from ~89 to ~155 mV (Eq. 2). If the fraction of *pmf* is equally partitioned between Δ*ψ* and ΔpH, this results in a ΔpH at ΔG_ATP_ of about 0.75 units with a *c*_14_ ring (Eq. 1). Assuming stromal pH of 7.8, the lumen pH should reach about 7, where the violaxanthin de-epoxidase (VDE) and the PsbS protein are inactive, and the *b_6_f* complex is fully active (Takizawa et al., 2007), thus allowing for maximal photosynthetic efficiency at low light (**Figure 2**). By contrast, with a *c*_8_ ATP synthase, ΔpH at ΔG_ATP_ should reach 1.3 units and a lumen pH of 6.5, which is sufficiently acidic to activate VDE and protonate PsbS, thus activating q_E_, while slowing electron flow through the *b_6_f* complex, even in the dark (**Figures 2B, C**). Small *c*-rings have even more severe lumen pH-related effects if *pmf* is stored predominantly in ΔpH, as previously discussed (Kramer et al., 1999; Kramer et al., 2003).

**Figure 3:**
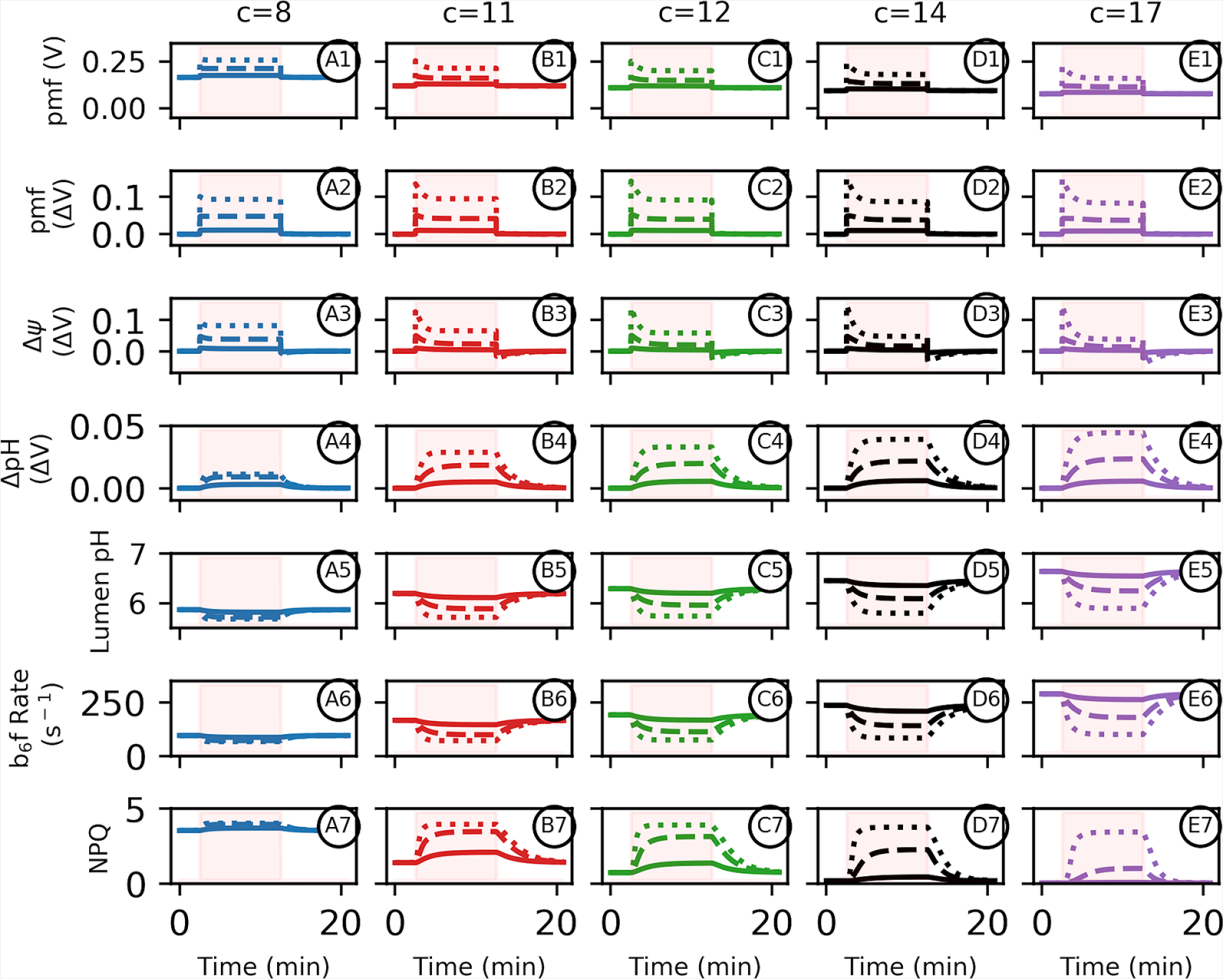
Altered adenosine triphosphate (ATP) synthase *c*-subunit stoichiometry limits proton motive force (*pmf*) composition and pH–mediated regulatory processes during photosynthesis. Simulated responses of the light reactions were performed as in Davis et al., 2017, with all standard conditions held constant except for the number of ATP synthase *c*-subunits. Simulations were performed using 10 min of static light at either 20 (solid lines), 100 (dashed lines), or 1,000 (dotted lines) μmol photons m^−2^s^−1^. Intervals of light excitation are indicated by shaded regions. **(Panels 1–4)** The light–induced *pmf* **(1, 2A–D)** of ATP synthases with *c*–stoichiometries of 8 (blue, **colum**n **A**), 11 (red, **column B**), 12 (green, **column C**), 14 (black, **column D**), or 17 (purple, **column E**) are shown in units of volts, so that a ΔpH of one is equivalent to 0.06 V. The total *pmf* **(panel 2)**, Δ*ψ* **(panel 3)**, and ΔpH (**panel 4**) are shown as light–induced changes relative to the *pmf* dark values indicated as ΔV from dark values, to emphasize light–induced ATP synthase constraints. **(5)** Light–induced changes in lumen pH due to photosynthetic activity. Light intensities and *c*–ring composition as in **(1)**. **(6)** The relative rate constant for plastoquinol oxidation at the cytochrome *b*_6_ *f* complex and **(7)** the extent of nonphotochemical quenching q_E_ component for each *c*–ring size due to the light–induced changes in lumen pH.

Similarly, the Δ*ψ* required just to maintain ΔG_ATP_ equilibrium in the dark increases by ~33 mV when going from *c*_14_ to *c*_8_ if *pmf* was equally partitioned with ΔpH. During photosynthesis, *pmf* is held out of equilibrium from ΔG_ATP_ (see below) so that light-induced *pmf* generation should exacerbate these increases (**Figure 3**). Under this hypothetical smaller *c*_8_ operating structure, photosynthetic *pmf* would either need to be limited to a lower total *pmf* than its current *c*_14_ state, or require a dramatic shift in *pmf* partitioning into Δ*ψ* to avoid near immediate over-acidification of the thylakoid lumen below ~5.5 (ΔpH 2.3 units assuming stromal pH 7.8 in the light) (Kramer et al., 1999), or the evolution of a less pH-sensitive oxygen evolving complex (Krieger and Weis, 1993). A shift in partitioning in favor of Δ*ψ* could occur *via* genetic regulation of counter-ion movement through ion transport expression (Davis et al., 2017), or an increase in the buffering capacity of the lumen, though this might require massive remodelling of thylakoids, or the use of high concentrations of mobile buffering groups such as polyamines (Ioannidis et al., 2012).

While the kinetic/thermodynamic model used to analyse changes in *c* stoichiometries was based upon higher plant light chloroplasts, it should be noted that in cyanobacteria, where photosynthetic and respiratory electron transport share the same membrane and quinone pool (Mullineaux, 2014), the *c* subunit stoichiometry and *pmf* partitioning will impact electron transfer within both processes due to the shared role of *b*_6_ *f* (Vermaas, 2001), though the *pmf*-dependencies of *b_6_f* turnover, OEC stability, or PSII recombination reactions have not been well explored in these species. However, as discussed above, cyanobacterial thylakoid ATP synthase *c*–rings are similar in size to chloroplast *c*–rings rather than bacterial respiratory rings (e.g., *E. coli*), possibly indicating that a large *c*–ring, with its corresponding limitations and advantages, while not necessarily optimized for respiration is less unfavorable to respiratory electron transfer than a small *c*–ring is unfavourable to photosynthetic electron transfer.

We next considered the effects of light activation of electron flow (**Figure 3**). With sufficiently high ATP synthase activity, it should be possible to maintain near equilibrium between *pmf* and ΔG_ATP_ even during photosynthesis. *In vivo,* however, the chloroplast ATP synthase activity is not sufficient to allow equilibration to occur, even under ideal conditions, and further down-regulation of control of ATP synthase activity under adverse conditions results in substantial disequilibrium with ΔG_ATP_ (Buchanan, 1980; Kanazawa and Kramer, 2002). This disequilibrium may have evolved to control lumen pH to activate photosynthetic control and q_E_ (Kanazawa and Kramer, 2002; Avenson et al., 2004) while maintaining ATP homeostasis, or to maintain photosynthetic control by imposing rate limitations at the *b*_6_ *f* complex. It could also represent a fundamental limitation in the kinetic properties of the ATP synthase, though in this case, it is not clear why this limitation could be overcome by over-expressing the complex. In the current simulations, we set ATP synthase activity constant at the highest levels we have observed *in vivo* based on analyses of the decay of the electrochromic shift in the dark (Zaks et al., 2012; Davis et al., 2017).

While the dark, initial *pmf*, which is set be in equilibrium with ΔG_ATP_, increases with smaller *c–*rings (**Figures 2** and **3**), the total light-induced *pmf* (**Figure 3** panel 2) is strongly limited to about the same extents regardless of the *c*–ring stoichiometry due to lumen pH–mediated photosynthetic control (**Figure 3** panel 5) and q_E_. Therefore, the total light–induced *pmf* progressively decreases with smaller *c*–rings (**Figure 3** panels 2–4 ). Thus, the only way to increase total *pmf* storage in small *c*-rings is to store a higher fraction as Δ*ψ* (**Figure 3** panel 3). The light–induced *pmf* challenge is present at both low and high light intensities, with larger *c*–rings being preferential to photosynthetic electron transfer under all intensities. This is clearly seen in the rates of turnover of the *b_6_f* complex (**Figure 3** panel 6). With larger *c*-rings, the lumen pH is above the pKa for PQH_2_ oxidation at low light, so photosynthetic control is low, but at high light, lumen acidification increases ΔpH and the turnover rate of the *b_6_f* complex decreases. In smaller *c*-rings, the lumen pH is initially sufficiently acidic that photosynthetic control is large even at low light, limiting electron flow and further acidification. Thus, *variable* photosynthetic control is lost with the smaller *c*-rings. Note that because PQH_2_ oxidation is the rate-limiting step in linear electron flow, *b*_6_ *f* turnover can be estimated by the re-reduction kinetics of P_700_^+^. Given that ΔpH with smaller *c*-rings is saturated, we expect little effect of changing downstream reactions on photosynthetic control.

**Figure 4:**
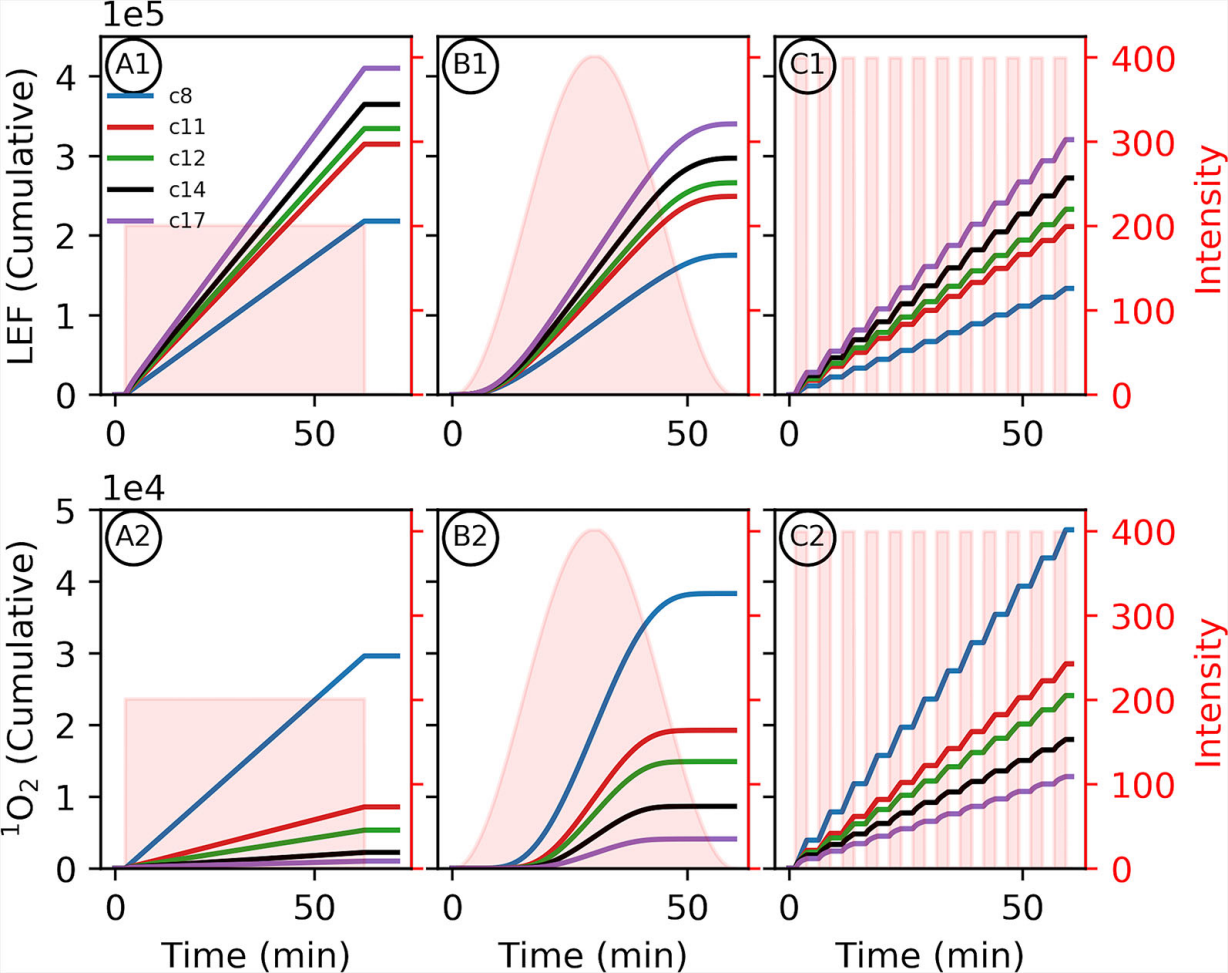
Altered proton motive force (*pmf*) composition due to *c*-subunit stoichiometry limits photosynthetic productivity. Simulated responses of the light reactions were performed as in **Figure 3**. Variability in environment was simulated with 1-h light profiles of static light **(A1, 2)**, sinusoidal light **(B1, 2)**, or square wave fluctuating light **(C1, 2)** to provide the same total illumination during the simulation. **(1)** The total outputs for linear electron flow (LEF) over the course of the light simulations and **(2)** ^1^O_2_ were integrated over the light treatment to give the cumulative totals. Shaded regions indicate the light profiles for each simulation.

However, higher Δ*ψ* increases the rates of recombination reactions in PSII and ^1^O_2_ production (**Figure 4**) (Davis et al., 2016). The PSII recombination rate depends on the concentration of charge–separated states capable of recombining as well as the energetics of electron sharing between redox intermediates, with the rate changing exponentially with Δ*ψ*. For the charge-separated state(s) forming P^+^Q_A_^−^, where P^+^ is the primary electron donor and Q_A_ the non-mobile PSII quinone, these changes correspond to:

(3) $$v_{recombination}=\left[S_{2}Q_{A}{\;}^{-}+\;S_{3}Q_{A}{\;}^{-}\right]*\;k_{r}*10^{\frac{\Delta E_{stab}-f*\Delta \psi }{0.06}}\;\;\;\;\;\;\;\;\;\;\;\;\;\;\;\;\;$$

where [S_2_Q_A_^−^ + S_3_Q_A_^−^] represents the fraction of PSII containing donor and acceptor side states capable of recombining from Q_A_^−^, *k*_r_ is the intrinsic rate of recombination from S_2_/S_3_Q_A_^−^ with no Δ*ψ*, ΔE*_stab_* is the stabilization free energy of the charge-separated state S_2_/S_3_Q_A_^−^ expressed in eV, and *f* the distance between the charge-separated states normal to the membrane surface (Davis et al., 2016). As Δ*ψ* increases, the ΔE*_stab_* of charge separated states decreases (de Grooth and van Gorkom, 1981; Vos et al., 1991), leading to an increase in the rate of recombination. Therefore, even with relatively small changes in the amount of energy stored as Δ*ψ*, the velocity of recombination will increase dramatically. This likely limits the amount of energy that can be stored safely across photosynthetic membranes as Δ*ψ*, as electron recombination through back–reactions can lead to generation of ROS (Rutherford et al., 2012; Davis et al., 2016).

As seen in **Figure 3**, relative to larger stoichiometries, the increased Δ*ψ* in smaller *c*–rings (**Figure 3** panel 3) is expected to increase the probability of PSII recombination reactions and ^1^O_2_ generation. This small *c*–ring pitfall is exacerbated by the constitutive downregulation of electron transfer due to the lower lumen pH, limiting productive LEF and resulting in a more reduced quinone pool, which provides substrate for PSII recombination (**Supplementary Figures 1**–**8**). Dynamic light intensities (**Figure 4**), as would be expected in many natural environments, can induce large spikes in Δ*ψ* (Cruz et al., 2001; Davis et al., 2016) that can exacerbate PSII recombination-induced ^1^O_2_ production, and our simulations suggest that this effect is worsened as *c* subunit stoichiometry decreases. Simulations of one hour of illumination at light intensities of various dynamics but equal total photon flux, recapitulate the increased production of ^1^O_2_ with increasing intensity of light fluctuations (**Figure 4** panel 2). Smaller *c*–rings, as with static light, produce even more ^1^O_2_ than the photosynthetic *c*_14_ with progressively more with more realistic, fluctuating light dynamics (**Figure 4C**). Coupled with the potential cellular damage from ^1^O_2_, small *c*–rings, even with higher light intensities than static light, result in progressively less LEF over the course of illumination in dynamic conditions due to premature downregulation of quinol oxidation relative to large stoichiometries (**Figure 4** panel 1).

It therefore appears that a large *c*–ring stoichiometry, although arguably energetically inefficient in isolation (von Ballmoos et al., 2008; Silverstein, 2014), may in fact be far more physiologically efficient for photoautotrophic maintenance and organismal survival than a smaller stoichiometry. Additionally, the decrease in the chloroplast ATP synthase *pmf* activation threshold (Kaim and Dimroth, 1999) may have led to the additional advantage of photosynthetic ATP generation under even low light and low *pmf* conditions (Kramer and Crofts, 1989).

### Are Large *c*-Rings an Evolutionary Adaptation to Utilize Large ΔpH and Synthesize Adenosine Triphosphate When Proton Motive Force Is Small?

Based on the arguments above for phototrophs, one might also expect to find higher *c*-stoichiometries in other organisms where *pmf* is constrained by similar needs to maintain physiologically permissive conditions. Interestingly, of the ATP synthase *c*-rings analyzed to date, the other group outside of oxygenic photoautotrophs that utilize larger (*c*_13_) rings are alkaliphilic bacteria (Meier et al., 2007; Preiss et al., 2010). Due to their high pH growth conditions, these organisms are subjected to an “inverted” ΔpH, acidic inside the cell relative to outside, and must generate a high Δ*ψ* in order to allow H^+^ driven ATP synthesis (Sturr et al., 1994; Olsson et al., 2003; Hicks et al., 2010). However, the inverted ΔpH works against total *pmf*, so that the operating *pmf* is lower than that in neutrophilic bacteria, possibly because there is a thermodynamic or structural limit to the amplitude of Δ*ψ* needed to counterbalance the negative ΔpH. Thus, a larger *c*-ring may be necessary to maintain sufficient *pmf* to overcome ΔG_ATP_, which could explain the growth defects found in the alkaliphilic *Bacillus pseudofirmus* OF4 with engineered smaller *c*-rings (Liu et al., 2011; Preiss et al., 2013).

More examples of *c*-ring architecture combined with *pmf* [or sodium motive force (*smf*)] measurements could resolve why some organisms have evolved to use larger, theoretically less efficient H^+^/ATP ratios. The monophyly of oxygenic photosynthesis in a single group of bacteria (cyanobacteria) may have initiated *c*–ring constraints in chloroplasts. The *pmf* composition and dynamics in cyanobacteria has not yet been well studied, whereas an electrochromic shift of carotenoid pigments in the thylakoid membranes of eukaryotes (Bailleul et al., 2010), has allowed focused studies primarily in the green lineage (Bailleul et al., 2015). The recent report of a useable electrochromic shift in a cyanobacterium should make such studies possible (Viola et al., 2019).

While we emphasize the larger effects of changing *c* subunit stoichiometry by large amounts, but as expected, the effects of smaller changes on *pmf* are incremental, and thus we expect there to be subtler trade-offs. However, even small tradeoffs are expected to be important over evolutionary time scales. One additional, and interesting, tradeoff is that a *c*_12_ ring would be expected to balance the ATP/NADPH production by LEF with consumption by the CBB cycle, perhaps obviating the need for cyclic electron flow under some conditions. However, the overall ATP/NADPH demand is dynamic and should depend on which metabolic sinks are engaged (Heber, 1974; Krause and Heber, 1976; Edwards and Walker, 1983; Fernyhough et al., 1983; Kobayashi et al., 1995; Kramer and Evans, 2011), and thus energy balancing mechanisms should still be required. Making the rings are smaller than 12 subunits would introduce a different problem: an excess of ATP/NADPH production relative to consumption by the CBB cycle; interestingly, there are no established mechanisms to ameliorate this kind of imbalance (Kanazawa et al., 2020). This issue is more apparent (and acute) in cyanobacteria, which use dissipative oxygen reduction pathways to protect reactions centers from photodamage, but as a consequence introduce ATP/NADPH imbalances. Thus, a greater understanding of the *pmf* and ATP synthase architecture in cyanobacteria will enhance the understanding of bioenergetic interactions between photosynthetic electron transfer and ATP production. This understanding is crucial for any future synthetic biology approaches to alter photosynthetic ATP production either directly *via* the ATP synthase (Cardona et al., 2018), or indirectly *via* the H^+^/e^−^ ratio of ATP/NADPH. These limitations and trade-offs, we predict, will likely hinder any gains in photosynthetic efficiency afforded by engineering smaller *c*–rings unless radical changes to the rest of photosynthesis are also made.

## Data Availability Statement

The datasets generated for this study can be found on github (https://github.com/protonzilla/Delta_Psi_Py) as a detailed Jupyter (www.jupyter.org) notebook.

## Author Contributions

GD and DK contributed to the design of simulations and interpretation of results. GD and DK contributed to the drafting and revising of the article.

## Funding

Funding for GD and DK was supported by the US Department of Energy (DOE), Office of Basic Science, Basic Energy Sciences (BES) under award number DE-FG02-91ER20021.

## Conflict of Interest

The authors declare that the research was conducted in the absence of any commercial or financial relationships that could be construed as a potential conflict of interest.

## Supplementary Material

Supplementary Figure 1 Comparison of pmf composition and pH–mediated regulatory processes during photosynthesis in naturally occurring photosynthetic c-rings and c12 ring to balance ATP/NADPH with carbon assimilation. Simulated responses of the light reactions were performed as in Davis et al., 2017, with all standard conditions held constant except for the number of ATP synthase *c*-subunits. Simulations were performed using 10 minutes of static light at either 20 (solid lines), 100 (dashed lines), or 1000 (dotted lines) μmol photons m^-2^s^-1^ as in Figure 1. Intervals of light excitation are indicated by shaded regions. (Panels 1-4) The light–induced *pmf* (1, 2A-D) of ATP synthases with *c*–stoichiometries of 12 (green, column A), 13 (orange, column B), 14 (black, column C), or 15 (cyan, column D) are shown in units of volts, so that a ΔpH of one is equivalent to 0.06 V. The total *pmf* (panel 2), Δ*ψ* (panel 3), and ΔpH (panel 4) are shown as light–induced changes relative to the *pmf* dark values indicated as ΔV from dark values, to emphasize light–induced ATP synthase constraints. (5) Light–induced changes in lumen pH due to photosynthetic activity. Light intensities and *c*–ring composition as in (1). (6) The relative rate constant for plastoquinol oxidation at the cytochrome *b_6_f* complex and (7) the extent of nonphotochemical quenching q_E_ component for each *c*–ring size due to the light–induced changes in lumen pH.

Supplementary Figure 2 Altered ATP synthase *c_8_* stoichiometry impacts *pmf* composition and pH–mediated regulatory processes during photosynthesis under increasingly dynamic light environments. Simulated responses of the light reactions were performed as in Davis et al., 2017, with all standard conditions held constant except for the number of ATP synthase *c*-subunits. Simulations were performed using 1-hour of either static light (A), sinusoidal light (B), or square wave fluctuating light (C) with equal total photon flux over the total duration of each light treatment. Intervals of light excitation are indicated by shaded regions. (Panels 1-4) The light–induced *pmf* of ATP synthases with *c*–stoichiometries of 8 (blue) or 14 (black) are shown in units of volts, so that a ΔpH of one is equivalent to 0.06 V. The total *pmf* (panel 2), Δ*ψ* (panel 3), and ΔpH (panel 4) are shown as light–induced changes relative to the *pmf* dark values indicated as ΔV from dark values, to emphasize light–induced ATP synthase constraints. (5) Light–induced changes in lumen pH due to photosynthetic activity. Light intensities and *c*–ring composition as in (1). (6) The relative rate constant for plastoquinol oxidation at the cytochrome *b_6_f* complex and (7) the extent of nonphotochemical quenching q_E_ component for each *c*–ring size due to the light–induced changes in lumen pH.

Supplementary Figure 3 Altered ATP synthase *c*_11_ stoichiometry impacts *pmf* composition and pH–mediated regulatory processes during photosynthesis under increasingly dynamic light environments. Simulated responses of the light reactions were performed as in Davis et al., 2017, with all standard conditions held constant except for the number of ATP synthase *c*-subunits. Simulations were performed using 1-hour of either static light (A), sinusoidal light (B), or square wave fluctuating light (C) with equal total photon flux over the total duration of each light treatment. Intervals of light excitation are indicated by shaded regions. (Panels 1-4) The light–induced *pmf* of ATP synthases with c–stoichiometries of 11 (red) or 14 (black) are shown in units of volts, so that a ΔpH of one is equivalent to 0.06 V. The total *pmf* (panel 2), Δ*ψ* (panel 3), and ΔpH (panel 4) are shown as light–induced changes relative to the *pmf* dark values indicated as ΔV from dark values, to emphasize light–induced ATP synthase constraints. (5) Light–induced changes in lumen pH due to photosynthetic activity. Light intensities and *c*–ring composition as in (1). (6) The relative rate constant for plastoquinol oxidation at the cytochrome b6f complex and (7) the extent of nonphotochemical quenching q_E_ component for each *c*–ring size due to the light–induced changes in lumen pH.

Supplementary Figure 4 Altered ATP synthase *c*_12_ stoichiometry impacts *pmf* composition and pH–mediated regulatory processes during photosynthesis under increasingly dynamic light environments. Simulated responses of the light reactions were performed as in Davis et al., 2017, with all standard conditions held constant except for the number of ATP synthase *c*-subunits. Simulations were performed using 1-hour of either static light (A), sinusoidal light (B), or square wave fluctuating light (C) with equal total photon flux over the total duration of each light treatment. Intervals of light excitation are indicated by shaded regions. (Panels 1-4) The light–induced *pmf* of ATP synthases with *c*–stoichiometries of 12 (green) or 14 (black) are shown in units of volts, so that a ΔpH of one is equivalent to 0.06 V. The total *pmf* (panel 2), Δ*ψ* (panel 3), and ΔpH (panel 4) are shown as light–induced changes relative to the *pmf* dark values indicated as ΔV from dark values, to emphasize light–induced ATP synthase constraints. (5) Light–induced changes in lumen pH due to photosynthetic activity. Light intensities and *c*–ring composition as in (1). (6) The relative rate constant for plastoquinol oxidation at the cytochrome b6f complex and (7) the extent of nonphotochemical quenching q_E_ component for each *c*–ring size due to the light–induced changes in lumen pH.

Supplementary Figure 5 Altered ATP synthase *c*_13_ stoichiometry impacts *pmf* composition and pH–mediated regulatory processes during photosynthesis under increasingly dynamic light environments. Simulated responses of the light reactions were performed as in Davis et al., 2017, with all standard conditions held constant except for the number of ATP synthase *c*-subunits. Simulations were performed using 1-hour of either static light (A), sinusoidal light (B), or square wave fluctuating light (C) with equal total photon flux over the total duration of each light treatment. Intervals of light excitation are indicated by shaded regions. (Panels 1-4) The light–induced pmf of ATP synthases with *c*–stoichiometries of 13 (orange) or 14 (black) are shown in units of volts, so that a ΔpH of one is equivalent to 0.06 V. The total *pmf* (panel 2), Δ*ψ* (panel 3), and ΔpH (panel 4) are shown as light–induced changes relative to the *pmf* dark values indicated as ΔV from dark values, to emphasize light–induced ATP synthase constraints. (5) Light–induced changes in lumen pH due to photosynthetic activity. Light intensities and *c*–ring composition as in (1). (6) The relative rate constant for plastoquinol oxidation at the cytochrome b6f complex and (7) the extent of nonphotochemical quenching q_E_ component for each *c*–ring size due to the light–induced changes in lumen pH.

Supplementary Figure 6 Altered ATP synthase *c*_15_ stoichiometry impacts *pmf* composition and pH–mediated regulatory processes during photosynthesis under increasingly dynamic light environments. Simulated responses of the light reactions were performed as in Davis et al., 2017, with all standard conditions held constant except for the number of ATP synthase *c*-subunits. Simulations were performed using 1-hour of either static light (A), sinusoidal light (B), or square wave fluctuating light (C) with equal total photon flux over the total duration of each light treatment. Intervals of light excitation are indicated by shaded regions. (Panels 1-4) The light–induced pmf of ATP synthases with c–stoichiometries of 15 (cyan) or 14 (black) are shown in units of volts, so that a ΔpH of one is equivalent to 0.06 V. The total *pmf* (panel 2), Δ*ψ* (panel 3), and ΔpH (panel 4) are shown as light–induced changes relative to the *pmf* dark values indicated as ΔV from dark values, to emphasize light–induced ATP synthase constraints. (5) Light–induced changes in lumen pH due to photosynthetic activity. Light intensities and c–ring composition as in (1). (6) The relative rate constant for plastoquinol oxidation at the cytochrome *b_6_f* complex and (7) the extent of nonphotochemical quenching q_E_ component for each *c*–ring size due to the light–induced changes in lumen pH.

Supplementary Figure 7 Altered ATP synthase *c*_17_ stoichiometry impacts *pmf* composition and pH–mediated regulatory processes during photosynthesis under increasingly dynamic light environments. Simulated responses of the light reactions were performed as in Davis et al., 2017, with all standard conditions held constant except for the number of ATP synthase *c*-subunits. Simulations were performed using 1-hour of either static light (A), sinusoidal light (B), or square wave fluctuating light (C) with equal total photon flux over the total duration of each light treatment. Intervals of light excitation are indicated by shaded regions. (Panels 1-4) The light–induced *pmf* of ATP synthases with *c*–stoichiometries of 17 (purple) or 14 (black) are shown in units of volts, so that a ΔpH of one is equivalent to 0.06 V. The total pmf (panel 2), Δ*ψ* (panel 3), and ΔpH (panel 4) are shown as light–induced changes relative to the *pmf* dark values indicated as ΔV from dark values, to emphasize light–induced ATP synthase constraints. (5) Light–induced changes in lumen pH due to photosynthetic activity. Light intensities and *c*–ring composition as in (1). (6) The relative rate constant for plastoquinol oxidation at the cytochrome *b_6_f* complex and (7) the extent of nonphotochemical quenching q_E_ component for each *c*–ring size due to the light–induced changes in lumen pH.

Supplementary Figure 8 Altered *pmf* composition due to *c*-subunit stoichiometry limits photosynthetic productivity. Simulated responses of the light reactions were performed as in figures 3 and 4. Variability in environment was simulated with 1-hour light profiles of static light (A1, 2), sinusoidal light (B1, 2), or square wave fluctuating light (C1, 2) to provide the same total illumination during the simulation. (1) The total outputs for linear electron flow (LEF) over the course of the light simulations and (2) 1O2 were integrated over the light treatment to give the cumulative totals. Shaded regions indicate the light profiles for each simulation.
