# Supplementary figures and images for: Optimization of ATP Synthase c–Rings for Oxygenic Photosynthesis
Source: Front Plant Sci. 2020 Jan 30;10:1778. doi: 10.3389/fpls.2019.01778 (PMC7003800; doi:10.3389/fpls.2019.01778)

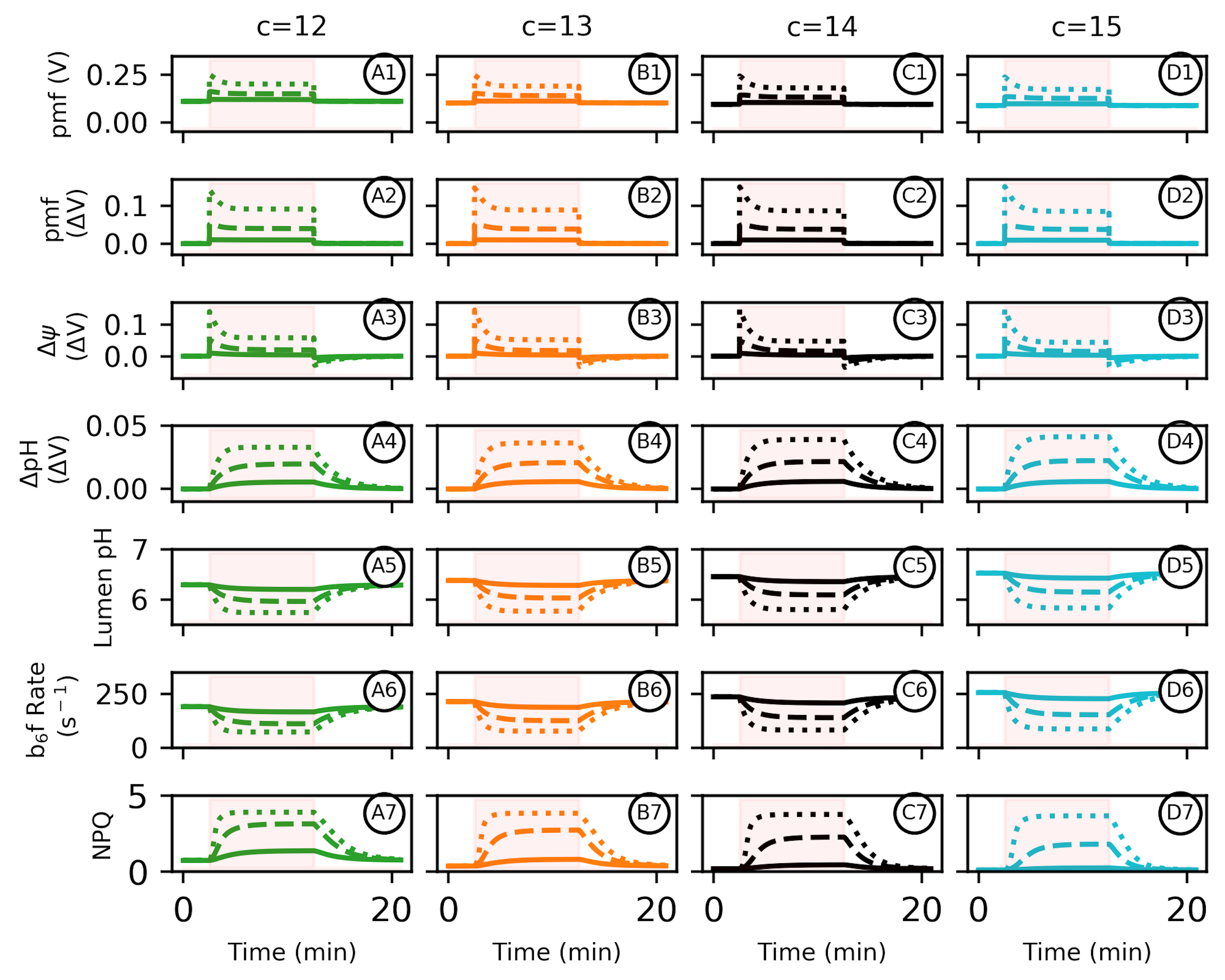

Supplement: Supplementary Figure 1 — Comparison of pmf composition and pH–mediated regulatory processes during photosynthesis in naturally occurring photosynthetic c-rings and c12 ring to balance ATP/NADPH with carbon assimilation. Simulated responses of the light reactions were performed as in Davis et al., 2017, with all standard conditions held constant except for the number of ATP synthase c-subunits. Simulations were performed using 10 minutes of static light at either 20 (solid lines), 100 (dashed lines), or 1000 (dotted lines) μmol photons m-2s-1 as in Figure 1. Intervals of light excitation are indicated by shaded regions. (Panels 1-4) The light–induced pmf (1, 2A-D) of ATP synthases with c–stoichiometries of 12 (green, column A), 13 (orange, column B), 14 (black, column C), or 15 (cyan, column D) are shown in units of volts, so that a ΔpH of one is equivalent to 0.06 V. The total pmf (panel 2), Δψ (panel 3), and ΔpH (panel 4) are shown as light–induced changes relative to the pmf dark values indicated as ΔV from dark values, to emphasize light–induced ATP synthase constraints. (5) Light–induced changes in lumen pH due to photosynthetic activity. Light intensities and c–ring composition as in (1). (6) The relative rate constant for plastoquinol oxidation at the cytochrome b6f complex and (7) the extent of nonphotochemical quenching qE component for each c–ring size due to the light–induced changes in lumen pH. [file Image_1.tiff]

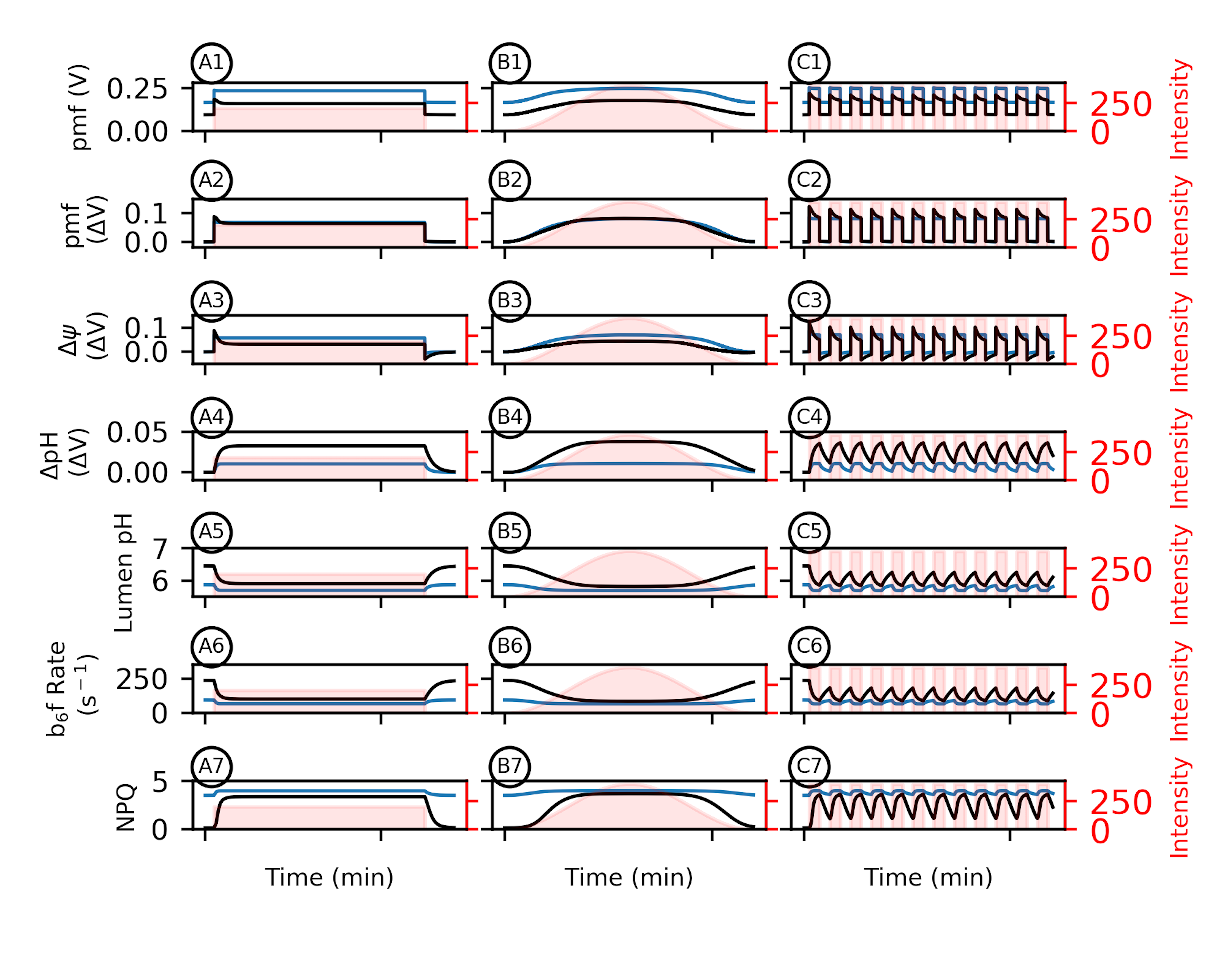

Supplement: Supplementary Figure 2 — Altered ATP synthase c8 stoichiometry impacts pmf composition and pH–mediated regulatory processes during photosynthesis under increasingly dynamic light environments. Simulated responses of the light reactions were performed as in Davis et al., 2017, with all standard conditions held constant except for the number of ATP synthase c-subunits. Simulations were performed using 1-hour of either static light (A), sinusoidal light (B), or square wave fluctuating light (C) with equal total photon flux over the total duration of each light treatment. Intervals of light excitation are indicated by shaded regions. (Panels 1-4) The light–induced pmf of ATP synthases with c–stoichiometries of 8 (blue) or 14 (black) are shown in units of volts, so that a ΔpH of one is equivalent to 0.06 V. The total pmf (panel 2), Δψ (panel 3), and ΔpH (panel 4) are shown as light–induced changes relative to the pmf dark values indicated as ΔV from dark values, to emphasize light–induced ATP synthase constraints. (5) Light–induced changes in lumen pH due to photosynthetic activity. Light intensities and c–ring composition as in (1). (6) The relative rate constant for plastoquinol oxidation at the cytochrome b6f complex and (7) the extent of nonphotochemical quenching qE component for each c–ring size due to the light–induced changes in lumen pH. [file Image_2.tiff]

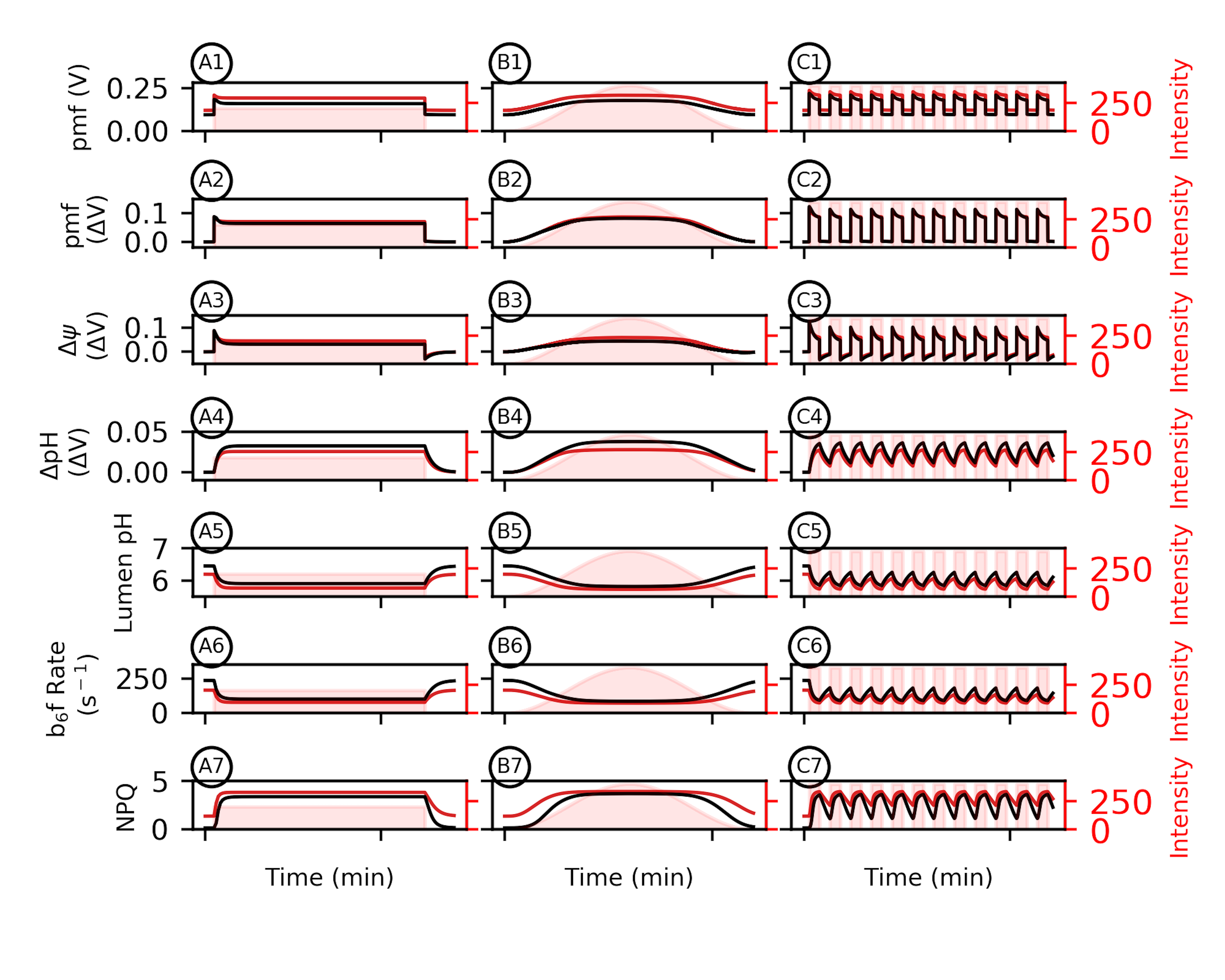

Supplement: Supplementary Figure 3 — Altered ATP synthase c 11 stoichiometry impacts pmf composition and pH–mediated regulatory processes during photosynthesis under increasingly dynamic light environments. Simulated responses of the light reactions were performed as in Davis et al., 2017, with all standard conditions held constant except for the number of ATP synthase c-subunits. Simulations were performed using 1-hour of either static light (A), sinusoidal light (B), or square wave fluctuating light (C) with equal total photon flux over the total duration of each light treatment. Intervals of light excitation are indicated by shaded regions. (Panels 1-4) The light–induced pmf of ATP synthases with c–stoichiometries of 11 (red) or 14 (black) are shown in units of volts, so that a ΔpH of one is equivalent to 0.06 V. The total pmf (panel 2), Δψ (panel 3), and ΔpH (panel 4) are shown as light–induced changes relative to the pmf dark values indicated as ΔV from dark values, to emphasize light–induced ATP synthase constraints. (5) Light–induced changes in lumen pH due to photosynthetic activity. Light intensities and c–ring composition as in (1). (6) The relative rate constant for plastoquinol oxidation at the cytochrome b6f complex and (7) the extent of nonphotochemical quenching qE component for each c–ring size due to the light–induced changes in lumen pH. [file Image_3.tiff]

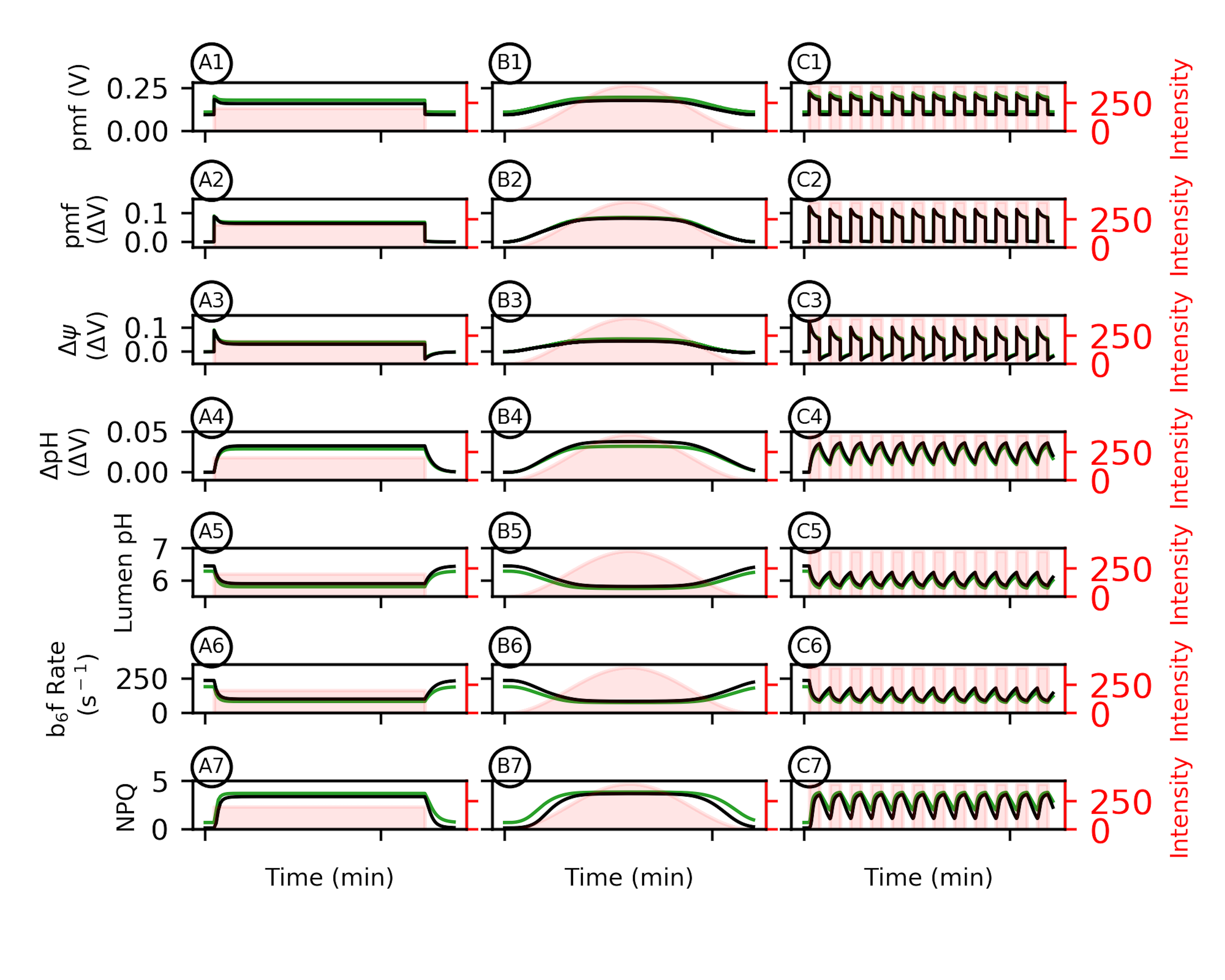

Supplement: Supplementary Figure 4 — Altered ATP synthase c 12 stoichiometry impacts pmf composition and pH–mediated regulatory processes during photosynthesis under increasingly dynamic light environments. Simulated responses of the light reactions were performed as in Davis et al., 2017, with all standard conditions held constant except for the number of ATP synthase c-subunits. Simulations were performed using 1-hour of either static light (A), sinusoidal light (B), or square wave fluctuating light (C) with equal total photon flux over the total duration of each light treatment. Intervals of light excitation are indicated by shaded regions. (Panels 1-4) The light–induced pmf of ATP synthases with c–stoichiometries of 12 (green) or 14 (black) are shown in units of volts, so that a ΔpH of one is equivalent to 0.06 V. The total pmf (panel 2), Δψ (panel 3), and ΔpH (panel 4) are shown as light–induced changes relative to the pmf dark values indicated as ΔV from dark values, to emphasize light–induced ATP synthase constraints. (5) Light–induced changes in lumen pH due to photosynthetic activity. Light intensities and c–ring composition as in (1). (6) The relative rate constant for plastoquinol oxidation at the cytochrome b6f complex and (7) the extent of nonphotochemical quenching qE component for each c–ring size due to the light–induced changes in lumen pH. [file Image_4.tiff]

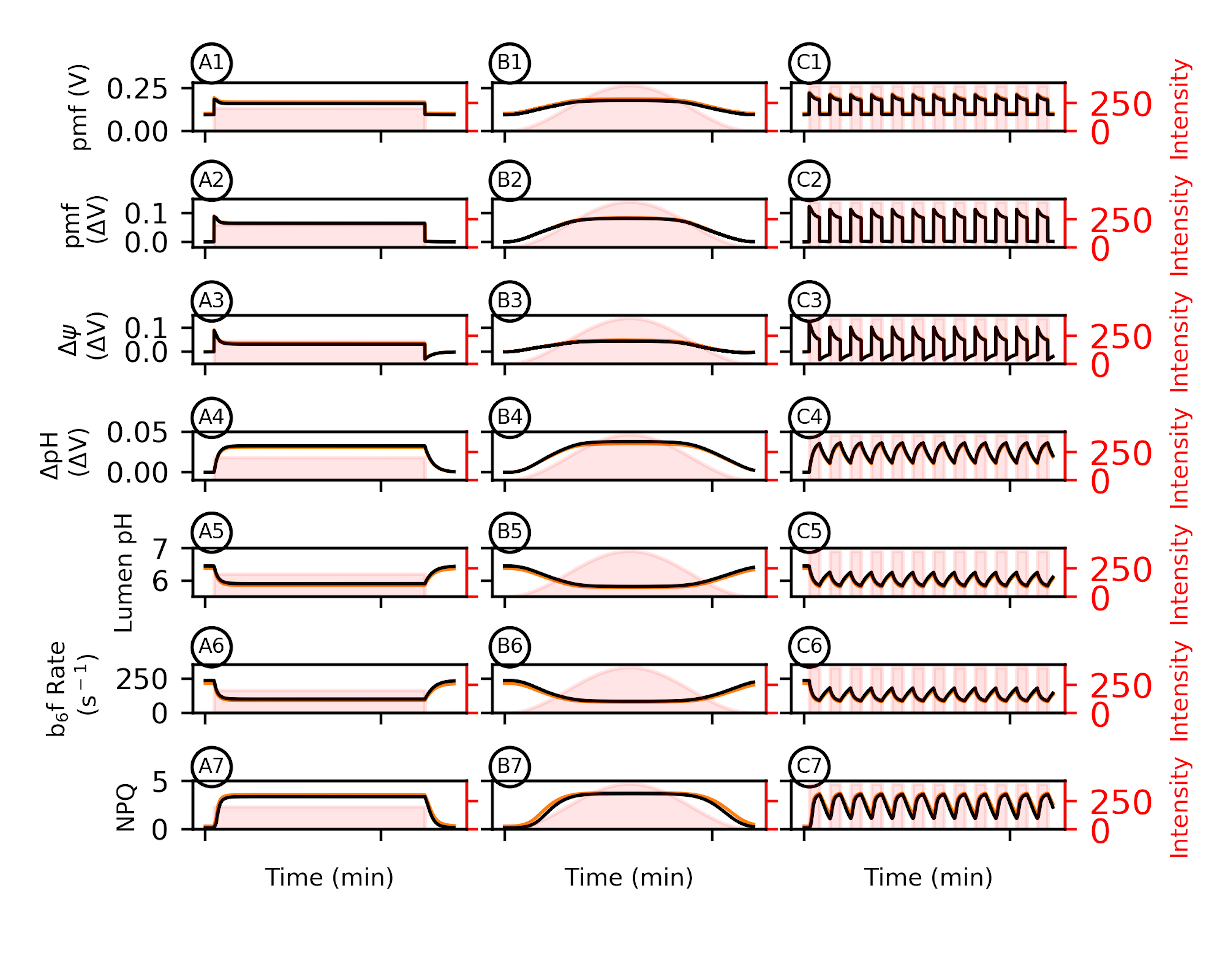

Supplement: Supplementary Figure 5 — Altered ATP synthase c 13 stoichiometry impacts pmf composition and pH–mediated regulatory processes during photosynthesis under increasingly dynamic light environments. Simulated responses of the light reactions were performed as in Davis et al., 2017, with all standard conditions held constant except for the number of ATP synthase c-subunits. Simulations were performed using 1-hour of either static light (A), sinusoidal light (B), or square wave fluctuating light (C) with equal total photon flux over the total duration of each light treatment. Intervals of light excitation are indicated by shaded regions. (Panels 1-4) The light–induced pmf of ATP synthases with c–stoichiometries of 13 (orange) or 14 (black) are shown in units of volts, so that a ΔpH of one is equivalent to 0.06 V. The total pmf (panel 2), Δψ (panel 3), and ΔpH (panel 4) are shown as light–induced changes relative to the pmf dark values indicated as ΔV from dark values, to emphasize light–induced ATP synthase constraints. (5) Light–induced changes in lumen pH due to photosynthetic activity. Light intensities and c–ring composition as in (1). (6) The relative rate constant for plastoquinol oxidation at the cytochrome b6f complex and (7) the extent of nonphotochemical quenching qE component for each c–ring size due to the light–induced changes in lumen pH. [file Image_5.tiff]

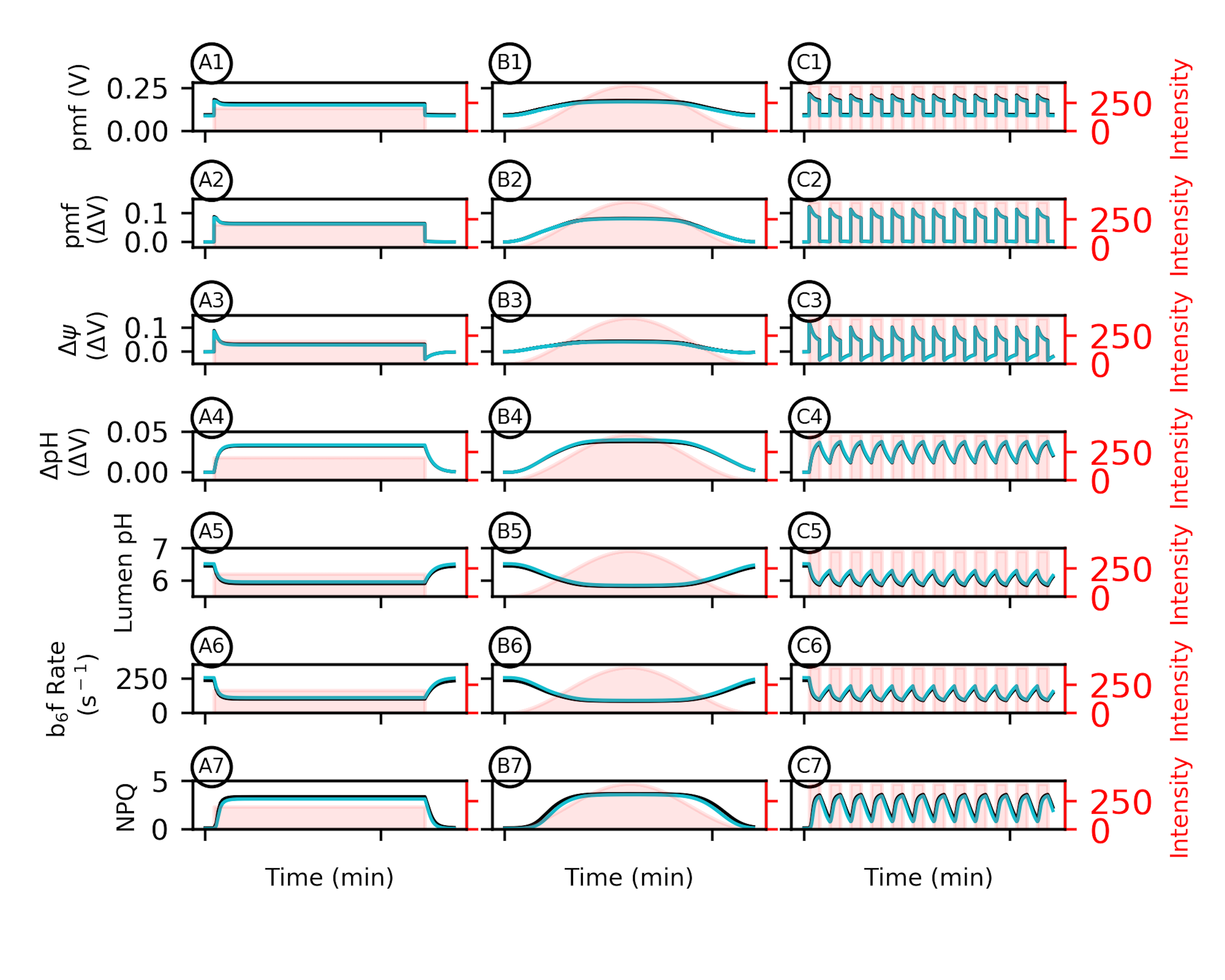

Supplement: Supplementary Figure 6 — Altered ATP synthase c 15 stoichiometry impacts pmf composition and pH–mediated regulatory processes during photosynthesis under increasingly dynamic light environments. Simulated responses of the light reactions were performed as in Davis et al., 2017, with all standard conditions held constant except for the number of ATP synthase c-subunits. Simulations were performed using 1-hour of either static light (A), sinusoidal light (B), or square wave fluctuating light (C) with equal total photon flux over the total duration of each light treatment. Intervals of light excitation are indicated by shaded regions. (Panels 1-4) The light–induced pmf of ATP synthases with c–stoichiometries of 15 (cyan) or 14 (black) are shown in units of volts, so that a ΔpH of one is equivalent to 0.06 V. The total pmf (panel 2), Δψ (panel 3), and ΔpH (panel 4) are shown as light–induced changes relative to the pmf dark values indicated as ΔV from dark values, to emphasize light–induced ATP synthase constraints. (5) Light–induced changes in lumen pH due to photosynthetic activity. Light intensities and c–ring composition as in (1). (6) The relative rate constant for plastoquinol oxidation at the cytochrome b6f complex and (7) the extent of nonphotochemical quenching qE component for each c–ring size due to the light–induced changes in lumen pH. [file Image_6.tiff]

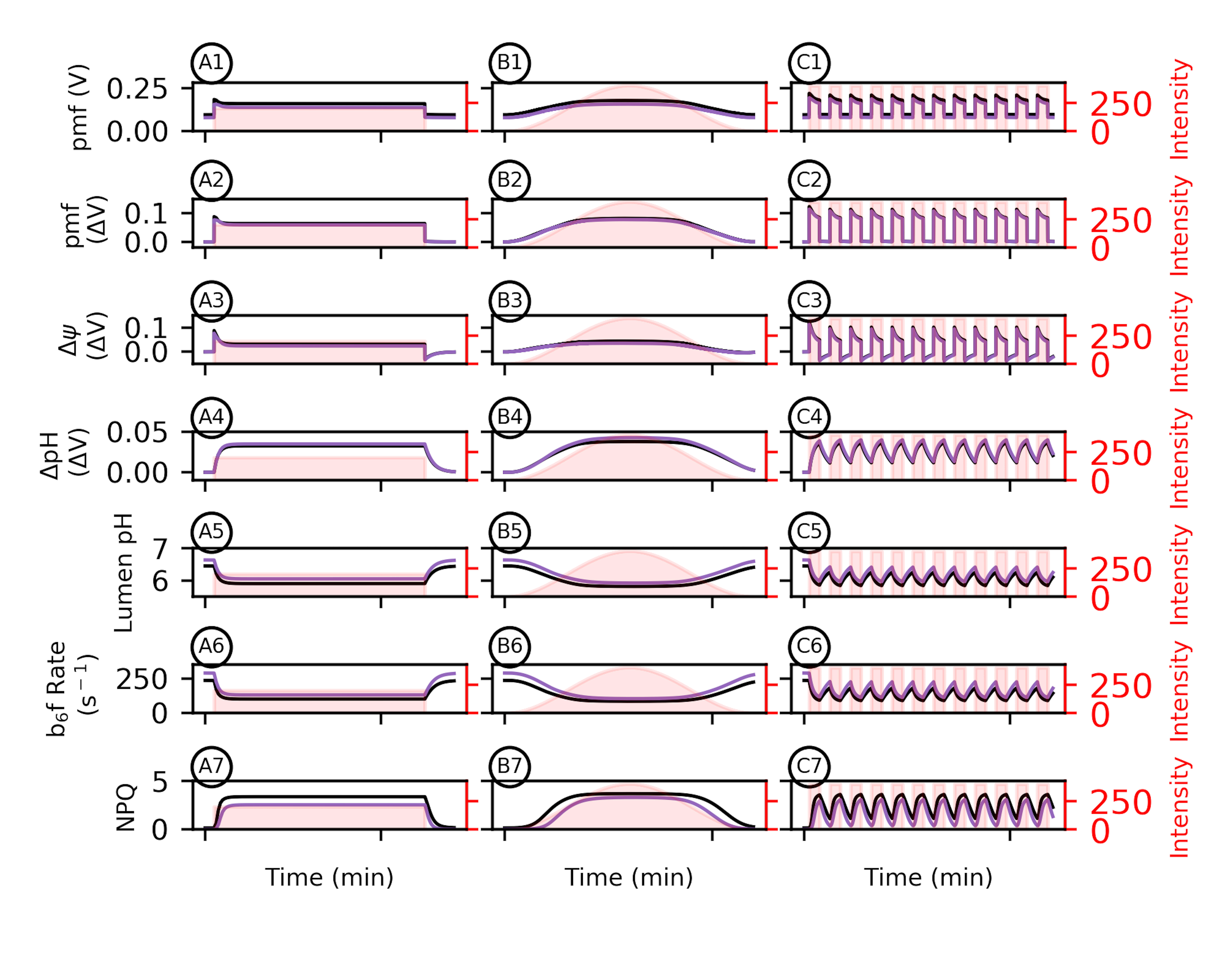

Supplement: Supplementary Figure 7 — Altered ATP synthase c 17 stoichiometry impacts pmf composition and pH–mediated regulatory processes during photosynthesis under increasingly dynamic light environments. Simulated responses of the light reactions were performed as in Davis et al., 2017, with all standard conditions held constant except for the number of ATP synthase c-subunits. Simulations were performed using 1-hour of either static light (A), sinusoidal light (B), or square wave fluctuating light (C) with equal total photon flux over the total duration of each light treatment. Intervals of light excitation are indicated by shaded regions. (Panels 1-4) The light–induced pmf of ATP synthases with c–stoichiometries of 17 (purple) or 14 (black) are shown in units of volts, so that a ΔpH of one is equivalent to 0.06 V. The total pmf (panel 2), Δψ (panel 3), and ΔpH (panel 4) are shown as light–induced changes relative to the pmf dark values indicated as ΔV from dark values, to emphasize light–induced ATP synthase constraints. (5) Light–induced changes in lumen pH due to photosynthetic activity. Light intensities and c–ring composition as in (1). (6) The relative rate constant for plastoquinol oxidation at the cytochrome b6f complex and (7) the extent of nonphotochemical quenching qE component for each c–ring size due to the light–induced changes in lumen pH. [file Image_7.tiff]

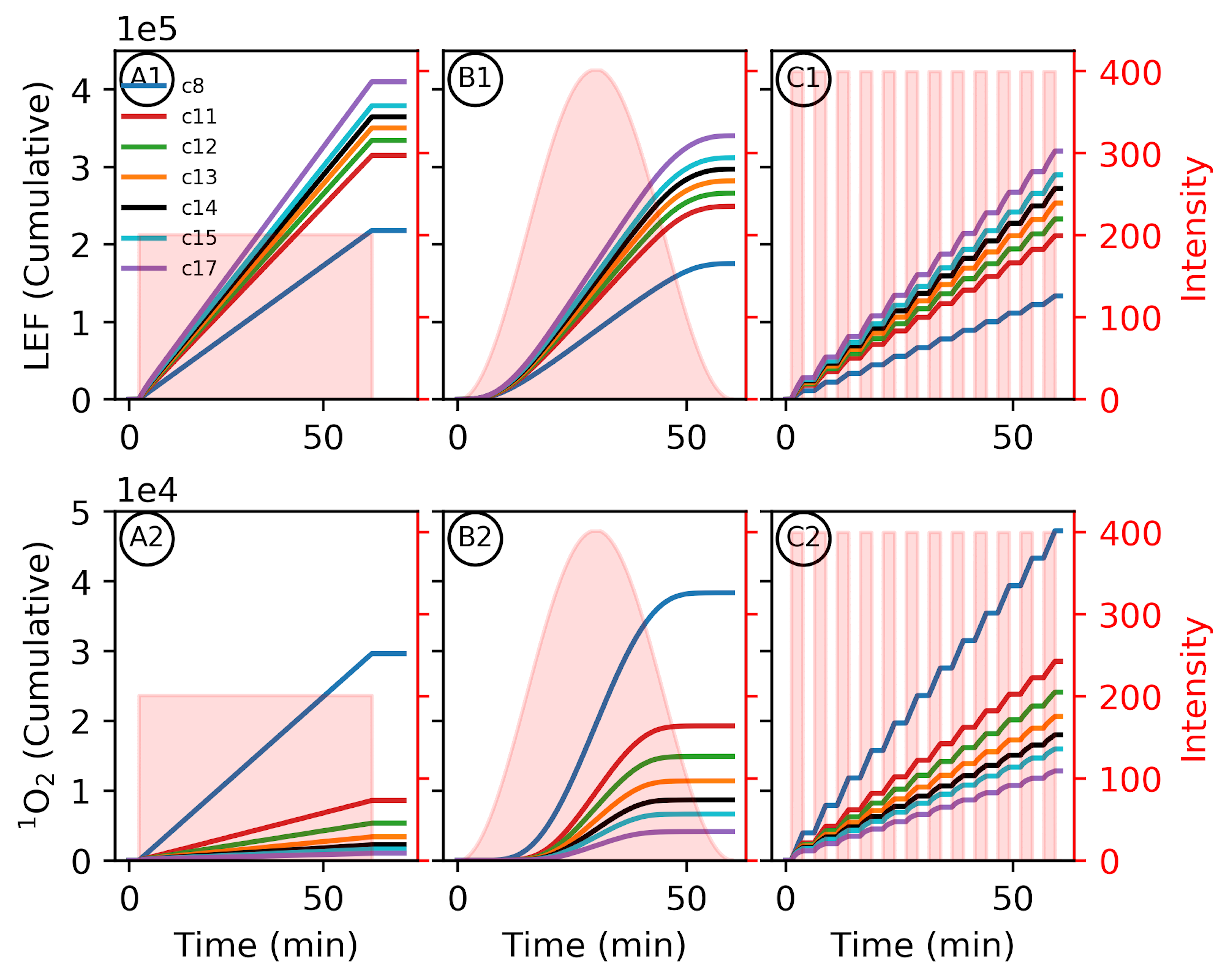

Supplement: Supplementary Figure 8 — Altered pmf composition due to c-subunit stoichiometry limits photosynthetic productivity. Simulated responses of the light reactions were performed as in figures 3 and 4. Variability in environment was simulated with 1-hour light profiles of static light (A1, 2), sinusoidal light (B1, 2), or square wave fluctuating light (C1, 2) to provide the same total illumination during the simulation. (1) The total outputs for linear electron flow (LEF) over the course of the light simulations and (2) 1O2 were integrated over the light treatment to give the cumulative totals. Shaded regions indicate the light profiles for each simulation. [file Image_8.tiff]
