# Supplementary material for: Optimization of ATP Synthase c–Rings for Oxygenic Photosynthesis
Source: Front Plant Sci. 2020 Jan 30;10:1778. doi: 10.3389/fpls.2019.01778 (PMC7003800; doi:10.3389/fpls.2019.01778)
Supplement: Supplementary file 9 [file DataSheet_1.pdf]

In [1]:

```
%matplotlib notebook
```

In [2]:

```
#The following loads the bulk of the hard core code that runs th
e simulations
#before running this you must install the delta_psi_py package:
#see https://github.com/protonzilla/Delta_Psi_Py for information
on downloading
#Davis, GA, Rutherford, AW, and Kramer, DM. (2017)
#Hacking the thylakoid proton motive force for improved photosyn
thesis:
#modulating ion flux rates that control proton motive force part
itioning into Δψ and ΔpH.
#Philosophical transactions of the Royal Society of London Serie
s B, Biological sciences 372.

from delta_psi_py import *
```

In [3]:

```
#Using the standard conditions, set up in the main library, the
initial_sim_states is a
#class that contains the standard initial values. To insert thes
e values into the simulations,
#it is necessary to convert to a list, using the method .as_list
(), as in the following:
# initial_sim_states.as_list()

initial_sim_states=sim_states()
initial_sim_state_list=initial_sim_states.as_list()

Kx_initial=sim_constants()
Kx_initial.k_KEA=0
Kx_initial.fraction_pH_effect=.25
#perform an initial simulation to allow the system to reach equi
librium in the dark


original_dark_equilibrated_initial_y, output=dark_equibration(in
itial_sim_states.as_list(),
                                                              Kx
_initial, 60*60, return_kinetics=True)
#All_Constants_Table('Standard Constants', Kx_initial)
```

In [4]:

```
#All_Constants_Table('Standard Initial States', initial_sim_stat
es)
```

In [5]:

```
#generate a dictionary to hold the results of the simulations.

output_dict={}
constants_dict={}
starting_conditions_dict={}
```

In [6]:

```
#import pandas as pd to manipulate dataframes
import pandas as pd

#generate a dictionary of c-subunit stoichiometries and associat
ed colors
set_of_c_stoichiometries = {8:'tab:blue',11:'tab:red',12:'tab:gr
een',13:'tab:orange',
                            14:'black',15:'tab:cyan',17:'tab:pur
ple'}
```

In [7]:

```
#generate a dictionary of light intensities and associated lines
tyles
flat_light_intensities = {20:'-',100:'--',1000:':'}
```

In [8]:

```
#generate a 10 minute light pulse for each of the 3 intensities
in the flat_light_intensities dictionary

for intensity in flat_light_intensities:
    baseline_duration=150 #in seconds
    baseline_intensity=0 #dark baseline intensity
    pulse_duration=600 #600 seconds (10 minute) pulse
    pulse_intensity=intensity #pulse is X intensity units
    recovery_duration = 500 #500 seconds recovery
    recovery_intensity=0 #recovery is dark
    rise_time=1 #1000 ms for the light to rise
    time_units='seconds'
    point_frequency=100 #start with a frequency of 100 points pe
r subtrace
    repeat_cycles=1 #do this once
    wave=generate_square_wave_based_light_sequence (baseline_dur
ation, baseline_intensity,
                        pulse_duration, pulse_intensity, recover
y_duration, recovery_intensity,
                        rise_time, time_units, point_frequency,
repeat_cycles)
    light_pattern['single_square_10_min_pulse_'+str(intensity)+'
_max']=wave
```

In [9]:

```
#run simulations for each of the flat_light_intensities
#all standard conditions remain unchanged
#vary n (H+/ATP) based on each c-subunit stoichiometry in set_of
_c_stoichiometries dictionary

for intensity in flat_light_intensities:
    print(intensity)
    for c_stoichiometry in set_of_c_stoichiometries:
        n = c_stoichiometry/3

        #the output dictionary name
        on='single 10 min '+str(intensity)+' light pulse c subun
its='+str(c_stoichiometry)
        print('n='+str(n))
        print(on)
        Kx=sim_constants() #generrate arrays contining optimized
time segments for the simulation
        Kx.k_KEA=0
        Kx.fraction_pH_effect=.25

        Kx.n=n
        constants_dict[on]=Kx #store constants in constants_dict

        output_dict[on], starting_conditions_dict[on]=sim(Kx, or
iginal_dark_equilibrated_initial_y,
                                                  light_pattern['s
ingle_square_10_min_pulse_'+str(intensity)+'_max'],
                                                  max_light_change
, points_per_segment, dark_equilibration=60*60)

        Changed_Constants_Table('Change Constants', Kx_initial,
Kx)
```

```
20
n=2.6666666666666665
single 10 min 20 light pulse c subunits=8


Change Constants
```

Changed Parameter	Old Value	New Value
light_per_L	0.0	0.6060606060606055
n	4.666666666666667	2.6666666666666665

n=3.6666666666666665
single 10 min 20 light pulse c subunits=11

Change Constants

Changed Parameter	Old Value	New Value
light_per_L	0.0	0.6060606060606055
n	4.666666666666667	3.6666666666666665

n=4.0
single 10 min 20 light pulse c subunits=12

Change Constants

Changed Parameter	Old Value	New Value
light_per_L	0.0	0.6060606060606055
n	4.666666666666667	4.0

n=4.333333333333333
single 10 min 20 light pulse c subunits=13

Change Constants

Changed Parameter	Old Value	New Value
light_per_L	0.0	0.6060606060606055
n	4.666666666666667	4.333333333333333

n=4.666666666666667
single 10 min 20 light pulse c subunits=14

Change Constants

Changed Parameter	Old Value	New Value
light_per_L	0.0	0.6060606060606055

```
n=5.0
single 10 min 20 light pulse c subunits=15
```

```
Change Constants
```

Changed Parameter	Old Value	New Value
light_per_L	0.0	0.6060606060606055
n	4.666666666666667	5.0

```
n=5.666666666666667
single 10 min 20 light pulse c subunits=17
```

```
Change Constants
```

Changed Parameter	Old Value	New Value
light_per_L	0.0	0.6060606060606055
n	4.666666666666667	5.666666666666667

```
100
n=2.6666666666666665
single 10 min 100 light pulse c subunits=8
```

```
Change Constants
```

Changed Parameter	Old Value	New Value
n	4.666666666666667	2.6666666666666665

```
n=3.6666666666666665
single 10 min 100 light pulse c subunits=11
```

```
Change Constants
```

Changed Parameter	Old Value	New Value
n	4.666666666666667	3.6666666666666665

n=4.0
single 10 min 100 light pulse c subunits=12

Change Constants

Changed Parameter	Old Value	New Value
n	4.666666666666667	4.0

n=4.333333333333333
single 10 min 100 light pulse c subunits=13

Change Constants

Changed Parameter	Old Value	New Value
n	4.666666666666667	4.333333333333333

n=4.666666666666667
single 10 min 100 light pulse c subunits=14

Change Constants

Changed Parameter	Old Value	New Value

n=5.0
single 10 min 100 light pulse c subunits=15

Change Constants

Changed Parameter	Old Value	New Value
n	4.666666666666667	5.0

n=5.666666666666667
single 10 min 100 light pulse c subunits=17

Change Constants

Changed Parameter	Old Value	New Value
n	4.666666666666667	5.666666666666667

1000
n=2.6666666666666665
single 10 min 1000 light pulse c subunits=8

Change Constants

Changed Parameter	Old Value	New Value
n	4.666666666666667	2.6666666666666665

n=3.6666666666666665
single 10 min 1000 light pulse c subunits=11

Change Constants

Changed Parameter	Old Value	New Value
n	4.666666666666667	3.6666666666666665

n=4.0
single 10 min 1000 light pulse c subunits=12

Change Constants

Changed Parameter	Old Value	New Value
n	4.666666666666667	4.0

n=4.333333333333333
single 10 min 1000 light pulse c subunits=13

Change Constants

Changed Parameter	Old Value	New Value
n	4.666666666666667	4.333333333333333

```
n=4.666666666666667
single 10 min 1000 light pulse c subunits=14


Change Constants
```

Changed Parameter	Old Value	New Value

```
n=5.0
single 10 min 1000 light pulse c subunits=15


Change Constants
```

Changed Parameter	Old Value	New Value
n	4.666666666666667	5.0

```
n=5.666666666666667
single 10 min 1000 light pulse c subunits=17


Change Constants
```

Changed Parameter	Old Value	New Value
n	4.666666666666667	5.666666666666667

In [11]:

```
#generate a figure of lumen pH-mediated conditions at equilibrium with deltaG ATP
print('Figure 2: ATP synthase c-ring size impacts photosynthetic physiology in the dark')

fig = plt.figure('Figure 2', figsize=(5,4), dpi=200)
ax1 = fig.add_subplot(311)
ax2 = fig.add_subplot(312)
ax3 = fig.add_subplot(313)
ax4 = ax3.twiny()
```

```
n = []

for key in output_dict:
    c=key.split('=')[1]
    if "single 10 min" in str(key):
        ax1.bar(c,output_dict[key]['pHlumen'][0], color=set_of_c
_stoichiometries[int(c)])
        ax2.bar(c,output_dict[key]['b6f_control'][0],color=set_o
f_c_stoichiometries[int(c)])
        ax3.bar(c,output_dict[key]['NPQ'][0],color=set_of_c_stoi
chiometries[int(c)])
        ax4.bar(c,output_dict[key]['NPQ'][0],color=set_of_c_stoi
chiometries[int(c)])
    values = round(int(c)/3,2)
    n.append(values)

ax1.set_ylabel('Lumen pH')
ax2.set_ylabel('b$_6$f Rate\nConstant (s$^-$$^1$)')
ax3.set_ylabel('NPQ')
ax3.set_xlabel(r'$c$-ring size')
ax1.get_xaxis().set_visible(False)
ax2.get_xaxis().set_visible(False)
ax1.set_ylim(bottom=5,top=7)
ax2.set_ylim(bottom=0,top=325)
ax3.set_ylim(bottom=0,top=4)


props = dict(boxstyle='circle', facecolor='white')
ax1.text(.015, .95, 'A', transform=ax1.transAxes, fontsize=8,ver
ticalalignment='top', bbox=props)
ax2.text(.015, .95, 'B', transform=ax2.transAxes, fontsize=8,ver
ticalalignment='top', bbox=props)
ax3.text(.015, .95, 'C', transform=ax3.transAxes, fontsize=8,ver
ticalalignment='top', bbox=props)
ax4.set_xlabel('H$^+$/ATP')
ax4.xaxis.set_ticks_position("bottom")
ax4.xaxis.set_label_position("bottom")
ax4.spines["bottom"].set_position(("axes", -0.55))
labels = ['2.67','3.67','4.0','4.33','4.67','5.0','5.67']
ax4.set_xticklabels(labels)
plt.tight_layout(pad=0.4, w_pad=0.5, h_pad=.0)
plt.show()
print('Kinetic modelling of photosynthetic light reactions with
altered ATP synthase c subunit stoichiometry. '
      'Simulated responses of the light reactions were performed
[illegible]
```

```
as in Davis et al., 2017, with all standard '
      'conditions held constant except for the number of ATP syn
thase c subunits. The pmf required to maintain '
      'equilibrium with ΔGATP in the dark is variable depending
upon the number of c subunits in the ATP synthase '
      'c—ring (Eq. 2). Changes in lumen pH in the dark due to al
terations in c—ring size (A) can decrease '
      'cytochrome b6f turnover rate (B) as well as activate pH—d
ependent NPQ in higher plants (C).')
```

Figure 2: ATP synthase c—ring size impacts photosynthetic physiology in the dark

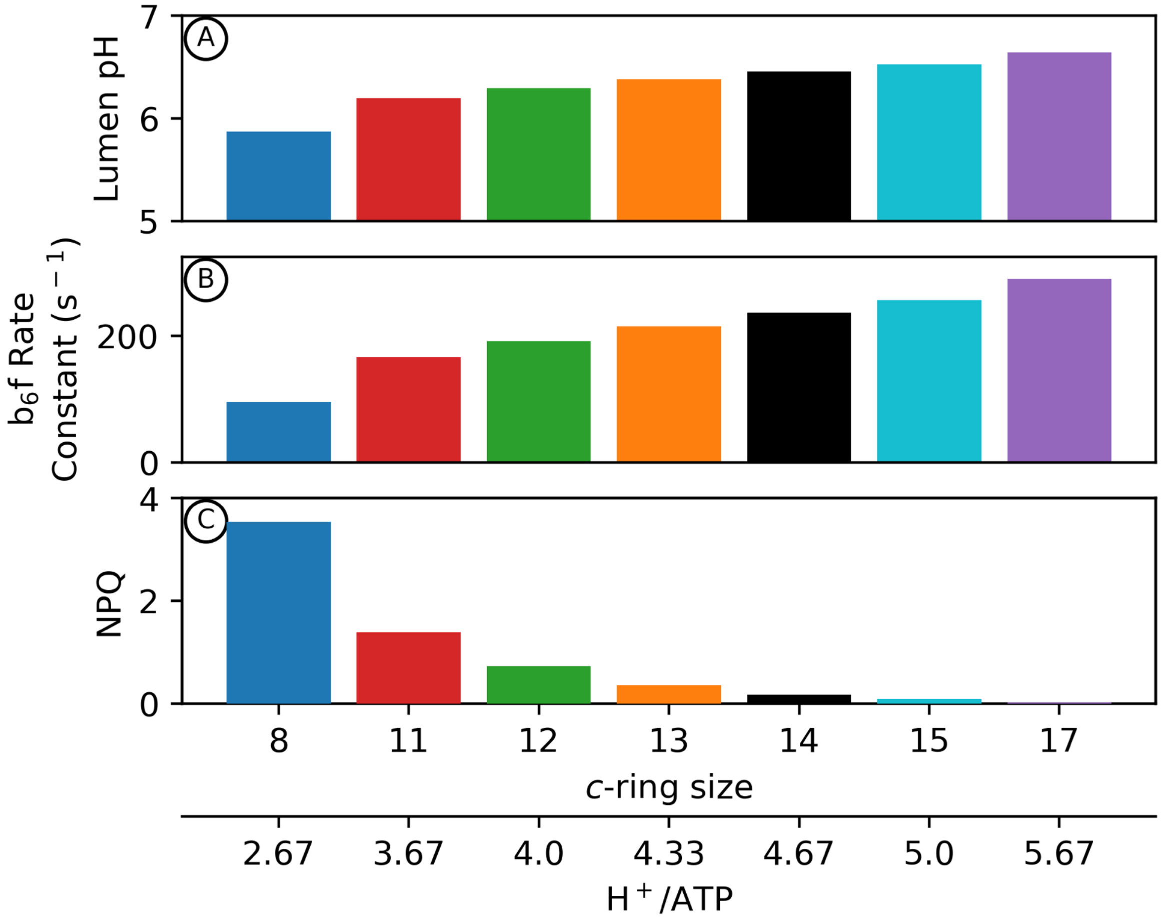

Kinetic modelling of photosynthetic light reactions with altered ATP synthase c subunit stoichiometry. S imulated responses of the light reactions were perfo rmed as in Davis et al., 2017, with all standard con ditions held constant except for the number of ATP s ynthase c subunits. The pmf required to maintain equ ilibrium with ΔGATP in the dark is variable dependin g upon the number of c subunits in the ATP synthase c–ring (Eq. 2). Changes in lumen pH in the dark due to alterations in c–ring size (A) can decrease cytoc hrome b6f turnover rate (B) as well as activate pH–d ependent NPQ in higher plants (C).

In [12]:

```
print('Figure 3: Altered ATP synthase c-subunit stoichiometry li
mits pmf composition and pH–mediated regulatory '
      'processes during photosynthesis.')
fig = plt.figure('Figure 3', figsize=(5,4), dpi=200)
ax1 = fig.add_subplot(7,5,1)
ax2 = fig.add_subplot(7,5,2)
ax3 = fig.add_subplot(7,5,3)
ax4 = fig.add_subplot(7,5,4)
ax5 = fig.add_subplot(7,5,5)
ax6 = fig.add_subplot(7,5,6)
ax7 = fig.add_subplot(7,5,7)
ax8 = fig.add_subplot(7,5,8)
ax9 = fig.add_subplot(7,5,9)
ax10 = fig.add_subplot(7,5,10)
ax11 = fig.add_subplot(7,5,11)
ax12 = fig.add_subplot(7,5,12)
ax13 = fig.add_subplot(7,5,13)
ax14 = fig.add_subplot(7,5,14)
ax15 = fig.add_subplot(7,5,15)
ax16 = fig.add_subplot(7,5,16)
ax17 = fig.add_subplot(7,5,17)
ax18 = fig.add_subplot(7,5,18)
ax19 = fig.add_subplot(7,5,19)
ax20 = fig.add_subplot(7,5,20)
ax21 = fig.add_subplot(7,5,21)
ax22 = fig.add_subplot(7,5,22)
ax23 = fig.add_subplot(7,5,23)
ax24 = fig.add_subplot(7,5,24)
ax25 = fig.add_subplot(7,5,25)
```

```
ax26 = fig.add_subplot(7,5,26)
ax27 = fig.add_subplot(7,5,27)
ax28 = fig.add_subplot(7,5,28)
ax29 = fig.add_subplot(7,5,29)
ax30 = fig.add_subplot(7,5,30)
ax31 = fig.add_subplot(7,5,31)
ax32 = fig.add_subplot(7,5,32)
ax33 = fig.add_subplot(7,5,33)
ax34 = fig.add_subplot(7,5,34)
ax35 = fig.add_subplot(7,5,35)


ax1b = ax1.twinx()
ax2b = ax2.twinx()
ax3b = ax3.twinx()
ax4b = ax4.twinx()
ax5b = ax5.twinx()
ax6b = ax6.twinx()
ax7b = ax7.twinx()
ax8b = ax8.twinx()
ax9b = ax9.twinx()
ax10b = ax10.twinx()
ax11b = ax11.twinx()
ax12b = ax12.twinx()
ax13b = ax13.twinx()
ax14b = ax14.twinx()
ax15b = ax15.twinx()
ax16b = ax16.twinx()
ax17b = ax17.twinx()
ax18b = ax18.twinx()
ax19b = ax19.twinx()
ax20b = ax20.twinx()
ax21b = ax21.twinx()
ax22b = ax22.twinx()
ax23b = ax23.twinx()
ax24b = ax24.twinx()
ax25b = ax25.twinx()
ax26b = ax26.twinx()
ax27b = ax27.twinx()
ax28b = ax28.twinx()
ax29b = ax29.twinx()
ax30b = ax30.twinx()
ax31b = ax31.twinx()
ax32b = ax32.twinx()
ax33b = ax33.twinx()
```

```
ax34b = ax34.twinx()
ax35b = ax35.twinx()

axes_row1 = [ax1,ax2,ax3,ax4,ax5]
axes_row2 = [ax6,ax7,ax8,ax9,ax10]
axes_row3 = [ax11,ax12,ax13,ax14,ax15]
axes_row4 = [ax16,ax17,ax18,ax19,ax20]
axes_row5 = [ax21,ax22,ax23,ax24,ax25]
axes_row6 = [ax26,ax27,ax28,ax29,ax30]
axes_row7 = [ax31,ax32,ax33,ax34,ax35]
c_stoichiometry = ['8','11','12','14','17']


for key in output_dict:
    if "single 10 min" in str(key):
        light=key.split('single 10 min ')[1]
        light=light.split(' light')[0]

        for i in range(len(axes_row1)):
            subplot_row1 = axes_row1[i]
            subplot_row2 = axes_row2[i]
            subplot_row3 = axes_row3[i]
            subplot_row4 = axes_row4[i]
            subplot_row5 = axes_row5[i]
            subplot_row6 = axes_row6[i]
            subplot_row7 = axes_row7[i]
            c = c_stoichiometry[i]
            if c == key.split('=')[1]:
                subplot_row1.plot(output_dict[key]['time_axis']/
60,output_dict[key]['pmf'],
                        color=set_of_c_stoichiometries[int(c)],l
inestyle=flat_light_intensities[int(light)])
                subplot_row2.plot(output_dict[key]['time_axis']/
60,output_dict[key]['pmf_offset'],
                        color=set_of_c_stoichiometries[int(c)],l
inestyle=flat_light_intensities[int(light)])
                subplot_row3.plot(output_dict[key]['time_axis']/
60,output_dict[key]['Dy_offset'],
                        color=set_of_c_stoichiometries[int(c)],l
inestyle=flat_light_intensities[int(light)])
                subplot_row4.plot(output_dict[key]['time_axis']/
60,output_dict[key]['delta_pH_V_offset'],
                        color=set_of_c_stoichiometries[int(c)],l
inestyle=flat_light_intensities[int(light)])
                subplot_row5.plot(output_dict[key]['time_axis']/
```

```
60,output_dict[key]['pHlumen'],

                                color=set_of_c_stoichiometries[int(c)],l
inestyle=flat_light_intensities[int(light)])
                    subplot_row6.plot(output_dict[key]['time_axis']/
60,output_dict[key]['b6f_control'],
                                color=set_of_c_stoichiometries[int(c)],l
inestyle=flat_light_intensities[int(light)])
                    subplot_row7.plot(output_dict[key]['time_axis']/
60,output_dict[key]['NPQ_array'],
                                color=set_of_c_stoichiometries[int(c)],l
inestyle=flat_light_intensities[int(light)])

axes = [ax1b,ax2b,ax3b,ax4b,ax5b,ax6b,ax7b,ax8b,ax9b,ax10b,ax11b
,ax12b,ax13b,ax14b,ax15b,ax16b,ax17b,ax18b,
        ax19b,ax20b,ax21b,ax22b,ax23b,ax24b,ax25b,ax26b,ax27b,ax
28b,ax29b,ax30b,ax31b,ax32b,ax33b,ax34b,ax35b]

for key in output_dict:
    for i in range(len(axes)):
        subplot=axes[i]
        if "single 10 min" in str(key):
            c=key.split('=')[1]
            light=key.split('single 10 min ')[1]
            light=light.split(' light')[0]
            if light == '1000' and c == '8':
                subplot.fill_between(output_dict[key]['time_axis
']/60, output_dict[key]['light_curve'],0,
                                    color='red', alpha=0.05,zor
der=2)

ax1.set_ylabel('pmf (V)',fontsize=7)
ax6.set_ylabel('pmf\n ($\Delta$V)',fontsize=7)
ax11.set_ylabel('$\Delta$$\psi$\n ($\Delta$V)',fontsize=7)
ax16.set_ylabel('$\Delta$pH\n ($\Delta$V)',fontsize=7)
ax21.set_ylabel('Lumen pH',fontsize=7)
ax26.set_ylabel('b$_6$f Rate\n (s$^-$$^1$)',fontsize=7)
ax31.set_ylabel('NPQ',fontsize=7)


axes = [ax2,ax3,ax4,ax5,ax7,ax8,ax9,ax10,ax12,ax13,ax14,ax15,ax1
7,ax18,ax19,ax20,ax22,ax23,ax24,ax25,ax27,ax28,
        ax29,ax30,ax32,ax33,ax34,ax35,ax1b,ax2b,ax3b,ax4b,ax5b,a
x6b,ax7b,ax8b,ax9b,ax10b,ax11b,ax12b,ax13b,ax14b,
        ax15b,ax16b,ax17b,ax18b,ax19b,ax20b,ax21b,ax22b,ax23b,ax
```

```
24b,ax25b,ax26b,ax27b,ax28b,ax29b,ax30b,ax31b,

        ax32b,ax33b,ax34b,ax35b]
for i in range(len(axes)):
    subplot=axes[i]
    subplot.set_yticklabels([])

axes = [ax1,ax2,ax3,ax4,ax5,ax6,ax7,ax8,ax9,ax10,ax11,ax12,ax13,
ax14,ax15,ax16,
        ax17,ax18,ax19,ax20,ax21,ax22,ax23,ax24,ax25,ax26,ax27,a
x28,ax29,ax30,
        ax1b,ax2b,ax3b,ax4b,ax5b,ax6b,ax7b,ax8b,ax9b,ax10b,ax11b
,ax12b,ax13b,
        ax14b,ax15b,ax16b,ax17b,ax18b,ax19b,ax20b,ax21b,ax22b,ax
23b,ax24b,ax25b,ax26b,ax27b,ax28b,ax29b,ax30b]
for i in range(len(axes)):
    subplot=axes[i]
    subplot.set_xticklabels([])

axes = [ax1b,ax2b,ax3b,ax4b,ax5b,ax6b,ax7b,ax8b,ax9b,ax10b,ax11b
,ax12b,ax13b,
        ax14b,ax15b,ax16b,ax17b,ax18b,ax19b,ax20b,ax21b,ax22b,ax
23b,ax24b,ax25b,ax26b,ax27b,ax28b,ax29b,ax30b,
        ax31b,ax32b,ax33b,ax34b,ax35b]
for i in range(len(axes)):
    subplot=axes[i]
    subplot.tick_params(right='off')


props = dict(boxstyle='circle', facecolor='white')
labels = ['A1','B1','C1','D1','E1']
for i in range(len(axes_row1)):
    subplot=axes_row1[i]
    label=labels[i]
    subplot.text(18, .31, label, fontsize=6,verticalalignment='t
op', bbox=props)
    subplot.set_ylim(bottom=-0.05, top=0.35)
labels = ['A2','B2','C2','D2','E2']
for i in range(len(axes_row2)):
    subplot=axes_row2[i]
    label=labels[i]
    subplot.text(18, .15, label, fontsize=6,verticalalignment='t
op', bbox=props)
    subplot.set_ylim(bottom=-0.03, top=0.17)
labels = ['A3','B3','C3','D3','E3']
```

```
for i in range(len(axes_row3)):

    subplot=axes_row3[i]
    label=labels[i]
    subplot.text(18, .15, label, fontsize=6,verticalalignment='top', bbox=props)
    subplot.set_ylim(bottom=-0.07, top=0.17)
labels = ['A4','B4','C4','D4','E4']
for i in range(len(axes_row4)):
    subplot=axes_row4[i]
    label=labels[i]
    subplot.text(18, .045, label, fontsize=6,verticalalignment='top', bbox=props)
    subplot.set_ylim(bottom=-0.01, top=0.05)
labels = ['A5','B5','C5','D5','E5']
for i in range(len(axes_row5)):
    subplot=axes_row5[i]
    label=labels[i]
    subplot.text(18, 6.85, label, fontsize=6,verticalalignment='top', bbox=props)
    subplot.set_ylim(bottom=5.5, top=7)
labels = ['A6','B6','C6','D6','E6']
for i in range(len(axes_row6)):
    subplot=axes_row6[i]
    label=labels[i]
    subplot.text(18, 310, label, fontsize=6,verticalalignment='top', bbox=props)
    subplot.set_ylim(bottom=0, top=350)

labels = ['A7','B7','C7','D7','E7']
for i in range(len(axes_row7)):
    subplot=axes_row7[i]
    label=labels[i]
    subplot.text(18, 4.5, label, fontsize=6,verticalalignment='top', bbox=props)
    subplot.set_ylim(bottom=0, top=5)
    subplot.set_xlabel('Time (min)',fontsize=7)

axes = [ax1,ax5,ax9,ax13,ax17,ax21]
for i in range(len(axes)):
    subplot=axes[i]
    subplot.tick_params(axis='y',labelsize='small')

axes = [ax1,ax2,ax3,ax4,ax5]
titles = ['c=8','c=11','c=12','c=14','c=17',]
```

```
for i in range(len(axes)):
    subplot=axes[i]
    title=titles[i]
    subplot.set_title(title, size=8)

plt.tight_layout(pad=0.4, w_pad=0.5, h_pad=.5)
plt.show()
print('Simulated responses of the light reactions were performed as in Davis et al., 2017, with all standard '
      'conditions held constant except for the number of ATP synthase c-subunits. Simulations were performed using '
      '10 minutes of static light at either 20 (solid lines), 100 (dashed lines), or 1000 (dotted lines) µmol '
      'photons m-2s-1. Intervals of light excitation are indicated by shaded regions. (Panels 1-4) The light-'
      'induced pmf (1, 2A-D) of ATP synthases with c-stoichiometries of 8 (blue, column A), 11 (red, column B), '
      '12 (green, column C), 14 (black, column D), or 17 (purple, column E) are shown in units of volts, so that '
      'a ΔpH of one is equivalent to 0.06 V. The total pmf (panel 2), Δψ (panel 3), and ΔpH (panel 4) are shown as '
      'light-induced changes relative to the pmf dark values indicated as ΔV from dark values, to emphasize light-'
      'induced ATP synthase constraints. (5) Light-induced changes in lumen pH due to photosynthetic activity. '
      'Light intensities and c-ring composition as in (1). (6) The relative rate constant for plastoquinol '
      'oxidation at the cytochrome b6f complex and (7) the extent of nonphotochemical quenching qE component for '
      'each c-ring size due to the light-induced changes in lumen pH. ')
```

Figure 3: Altered ATP synthase c-subunit stoichiometry limits pmf composition and pH-mediated regulatory processes during photosynthesis.

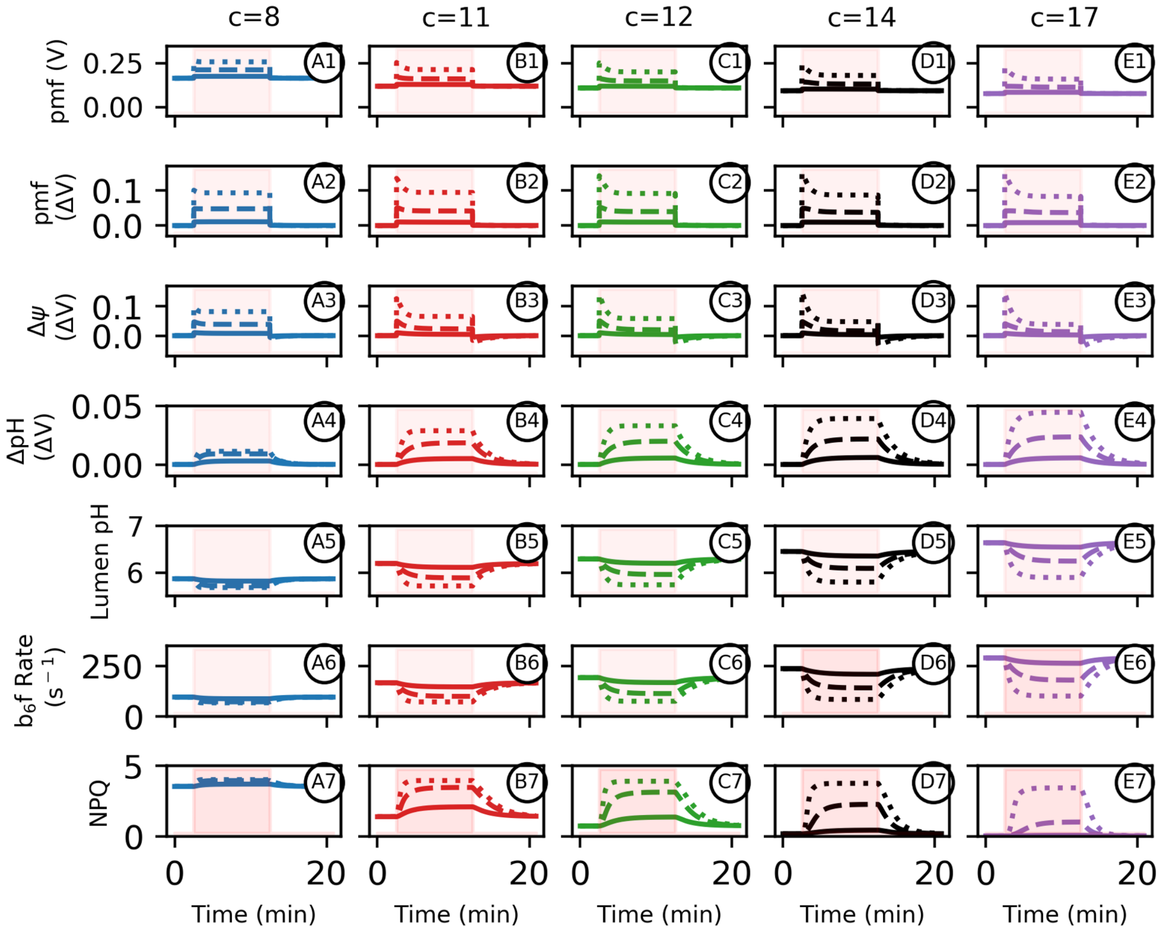
c=8
c=11
c=12
c=14
c=17
pmf (V)
0.25
0.00
pmf (ΔV)
0.1
0.0
Δψ (ΔV)
0.1
0.0
ΔpH (ΔV)
0.05
0.00
Lumen pH
7
6
b6f Rate (s−1)
250
0
NPQ
5
0
A1
A2
A3
A4
A5
A6
A7
B1
B2
B3
B4
B5
B6
B7
C1
C2
C3
C4
C5
C6
C7
D1
D2
D3
D4
D5
D6
D7
E1
E2
E3
E4
E5
E6
E7
0
20
Time (min)

Simulated responses of the light reactions were performed as in Davis et al., 2017, with all standard conditions held constant except for the number of ATP synthase c-subunits. Simulations were performed using 10 minutes of static light at either 20 (solid lines), 100 (dashed lines), or 1000 (dotted lines) μmol photons m-2s-1. Intervals of light excitation are indicated by shaded regions. (Panels 1-4) The light–induced pmf (1, 2A-D) of ATP synthases with c–stoichiometries of 8 (blue, column A), 11 (red, column B), 12 (green, column C), 14 (black, column D), or 17 (purple, column E) are shown in units of volts, so that a ΔpH of one is equivalent to 0.06 V. The total pmf (panel 2), Δψ (panel 3), and ΔpH (panel 4) are shown as light–induced changes relative to the pmf dark values indicated as ΔV from dark values, to emphasize light–induced ATP synthase constraints. (5) Light–induced changes in lumen pH due to photosynthetic activity. Light intensities and c–ring composition as in (1). (6) The relative rate constant for plastoquinol oxidation at the cytochrome b6f complex and (7) the extent of nonphotochemical quenching qE component for each c–ring size due to the light–induced changes in lumen pH.

In [13]:

```
print('Supplemental Figure 1: Comparison of pmf composition and
pH–mediated regulatory processes during '
      'photosynthesis in naturally occuring photosynthetic c-rin
gs and c12 ring to balance ATP/NADPH with '
      'carbon assimilation.')
fig = plt.figure('Supplemental Figure 1', figsize=(5,4), dpi=200
)
ax1 = fig.add_subplot(7,4,1)
ax2 = fig.add_subplot(7,4,2)
ax3 = fig.add_subplot(7,4,3)
ax4 = fig.add_subplot(7,4,4)
ax5 = fig.add_subplot(7,4,5)
ax6 = fig.add_subplot(7,4,6)
ax7 = fig.add_subplot(7,4,7)
ax8 = fig.add_subplot(7,4,8)
ax9 = fig.add_subplot(7,4,9)
ax10 = fig.add_subplot(7,4,10)
ax11 = fig.add_subplot(7,4,11)
```

```
ax11 = fig.add_subplot(7,4,11)
ax12 = fig.add_subplot(7,4,12)

ax13 = fig.add_subplot(7,4,13)
ax14 = fig.add_subplot(7,4,14)
ax15 = fig.add_subplot(7,4,15)
ax16 = fig.add_subplot(7,4,16)
ax17 = fig.add_subplot(7,4,17)
ax18 = fig.add_subplot(7,4,18)
ax19 = fig.add_subplot(7,4,19)
ax20 = fig.add_subplot(7,4,20)
ax21 = fig.add_subplot(7,4,21)
ax22 = fig.add_subplot(7,4,22)
ax23 = fig.add_subplot(7,4,23)
ax24 = fig.add_subplot(7,4,24)
ax25 = fig.add_subplot(7,4,25)
ax26 = fig.add_subplot(7,4,26)
ax27 = fig.add_subplot(7,4,27)
ax28 = fig.add_subplot(7,4,28)


ax1b = ax1.twinx()
ax2b = ax2.twinx()
ax3b = ax3.twinx()
ax4b = ax4.twinx()
ax5b = ax5.twinx()
ax6b = ax6.twinx()
ax7b = ax7.twinx()
ax8b = ax8.twinx()
ax9b = ax9.twinx()
ax10b = ax10.twinx()
ax11b = ax11.twinx()
ax12b = ax12.twinx()
ax13b = ax13.twinx()
ax14b = ax14.twinx()
ax15b = ax15.twinx()
ax16b = ax16.twinx()
ax17b = ax17.twinx()
ax18b = ax18.twinx()
ax19b = ax19.twinx()
ax20b = ax20.twinx()
ax21b = ax21.twinx()
ax22b = ax22.twinx()
ax23b = ax23.twinx()
ax24b = ax24.twinx()
ax25b = ax25.twinx()
```

```
ax26b = ax26.twinx()
ax27b = ax27.twinx()
ax28b = ax28.twinx()

axes_row1 = [ax1,ax2,ax3,ax4]
axes_row2 = [ax5,ax6,ax7,ax8]
axes_row3 = [ax9,ax10,ax11,ax12]
axes_row4 = [ax13,ax14,ax15,ax16]
axes_row5 = [ax17,ax18,ax19,ax20]
axes_row6 = [ax21,ax22,ax23,ax24]
axes_row7 = [ax25,ax26,ax27,ax28]
c_stoichiometry = ['12','13','14','15']


for key in output_dict:
    if "single 10 min" in str(key):
        light=key.split('single 10 min ')[1]
        light=light.split(' light')[0]

        for i in range(len(axes_row1)):
            subplot_row1 = axes_row1[i]
            subplot_row2 = axes_row2[i]
            subplot_row3 = axes_row3[i]
            subplot_row4 = axes_row4[i]
            subplot_row5 = axes_row5[i]
            subplot_row6 = axes_row6[i]
            subplot_row7 = axes_row7[i]
            c = c_stoichiometry[i]
            if c == key.split('=')[1]:
                subplot_row1.plot(output_dict[key]['time_axis']/
60,output_dict[key]['pmf'],
                        color=set_of_c_stoichiometries[int(c)],l
inestyle=flat_light_intensities[int(light)])
                subplot_row2.plot(output_dict[key]['time_axis']/
60,output_dict[key]['pmf_offset'],
                        color=set_of_c_stoichiometries[int(c)],l
inestyle=flat_light_intensities[int(light)])
                subplot_row3.plot(output_dict[key]['time_axis']/
60,output_dict[key]['Dy_offset'],
                        color=set_of_c_stoichiometries[int(c)],l
inestyle=flat_light_intensities[int(light)])
                subplot_row4.plot(output_dict[key]['time_axis']/
60,output_dict[key]['delta_pH_V_offset'],
                        color=set_of_c_stoichiometries[int(c)],l
inestyle=flat_light_intensities[int(light)])
```

```
                subplot_row5.plot(output_dict[key]['time_axis']/
60,output_dict[key]['pHlumen'],
                        color=set_of_c_stoichiometries[int(c)],l
inestyle=flat_light_intensities[int(light)])
                subplot_row6.plot(output_dict[key]['time_axis']/
60,output_dict[key]['b6f_control'],
                        color=set_of_c_stoichiometries[int(c)],l
inestyle=flat_light_intensities[int(light)])
                subplot_row7.plot(output_dict[key]['time_axis']/
60,output_dict[key]['NPQ_array'],
                        color=set_of_c_stoichiometries[int(c)],l
inestyle=flat_light_intensities[int(light)])

axes = [ax1b,ax2b,ax3b,ax4b,ax5b,ax6b,ax7b,ax8b,ax9b,ax10b,ax11b
,ax12b,ax13b,ax14b,ax15b,ax16b,ax17b,ax18b,ax19b,
        ax20b,ax21b,ax22b,ax23b,ax24b,ax25b,ax26b,ax27b,ax28b,ax
29b,ax30b,ax31b,ax32b,ax33b,ax34b,ax35b]

for key in output_dict:
    for i in range(len(axes)):
        subplot=axes[i]
        if "single 10 min" in str(key):
            c=key.split('=')[1]
            light=key.split('single 10 min ')[1]
            light=light.split(' light')[0]
            if light == '1000' and c == '8':
                subplot.fill_between(output_dict[key]['time_axis
']/60, output_dict[key]['light_curve'],0,
                                     color='red', alpha=0.05,zor
der=2)

ax1.set_ylabel('pmf (V)',fontsize=7)
ax5.set_ylabel('pmf\n ($\Delta$V)',fontsize=7)
ax9.set_ylabel('$\Delta$$\psi$\n ($\Delta$V)',fontsize=7)
ax13.set_ylabel('$\Delta$pH\n ($\Delta$V)',fontsize=7)
ax17.set_ylabel('Lumen pH',fontsize=7)
ax21.set_ylabel('b$_6$f Rate\n (s$^-$$^1$)',fontsize=7)
ax25.set_ylabel('NPQ',fontsize=7)


axes = [ax2,ax3,ax4,ax6,ax7,ax8,ax10,ax11,ax12,ax14,ax15,ax16,ax
18,ax19,ax20,ax22,ax23,ax24,ax26,ax27,ax28,
        ax1b,ax2b,ax3b,ax4b,ax5b,ax6b,ax7b,ax8b,ax9b,ax10b,ax11b
,ax12b,ax13b,
        ax14b,ax15b,ax16b,ax17b,ax18b,ax19b,ax20b,ax21b,ax22b,ax
```

```
23b,ax24b,ax25b,ax26b,ax27b,ax28b]
for i in range(len(axes)):
    subplot=axes[i]
    subplot.set_yticklabels([])

axes = [ax1,ax2,ax3,ax4,ax5,ax6,ax7,ax8,ax9,ax10,ax11,ax12,ax13,
ax14,ax15,ax16,
        ax17,ax18,ax19,ax20,ax21,ax22,ax23,ax24]
for i in range(len(axes)):
    subplot=axes[i]
    subplot.set_xticklabels([])

axes = [ax1b,ax2b,ax3b,ax4b,ax5b,ax6b,ax7b,ax8b,ax9b,ax10b,ax11b
,ax12b,ax13b,
        ax14b,ax15b,ax16b,ax17b,ax18b,ax19b,ax20b,ax21b,ax22b,ax
23b,ax24b,ax25b,ax26b,ax27b,ax28b]
for i in range(len(axes)):
    subplot=axes[i]
    subplot.tick_params(right='off')


props = dict(boxstyle='circle', facecolor='white')
labels = ['A1','B1','C1','D1']
for i in range(len(axes_row1)):
    subplot=axes_row1[i]
    label=labels[i]
    subplot.text(18, .31, label, fontsize=6,verticalalignment='t
op', bbox=props)
    subplot.set_ylim(bottom=-0.05, top=0.35)
labels = ['A2','B2','C2','D2']
for i in range(len(axes_row2)):
    subplot=axes_row2[i]
    label=labels[i]
    subplot.text(18, .15, label, fontsize=6,verticalalignment='t
op', bbox=props)
    subplot.set_ylim(bottom=-0.03, top=0.17)
labels = ['A3','B3','C3','D3']
for i in range(len(axes_row3)):
    subplot=axes_row3[i]
    label=labels[i]
    subplot.text(18, .15, label, fontsize=6,verticalalignment='t
op', bbox=props)
    subplot.set_ylim(bottom=-0.07, top=0.17)
labels = ['A4','B4','C4','D4']
for i in range(len(axes_row4)):
```

```
[illegible]
    subplot=axes_row4[i]
    label=labels[i]
    subplot.text(18, .045, label, fontsize=6,verticalalignment='
top', bbox=props)
    subplot.set_ylim(bottom=-0.01, top=0.05)
labels = ['A5','B5','C5','D5']
for i in range(len(axes_row5)):
    subplot=axes_row5[i]
    label=labels[i]
    subplot.text(18, 6.85, label, fontsize=6,verticalalignment='
top', bbox=props)
    subplot.set_ylim(bottom=5.5, top=7)
labels = ['A6','B6','C6','D6']
for i in range(len(axes_row6)):
    subplot=axes_row6[i]
    label=labels[i]
    subplot.text(18, 310, label, fontsize=6,verticalalignment='t
op', bbox=props)
    subplot.set_ylim(bottom=0, top=350)

labels = ['A7','B7','C7','D7']
for i in range(len(axes_row7)):
    subplot=axes_row7[i]
    label=labels[i]
    subplot.text(18, 4.5, label, fontsize=6,verticalalignment='t
op', bbox=props)
    subplot.set_ylim(bottom=0, top=5)
    subplot.set_xlabel('Time (min)',fontsize=7)

axes = [ax1,ax5,ax9,ax13,ax17,ax21]
for i in range(len(axes)):
    subplot=axes[i]
    subplot.tick_params(axis='y',labelsize='small')

axes = [ax1,ax2,ax3,ax4]
titles = ['c=12','c=13','c=14','c=15']
for i in range(len(axes)):
    subplot=axes[i]
    title=titles[i]
    subplot.set_title(title, size=8)

plt.tight_layout(pad=0.4, w_pad=0.5, h_pad=.5)
plt.show()
print('Simulated responses of the light reactions were performed
as in Davis et al., 2017, with all standard '
```

```
      'conditions held constant except for the number of ATP syn
thase c-subunits. Simulations were performed using '
      '10 minutes of static light at either 20 (solid lines), 10
0 (dashed lines), or 1000 (dotted lines) µmol '
      'photons m-2s-1 as in Figure 1. Intervals of light excitat
ion are indicated by shaded regions. (Panels 1-4) '
      'The light-induced pmf (1, 2A-D) of ATP synthases with c-s
toichiometries of 12 (green, column A), 13 (orange, '
      'column B), 14 (black, column C), or 15 (cyan, column D)ar
e shown in units of volts, so that a ΔpH of one is '
      'equivalent to 0.06 V. The total pmf (panel 2), Δψ (panel
3), and ΔpH (panel 4) are shown as light-'
      'induced changes relative to the pmf dark values indicated
as ΔV from dark values, to emphasize light-induced '
      'ATP synthase constraints. (5) Light-induced changes in lu
men pH due to photosynthetic activity. Light '
      'intensities and c-ring composition as in (1). (6) The rel
ative rate constant for plastoquinol oxidation at '
      'the cytochrome b6f complex and (7) the extent of nonphoto
chemical quenching qE component for each c-ring '
      'size due to the light-induced changes in lumen pH.')
```

Supplemental Figure 1: Comparison of pmf composition and pH-mediated regulatory processes during photosynthesis in naturally occuring photosynthetic c-rings and c12 ring to balance ATP/NADPH with carbon assimilation.

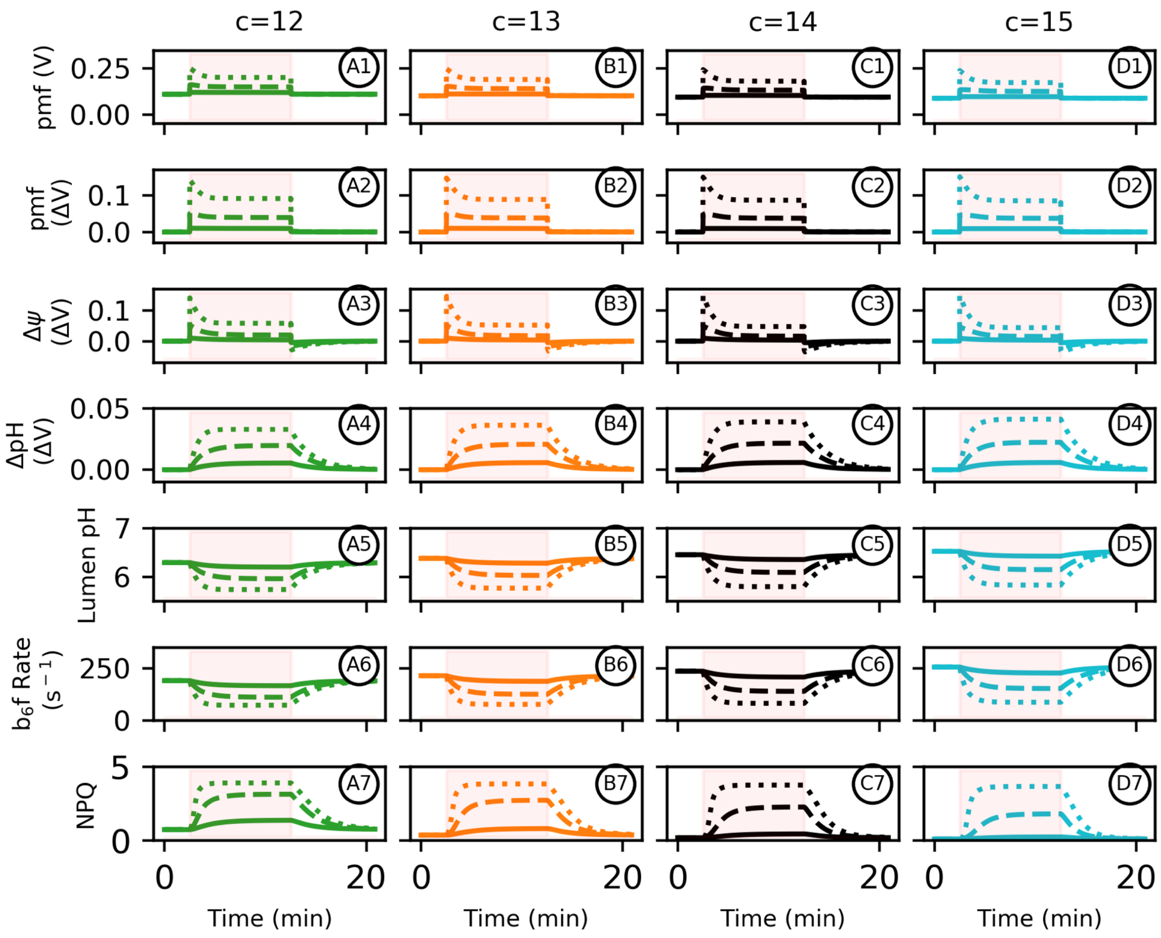

c=12
c=13
c=14
c=15
pmf (V)
0.25
0.00
pmf (ΔV)
0.1
0.0
Δψ (ΔV)
ΔpH (ΔV)
0.05
Lumen pH
7
6
b6f Rate (s−1)
250
0
NPQ
5
A1
B1
C1
D1
A2
B2
C2
D2
A3
B3
C3
D3
A4
B4
C4
D4
A5
B5
C5
D5
A6
B6
C6
D6
A7
B7
C7
D7
20
Time (min)

Simulated responses of the light reactions were performed as in Davis et al., 2017, with all standard conditions held constant except for the number of ATP synthase c-subunits. Simulations were performed using 10 minutes of static light at either 20 (solid lines), 100 (dashed lines), or 1000 (dotted lines) µmol photons m-2s-1 as in Figure 1. Intervals of light excitation are indicated by shaded regions. (Panels 1-4) The light–induced pmf (1, 2A-D) of ATP synthases with c–stoichiometries of 12 (green, column A), 13 (orange, column B), 14 (black, column C), or 15 (cyan, column D)are shown in units of volts, so that a ΔpH of one is equivalent to 0.06 V. The total pmf (panel 2), Δψ (panel 3), and ΔpH (panel 4) are shown as light–induced changes relative to the pmf dark values indicated as ΔV from dark values, to emphasize light–induced ATP synthase constraints. (5) Light–induced changes in lumen pH due to photosynthetic activity. Light intensities and c–ring composition as in (1). (6) The relative rate constant for plastoquinol oxidation at the cytochrome b6f complex and (7) the extent of nonphotochemical quenching qE component for each c–ring size due to the light–induced changes in lumen pH.

In [25]:

```
#generate a one hour square light pulse
baseline_duration=150 #in seconds
baseline_intensity=0 #dark baseline
pulse_duration=3600 #3600 seconds pulse
pulse_intensity=201.3 #pulse is 201.3 units
recovery_duration = 500 #500 seconds recovery
recovery_intensity=0 #recovery is dark
rise_time=1 #100 ms for the light to rise
time_units='seconds'
point_frequency=100 #start with a frequency of 100 points per su
btrace
repeat_cycles=1 #do this once
wave=generate_square_wave_based_light_sequence (baseline_duratio
n, baseline_intensity,
                    pulse_duration, pulse_intensity, recovery_du
ration, recovery_intensity,
                    rise_time, time_units, point_frequency, repe
at_cycles)
light_pattern['single_square_60_min_201.3_max']=wave
```

In [26]:

```
#run simulations for the one hour pulse
#all standard conditions remain unchanged
#vary n (H+/ATP) based on each c-subunit stoichiometry in set_of
_c_stoichiometries dictionary

for c_stoichiometry in set_of_c_stoichiometries:
    n = c_stoichiometry/3
    on='single pulse 201.3umolE subunits='+str(c_stoichiometry)
#the output name

    Kx=sim_constants() #generrate arrays contining optimized tim
e segments for the simulation
    Kx.k_KEA=0
    Kx.fraction_pH_effect=.25

    Kx.n=n
    constants_dict[on]=Kx #store constants in constants_dict

    constants_dict[on]=Kx #store constants in constants_dict

    output_dict[on], starting_conditions_dict[on]=sim(Kx, origin
al_dark_equilibrated_initial_y,
                                            light_pattern['singl
e_square_60_min_201.3_max'],
                                            max_light_change, po
ints_per_segment, dark_equilibration=60*60)

    Changed_Constants_Table('Change Constants', Kx_initial, Kx)
```

Change Constants

Changed Parameter	Old Value	New Value
n	4.666666666666667	2.6666666666666665

Change Constants

Changed Parameter	Old Value	New Value
n	4.666666666666667	3.6666666666666665

Change Constants

Changed Parameter	Old Value	New Value
n	4.666666666666667	4.0

Change Constants

Changed Parameter	Old Value	New Value
n	4.666666666666667	4.333333333333333

Change Constants

Changed Parameter	Old Value	New Value

Change Constants

Changed Parameter	Old Value	New Value
n	4.666666666666667	5.0

Change Constants

Changed Parameter	Old Value	New Value
n	4.666666666666667	5.666666666666667

In [27]:

```
#make a single one hour sin wave with max PAR of 402.71 umolE

total_duration=60*60 #duration is in seconds, so we do 3600
light_frequency=1/(60*60) #make the frequency the same as the du
ration to get one cycle
points_per_second=10
max_PAR=402.71
wave=generate_sin_wave (total_duration, max_PAR, light_frequency
, points_per_second)
light_pattern['single_sin_wave_1_hr_402.71_max']=wave
```

In [28]:

```
#run simulations for the one hour sin wave
#all standard conditions remain unchanged
#vary n (H+/ATP) based on each c-subunit stoichiometry in set_of
_c_stoichiometries dictionary

for c_stoichiometry in set_of_c_stoichiometries:
    n = c_stoichiometry/3
    on='single sin wave 402.71umolE subunits='+str(c_stoichiomet
ry) #the output name

    Kx=sim_constants() #generrate arrays contining optimized tim
e segments for the simulation
    Kx.k_KEA=0
    Kx.fraction_pH_effect=.25

    Kx.n=n
    constants_dict[on]=Kx #store constants in constants_dict

    constants_dict[on]=Kx #store constants in constants_dict

    output_dict[on], starting_conditions_dict[on]=sim(Kx, origin
al_dark_equilibrated_initial_y,
                                                light_pattern['singl
e_sin_wave_1_hr_402.71_max'],
                                                max_light_change, po
ints_per_segment, dark_equilibration=60*60)

    Changed_Constants_Table('Change Constants', Kx_initial, Kx)
```

Change Constants

Changed Parameter	Old Value	New Value
light_per_L	0.0	0.9991346263789401
n	4.666666666666667	2.6666666666666665

Change Constants

Changed Parameter	Old Value	New Value
light_per_L	0.0	0.9991346263789401
n	4.666666666666667	3.6666666666666665

Change Constants

Changed Parameter	Old Value	New Value
light_per_L	0.0	0.9991346263789401
n	4.666666666666667	4.0

Change Constants

Changed Parameter	Old Value	New Value
light_per_L	0.0	0.9991346263789401
n	4.666666666666667	4.333333333333333

Change Constants

Changed Parameter	Old Value	New Value
light_per_L	0.0	0.9991346263789401

Change Constants

Changed Parameter	Old Value	New Value
light_per_L	0.0	0.9991346263789401
n	4.666666666666667	5.0

Change Constants

Changed Parameter	Old Value	New Value
light_per_L	0.0	0.9991346263789401
n	4.666666666666667	5.666666666666667

In [29]:

```
#run simulations for a one hour square light wave
#5 minute light cycle between 0 umolE and 400 umolE
#all standard conditions remain unchanged
#vary n (H+/ATP) based on each c-subunit stoichiometry in set_of
_c_stoichiometries dictionary

for c_stoichiometry in set_of_c_stoichiometries:
    n = c_stoichiometry/3
    on='square wave 60 min max 400 light subunits='+str(c_stoich
iometry) #the output name

    Kx=sim_constants() #generate arrays contining optimized time
segments for the simulation
    Kx.k_KEA=0
    Kx.fraction_pH_effect=.25

    Kx.n=n
    constants_dict[on]=Kx #store constants in constants_dict

    constants_dict[on]=Kx #store constants in constants_dict

    output_dict[on], starting_conditions_dict[on]=sim(Kx, origin
al_dark_equilibrated_initial_y,
                                            light_pattern['one_h
our_5_min_cycle_square_wave_max_PAR_600'],
                                            max_light_change, po
ints_per_segment, dark_equilibration=60*60)
    Changed_Constants_Table('Change Constants', Kx_initial, Kx)
```

Change Constants

Changed Parameter	Old Value	New Value
n	4.666666666666667	2.6666666666666665

Change Constants

Changed Parameter	Old Value	New Value
n	4.666666666666667	3.6666666666666665

Change Constants

Changed Parameter	Old Value	New Value
n	4.666666666666667	4.0

Change Constants

Changed Parameter	Old Value	New Value
n	4.666666666666667	4.333333333333333

Change Constants

Changed Parameter	Old Value	New Value

Change Constants

Changed Parameter	Old Value	New Value
n	4.666666666666667	5.0

Change Constants

Changed Parameter	Old Value	New Value
n	4.666666666666667	5.666666666666667

In [59]:

```
print('Supplemental Figure 2:  Altered ATP synthase c8 stoichiom
etry impacts pmf composition and pH—mediated '
      'regulatory processes during photosynthesis under increasi
ngly dynamic light environments.')
fig = plt.figure('Supplemental Figure 2', figsize=(5,4), dpi=200
)
ax1 = fig.add_subplot(8,3,1)
ax2 = fig.add_subplot(8,3,2)
ax3 = fig.add_subplot(8,3,3)
ax4 = fig.add_subplot(8,3,4)
ax5 = fig.add_subplot(8,3,5)
ax6 = fig.add_subplot(8,3,6)
ax7 = fig.add_subplot(8,3,7)
ax8 = fig.add_subplot(8,3,8)
ax9 = fig.add_subplot(8,3,9)
ax10 = fig.add_subplot(8,3,10)
ax11 = fig.add_subplot(8,3,11)
ax12 = fig.add_subplot(8,3,12)
ax13 = fig.add_subplot(8,3,13)
ax14 = fig.add_subplot(8,3,14)
ax15 = fig.add_subplot(8,3,15)
ax16 = fig.add_subplot(8,3,16)
ax17 = fig.add_subplot(8,3,17)
ax18 = fig.add_subplot(8,3,18)
ax19 = fig.add_subplot(8,3,19)
ax20 = fig.add_subplot(8,3,20)
ax21 = fig.add_subplot(8,3,21)

ax1b = ax1.twinx()
ax2b = ax2.twinx()
ax3b = ax3.twinx()
ax4b = ax4.twinx()
ax5b = ax5.twinx()
ax6b = ax6.twinx()
ax7b = ax7.twinx()
ax8b = ax8.twinx()
ax9b = ax9.twinx()
ax10b = ax10.twinx()
```

```
ax11b = ax11.twinx()
ax12b = ax12.twinx()
ax13b = ax13.twinx()
ax14b = ax14.twinx()
ax15b = ax15.twinx()
ax16b = ax16.twinx()
ax17b = ax17.twinx()
ax18b = ax18.twinx()
ax19b = ax19.twinx()
ax20b = ax20.twinx()
ax21b = ax21.twinx()

for key in output_dict:
    c=key.split('=')[1]
    if c == "8":
        if "single pulse 201.3umolE subunits" in str(key):
            ax1.plot(output_dict[key]['time_axis']/60,output_dic
t[key]['pmf'],
                    color=set_of_c_stoichiometries[int(c)],linew
idth=1)
            ax4.plot(output_dict[key]['time_axis']/60,output_dic
t[key]['pmf_offset'],
                    color=set_of_c_stoichiometries[int(c)],linew
idth=1)
            ax7.plot(output_dict[key]['time_axis']/60,output_dic
t[key]['Dy_offset'],
                    color=set_of_c_stoichiometries[int(c)],linew
idth=1)
            ax10.plot(output_dict[key]['time_axis']/60,output_di
ct[key]['delta_pH_V_offset'],
                    color=set_of_c_stoichiometries[int(c)],linew
idth=1)
            ax13.plot(output_dict[key]['time_axis']/60,output_di
ct[key]['pHlumen'],
                    color=set_of_c_stoichiometries[int(c)],linew
idth=1)
            ax16.plot(output_dict[key]['time_axis']/60,output_di
ct[key]['b6f_control'],
                    color=set_of_c_stoichiometries[int(c)],linew
idth=1)
            ax19.plot(output_dict[key]['time_axis']/60,output_di
ct[key]['NPQ_array'],
                    color=set_of_c_stoichiometries[int(c)],linew
idth=1)
```

```
            ax1b.fill_between(output_dict[key]['time_axis']/60,
output_dict[key]['light_curve'],0,
                                  color='red', alpha=0.1,zorder=2)
            ax4b.fill_between(output_dict[key]['time_axis']/60,
output_dict[key]['light_curve'],0,
                                  color='red', alpha=0.1,zorder=2)
            ax7b.fill_between(output_dict[key]['time_axis']/60,
output_dict[key]['light_curve'],0,
                                  color='red', alpha=0.1,zorder=2)
            ax10b.fill_between(output_dict[key]['time_axis']/60,
output_dict[key]['light_curve'],0,
                                   color='red', alpha=0.1,zorder=2)
            ax13b.fill_between(output_dict[key]['time_axis']/60,
output_dict[key]['light_curve'],0,
                                   color='red', alpha=0.1,zorder=2)
            ax16b.fill_between(output_dict[key]['time_axis']/60,
output_dict[key]['light_curve'],0,
                                   color='red', alpha=0.1,zorder=2)
            ax19b.fill_between(output_dict[key]['time_axis']/60,
output_dict[key]['light_curve'],0,
                                   color='red', alpha=0.1,zorder=2)
        if "single sin wave 402.71umolE subunits" in str(key):
            ax2.plot(output_dict[key]['time_axis']/60,output_dic
t[key]['pmf'],
                     color=set_of_c_stoichiometries[int(c)],line
width=1)
            ax5.plot(output_dict[key]['time_axis']/60,output_dic
t[key]['pmf_offset'],
                     color=set_of_c_stoichiometries[int(c)],line
width=1)
            ax8.plot(output_dict[key]['time_axis']/60,output_dic
t[key]['Dy_offset'],
                     color=set_of_c_stoichiometries[int(c)],line
width=1)
            ax11.plot(output_dict[key]['time_axis']/60,output_di
ct[key]['delta_pH_V_offset'],
                      color=set_of_c_stoichiometries[int(c)],lin
ewidth=1)
            ax14.plot(output_dict[key]['time_axis']/60,output_di
ct[key]['pHlumen'],
                      color=set_of_c_stoichiometries[int(c)],lin
ewidth=1)
            ax17.plot(output_dict[key]['time_axis']/60,output_di
ct[key]['b6f_control'],
```

```
                    color=set_of_c_stoichiometries[int(c)],lin
ewidth=1)
            ax20.plot(output_dict[key]['time_axis']/60,output_di
ct[key]['NPQ_array'],
                    color=set_of_c_stoichiometries[int(c)],lin
ewidth=1)


            ax2b.fill_between(output_dict[key]['time_axis']/60,
output_dict[key]['light_curve'],0,
                            color='red', alpha=0.1,zorder=2)
            ax5b.fill_between(output_dict[key]['time_axis']/60,
output_dict[key]['light_curve'],0,
                            color='red', alpha=0.1,zorder=2)
            ax8b.fill_between(output_dict[key]['time_axis']/60,
output_dict[key]['light_curve'],0,
                            color='red', alpha=0.1,zorder=2)
            ax11b.fill_between(output_dict[key]['time_axis']/60,
output_dict[key]['light_curve'],0,
                             color='red', alpha=0.1,zorder=2)
            ax14b.fill_between(output_dict[key]['time_axis']/60,
output_dict[key]['light_curve'],0,
                             color='red', alpha=0.1,zorder=2)
            ax17b.fill_between(output_dict[key]['time_axis']/60,
output_dict[key]['light_curve'],0,
                             color='red', alpha=0.1,zorder=2)
            ax20b.fill_between(output_dict[key]['time_axis']/60,
output_dict[key]['light_curve'],0,
                             color='red', alpha=0.1,zorder=2)

        if "square wave 60 min max 400 light subunits" in str(ke
y):
            ax3.plot(output_dict[key]['time_axis']/60,output_dic
t[key]['pmf'],
                     color=set_of_c_stoichiometries[int(c)],line
width=1)
            ax6.plot(output_dict[key]['time_axis']/60,output_dic
t[key]['pmf_offset'],
                     color=set_of_c_stoichiometries[int(c)],line
width=1)
            ax9.plot(output_dict[key]['time_axis']/60,output_dic
t[key]['Dy_offset'],
                     color=set_of_c_stoichiometries[int(c)],line
width=1)
```

```
            ax12.plot(output_dict[key]['time_axis']/60,output_di
ct[key]['delta_pH_V_offset'],
                      color=set_of_c_stoichiometries[int(c)],lin
ewidth=1)
            ax15.plot(output_dict[key]['time_axis']/60,output_di
ct[key]['pHlumen'],
                      color=set_of_c_stoichiometries[int(c)],lin
ewidth=1)
            ax18.plot(output_dict[key]['time_axis']/60,output_di
ct[key]['b6f_control'],
                      color=set_of_c_stoichiometries[int(c)],lin
ewidth=1)
            ax21.plot(output_dict[key]['time_axis']/60,output_di
ct[key]['NPQ_array'],
                      color=set_of_c_stoichiometries[int(c)],lin
ewidth=1)


            ax3b.fill_between(output_dict[key]['time_axis']/60,
output_dict[key]['light_curve'],0,
                              color='red', alpha=0.1,zorder=2)
            ax6b.fill_between(output_dict[key]['time_axis']/60,
output_dict[key]['light_curve'],0,
                              color='red', alpha=0.1,zorder=2)
            ax9b.fill_between(output_dict[key]['time_axis']/60,
output_dict[key]['light_curve'],0,
                              color='red', alpha=0.1,zorder=2)
            ax12b.fill_between(output_dict[key]['time_axis']/60,
output_dict[key]['light_curve'],0,
                               color='red', alpha=0.1,zorder=2)
            ax15b.fill_between(output_dict[key]['time_axis']/60,
output_dict[key]['light_curve'],0,
                               color='red', alpha=0.1,zorder=2)
            ax18b.fill_between(output_dict[key]['time_axis']/60,
output_dict[key]['light_curve'],0,
                               color='red', alpha=0.11,zorder=2)
            ax21b.fill_between(output_dict[key]['time_axis']/60,
output_dict[key]['light_curve'],0,
                               color='red', alpha=0.1,zorder=2)
    if c == "14":
        if "single pulse 201.3umolE subunits" in str(key):
            ax1.plot(output_dict[key]['time_axis']/60,output_dic
t[key]['pmf'],
                     color=set_of_c_stoichiometries[int(c)],line
```

```
width=1)

            ax4.plot(output_dict[key]['time_axis']/60,output_dic
t[key]['pmf_offset'],
                     color=set_of_c_stoichiometries[int(c)],line
width=1)
            ax7.plot(output_dict[key]['time_axis']/60,output_dic
t[key]['Dy_offset'],
                     color=set_of_c_stoichiometries[int(c)],line
width=1)
            ax10.plot(output_dict[key]['time_axis']/60,output_di
ct[key]['delta_pH_V_offset'],
                      color=set_of_c_stoichiometries[int(c)],lin
ewidth=1)
            ax13.plot(output_dict[key]['time_axis']/60,output_di
ct[key]['pHlumen'],
                      color=set_of_c_stoichiometries[int(c)],lin
ewidth=1)
            ax16.plot(output_dict[key]['time_axis']/60,output_di
ct[key]['b6f_control'],
                      color=set_of_c_stoichiometries[int(c)],lin
ewidth=1)
            ax19.plot(output_dict[key]['time_axis']/60,output_di
ct[key]['NPQ_array'],
                      color=set_of_c_stoichiometries[int(c)],lin
ewidth=1)
        if "single sin wave 402.71umolE subunits" in str(key):
            ax2.plot(output_dict[key]['time_axis']/60,output_dic
t[key]['pmf'],
                     color=set_of_c_stoichiometries[int(c)],line
width=1)
            ax5.plot(output_dict[key]['time_axis']/60,output_dic
t[key]['pmf_offset'],
                     color=set_of_c_stoichiometries[int(c)],line
width=1)
            ax8.plot(output_dict[key]['time_axis']/60,output_dic
t[key]['Dy_offset'],
                     color=set_of_c_stoichiometries[int(c)],line
width=1)
            ax11.plot(output_dict[key]['time_axis']/60,output_di
ct[key]['delta_pH_V_offset'],
                      color=set_of_c_stoichiometries[int(c)],lin
ewidth=1)
            ax14.plot(output_dict[key]['time_axis']/60,output_di
ct[key]['pHlumen'],
```

```
                        color=set_of_c_stoichiometries[int(c)],lin
ewidth=1)
            ax17.plot(output_dict[key]['time_axis']/60,output_di
ct[key]['b6f_control'],
                        color=set_of_c_stoichiometries[int(c)],lin
ewidth=1)
            ax20.plot(output_dict[key]['time_axis']/60,output_di
ct[key]['NPQ_array'],
                        color=set_of_c_stoichiometries[int(c)],lin
ewidth=1)
        if "square wave 60 min max 400 light subunits" in str(ke
y):
            ax3.plot(output_dict[key]['time_axis']/60,output_dic
t[key]['pmf'],
                       color=set_of_c_stoichiometries[int(c)],line
width=1)
            ax6.plot(output_dict[key]['time_axis']/60,output_dic
t[key]['pmf_offset'],
                       color=set_of_c_stoichiometries[int(c)],line
width=1)
            ax9.plot(output_dict[key]['time_axis']/60,output_dic
t[key]['Dy_offset'],
                       color=set_of_c_stoichiometries[int(c)],line
width=1)
            ax12.plot(output_dict[key]['time_axis']/60,output_di
ct[key]['delta_pH_V_offset'],
                        color=set_of_c_stoichiometries[int(c)],lin
ewidth=1)
            ax15.plot(output_dict[key]['time_axis']/60,output_di
ct[key]['pHlumen'],
                        color=set_of_c_stoichiometries[int(c)],lin
ewidth=1)
            ax18.plot(output_dict[key]['time_axis']/60,output_di
ct[key]['b6f_control'],
                        color=set_of_c_stoichiometries[int(c)],lin
ewidth=1)
            ax21.plot(output_dict[key]['time_axis']/60,output_di
ct[key]['NPQ_array'],
                        color=set_of_c_stoichiometries[int(c)],lin
ewidth=1)


ax1.set_ylabel('pmf (V)',fontsize=7)
ax4.set_ylabel('pmf\n ($\Delta$V)',fontsize=7)
```

```
ax7.set_ylabel('$\Delta$$\psi$\n ($\Delta$V)',fontsize=7)

ax10.set_ylabel('$\Delta$pH\n ($\Delta$V)',fontsize=7)
ax13.set_ylabel('Lumen pH',fontsize=7)
ax16.set_ylabel('b$_6$f Rate\n (s$^-$$^1$)',fontsize=7)
ax19.set_ylabel('NPQ',fontsize=7)
ax3b.set_ylabel('Intensity',fontsize=7)
ax6b.set_ylabel('Intensity',fontsize=7)
ax9b.set_ylabel('Intensity',fontsize=7)
ax12b.set_ylabel('Intensity',fontsize=7)
ax15b.set_ylabel('Intensity',fontsize=7)
ax18b.set_ylabel('Intensity',fontsize=7)
ax21b.set_ylabel('Intensity',fontsize=7)

axes = [ax1,ax2,ax3,ax4,ax5,ax6,ax7,ax8,ax9,ax10,ax11,ax12,ax13,
ax14,ax15,ax16,ax17,ax18,
        ax1b,ax2b,ax3b,ax4b,ax5b,ax6b,ax7b,ax8b,ax9b,ax10b,ax11b
,ax12b,ax13b,ax14b,ax15b,
        ax16b,ax17b,ax18b,ax19b,ax20b,ax21b]
for i in range(len(axes)):
    subplot=axes[i]
    subplot.set_xticklabels([])

axes = [ax2,ax3,ax5,ax6,ax8,ax9,ax11,ax12,ax14,ax15,ax17,ax18,ax
20,ax21,
        ax1b,ax2b,ax4b,ax5b,ax7b,ax8b,ax10b,ax11b,ax13b,ax14b,ax
16b,ax17b,ax19b,ax20b]
for i in range(len(axes)):
    subplot=axes[i]
    subplot.set_yticklabels([])

props = dict(boxstyle='circle', facecolor='white')
labels = ['A1','B1','C1']
axes = [ax1,ax2,ax3]
for i in range(len(axes)):
    subplot=axes[i]
    label=labels[i]
    subplot.text(-2, .45, label, fontsize=6,verticalalignment='t
op', bbox=props)
    subplot.set_ylim(bottom=0, top=0.28)
axes = [ax4,ax5,ax6]
labels = ['A2','B2','C2']
for i in range(len(axes)):
    subplot=axes[i]
    label=labels[i]
```

```
    subplot.text(-2, .25, label, fontsize=6,verticalalignment='t

op', bbox=props)
    subplot.set_ylim(bottom=-0.02, top=0.15)
axes = [ax7,ax8,ax9]
labels = ['A3','B3','C3']
for i in range(len(axes)):
    subplot=axes[i]
    label=labels[i]
    subplot.text(-2, .25, label, fontsize=6,verticalalignment='t
op', bbox=props)
    subplot.set_ylim(bottom=-0.05, top=0.15)
axes = [ax10,ax11,ax12]
labels = ['A4','B4','C4']
for i in range(len(axes)):
    subplot=axes[i]
    label=labels[i]
    subplot.text(-2, .08, label, fontsize=6,verticalalignment='t
op', bbox=props)
    subplot.set_ylim(bottom=-0.01, top=0.05)
axes = [ax13,ax14,ax15]
labels = ['A5','B5','C5']
for i in range(len(axes)):
    subplot=axes[i]
    label=labels[i]
    subplot.text(-2, 7.8, label, fontsize=6,verticalalignment='t
op', bbox=props)
    subplot.set_ylim(bottom=5.5, top=7)
axes = [ax16,ax17,ax18]
labels = ['A6','B6','C6']
for i in range(len(axes)):
    subplot=axes[i]
    label=labels[i]
    subplot.text(-2, 550, label, fontsize=6,verticalalignment='t
op', bbox=props)
    subplot.set_ylim(bottom=0, top=350)
axes = [ax19,ax20,ax21]
labels = ['A7','B7','C7']
for i in range(len(axes)):
    subplot=axes[i]
    label=labels[i]
    subplot.text(-2, 7.5, label, fontsize=6,verticalalignment='t
op', bbox=props)
    subplot.set_ylim(bottom=0, top=5)
    subplot.set_xlabel('Time (min)',fontsize=7)
```

```
[illegible]


axes = [ax1,ax4,ax7,ax10,ax13,ax16,ax19]
for i in range(len(axes)):
    subplot=axes[i]
    subplot.tick_params(axis='y',labelsize='small')

axes = [ax1b,ax2b,ax3b,ax4b,ax5b,ax6b,ax7b,
        ax8b,ax9b,ax10b,ax11b,ax12b,ax13b,ax14b,
        ax15b,ax16b,ax17b,ax18b,ax19b,ax20b,ax21b]
for i in range(len(axes)):
    subplot = axes[i]
    subplot.set_ylim(bottom=0, top=425)
    subplot.yaxis.label.set_color('red')
    subplot.spines['right'].set_color('red')
    subplot.tick_params(axis='y', colors='red')
    if i not in [2,5,8,11,14,17,20]:
        subplot.set_yticklabels([])

plt.tight_layout(pad=1, w_pad=0.5, h_pad=0)
plt.show()
print('Simulated responses of the light reactions were performed
as in Davis et al., 2017, with all standard '
      'conditions held constant except for the number of ATP syn
thase c-subunits. Simulations were performed using '
      '1-hour of either static light (A), sinusoidal light (B),
or square wave fluctuating light (C) with equal '
      'total photon flux over the total duration of each light t
reatment. Intervals of light excitation are '
      'indicated by shaded regions. (Panels 1-4) The light–induc
ed pmf of ATP synthases with c–stoichiometries of 8 '
      '(blue) or 14 (black) are shown in units of volts, so that
a ΔpH of one is equivalent to 0.06 V. The total '
      'pmf (panel 2), Δψ (panel 3), and ΔpH (panel 4) are shown
as light–induced changes relative to the pmf dark '
      'values indicated as ΔV from dark values, to emphasize lig
ht–induced ATP synthase constraints. (5) Light–'
      'induced changes in lumen pH due to photosynthetic activit
y. Light intensities and c–ring composition as in '
      '(1). (6) The relative rate constant for plastoquinol oxid
ation at the cytochrome b6f complex and (7) the '
      'extent of nonphotochemical quenching qE component for eac
h c–ring size due to the light–induced changes in '
      'lumen pH.')
```

Supplemental Figure 2: Altered ATP synthase c8 stoichiometry impacts pmf composition and pH–mediated regulatory processes during photosynthesis under increasingly dynamic light environments.

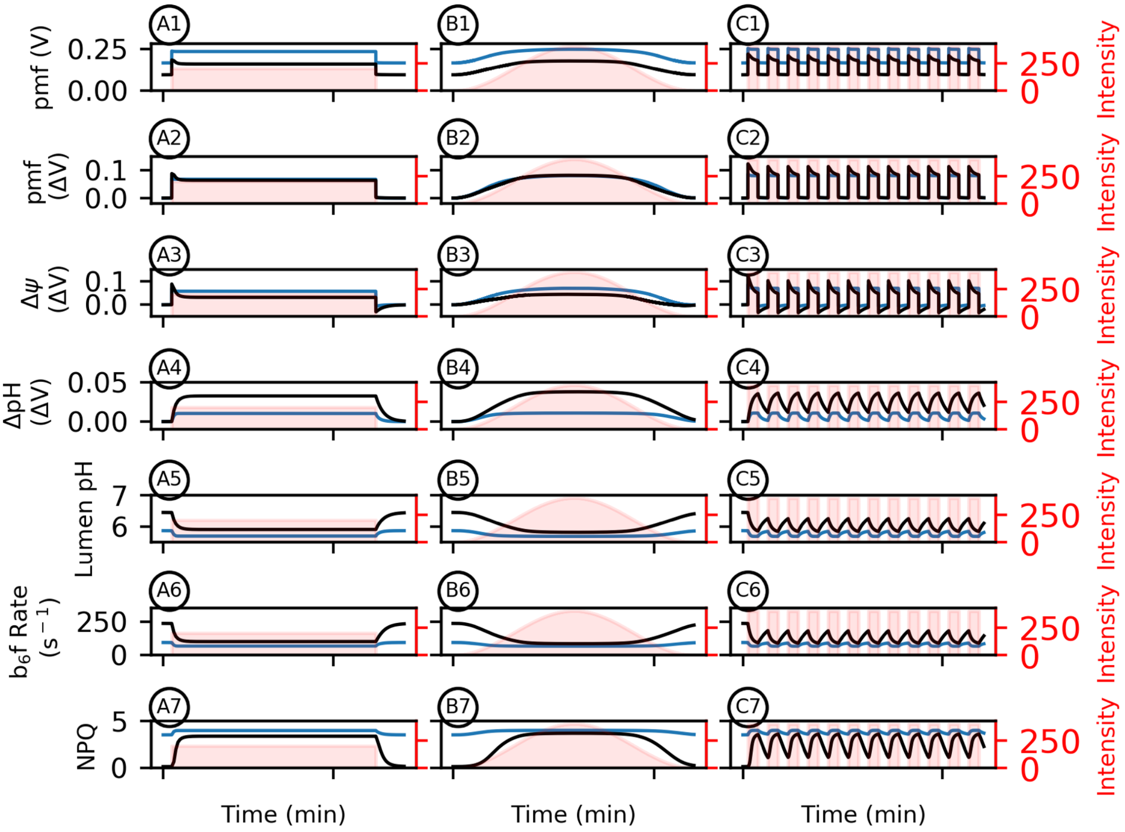

```
Simulated responses of the light reactions were perf
ormed as in Davis et al., 2017, with all standard co
nditions held constant except for the number of ATP
synthase c-subunits. Simulations were performed usin
g 1-hour of either static light (A), sinusoidal ligh
t (B), or square wave fluctuating light (C) with equ
al total photon flux over the total duration of each
light treatment. Intervals of light excitation are i
ndicated by shaded regions. (Panels 1-4) The light–i
nduced pmf of ATP synthases with c–stoichiometries o
f 8 (blue) or 14 (black) are shown in units of volts
, so that a ΔpH of one is equivalent to 0.06 V. The
total pmf (panel 2), Δψ (panel 3), and ΔpH (panel 4)
are shown as light–induced changes relative to the p
mf dark values indicated as ΔV from dark values, to
emphasize light–induced ATP synthase constraints. (5
) Light–induced changes in lumen pH due to photosynt
hetic activity. Light intensities and c–ring composi
tion as in (1). (6) The relative rate constant for p
lastoquinol oxidation at the cytochrome b6f complex
and (7) the extent of nonphotochemical quenching qE
component for each c–ring size due to the light–indu
ced changes in lumen pH.
```

In [60]:

```
print('Supplemental Figure 3:  Altered ATP synthase c11 stoichio
metry impacts pmf composition and pH–mediated '
      'regulatory processes during photosynthesis under increasi
ngly dynamic light environments.')

fig = plt.figure('Supplemental Figure 3', figsize=(5,4), dpi=200
)
ax1 = fig.add_subplot(8,3,1)
ax2 = fig.add_subplot(8,3,2)
ax3 = fig.add_subplot(8,3,3)
ax4 = fig.add_subplot(8,3,4)
ax5 = fig.add_subplot(8,3,5)
ax6 = fig.add_subplot(8,3,6)
ax7 = fig.add_subplot(8,3,7)
ax8 = fig.add_subplot(8,3,8)
ax9 = fig.add_subplot(8,3,9)
ax10 = fig.add_subplot(8,3,10)
ax11 = fig.add_subplot(8,3,11)
```

```
ax11 = fig.add_subplot(8,3,11)
ax12 = fig.add_subplot(8,3,12)

ax13 = fig.add_subplot(8,3,13)
ax14 = fig.add_subplot(8,3,14)
ax15 = fig.add_subplot(8,3,15)
ax16 = fig.add_subplot(8,3,16)
ax17 = fig.add_subplot(8,3,17)
ax18 = fig.add_subplot(8,3,18)
ax19 = fig.add_subplot(8,3,19)
ax20 = fig.add_subplot(8,3,20)
ax21 = fig.add_subplot(8,3,21)

ax1b = ax1.twinx()
ax2b = ax2.twinx()
ax3b = ax3.twinx()
ax4b = ax4.twinx()
ax5b = ax5.twinx()
ax6b = ax6.twinx()
ax7b = ax7.twinx()
ax8b = ax8.twinx()
ax9b = ax9.twinx()
ax10b = ax10.twinx()
ax11b = ax11.twinx()
ax12b = ax12.twinx()
ax13b = ax13.twinx()
ax14b = ax14.twinx()
ax15b = ax15.twinx()
ax16b = ax16.twinx()
ax17b = ax17.twinx()
ax18b = ax18.twinx()
ax19b = ax19.twinx()
ax20b = ax20.twinx()
ax21b = ax21.twinx()

for key in output_dict:
    c=key.split('=')[1]
    if c == "11":
        if "single pulse 201.3umolE subunits" in str(key):
            ax1.plot(output_dict[key]['time_axis']/60,output_dic
t[key]['pmf'],
                     color=set_of_c_stoichiometries[int(c)],line
width=1)
            ax4.plot(output_dict[key]['time_axis']/60,output_dic
t[key]['pmf_offset'],
                     color=set_of_c_stoichiometries[int(c)],line
```

```
width=1)
            ax7.plot(output_dict[key]['time_axis']/60,output_dic
t[key]['Dy_offset'],
                     color=set_of_c_stoichiometries[int(c)],line
width=1)
            ax10.plot(output_dict[key]['time_axis']/60,output_di
ct[key]['delta_pH_V_offset'],
                      color=set_of_c_stoichiometries[int(c)],lin
ewidth=1)
            ax13.plot(output_dict[key]['time_axis']/60,output_di
ct[key]['pHlumen'],
                      color=set_of_c_stoichiometries[int(c)],lin
ewidth=1)
            ax16.plot(output_dict[key]['time_axis']/60,output_di
ct[key]['b6f_control'],
                      color=set_of_c_stoichiometries[int(c)],lin
ewidth=1)
            ax19.plot(output_dict[key]['time_axis']/60,output_di
ct[key]['NPQ_array'],
                      color=set_of_c_stoichiometries[int(c)],lin
ewidth=1)


            ax1b.fill_between(output_dict[key]['time_axis']/60,
output_dict[key]['light_curve'],0,
                              color='red', alpha=0.1,zorder=2)
            ax4b.fill_between(output_dict[key]['time_axis']/60,
output_dict[key]['light_curve'],0,
                              color='red', alpha=0.1,zorder=2)
            ax7b.fill_between(output_dict[key]['time_axis']/60,
output_dict[key]['light_curve'],0,
                              color='red', alpha=0.1,zorder=2)
            ax10b.fill_between(output_dict[key]['time_axis']/60,
output_dict[key]['light_curve'],0,
                               color='red', alpha=0.1,zorder=2)
            ax13b.fill_between(output_dict[key]['time_axis']/60,
output_dict[key]['light_curve'],0,
                               color='red', alpha=0.1,zorder=2)
            ax16b.fill_between(output_dict[key]['time_axis']/60,
output_dict[key]['light_curve'],0,
                               color='red', alpha=0.1,zorder=2)
            ax19b.fill_between(output_dict[key]['time_axis']/60,
output_dict[key]['light_curve'],0,
                               color='red', alpha=0.1,zorder=2)
        if "single sin wave 402.71umolE subunits" in str(key):
```

```
            ax2.plot(output_dict[key]['time_axis']/60,output_dic
t[key]['pmf'],
                     color=set_of_c_stoichiometries[int(c)],line
width=1)
            ax5.plot(output_dict[key]['time_axis']/60,output_dic
t[key]['pmf_offset'],
                     color=set_of_c_stoichiometries[int(c)],line
width=1)
            ax8.plot(output_dict[key]['time_axis']/60,output_dic
t[key]['Dy_offset'],
                     color=set_of_c_stoichiometries[int(c)],line
width=1)
            ax11.plot(output_dict[key]['time_axis']/60,output_di
ct[key]['delta_pH_V_offset'],
                      color=set_of_c_stoichiometries[int(c)],lin
ewidth=1)
            ax14.plot(output_dict[key]['time_axis']/60,output_di
ct[key]['pHlumen'],
                      color=set_of_c_stoichiometries[int(c)],lin
ewidth=1)
            ax17.plot(output_dict[key]['time_axis']/60,output_di
ct[key]['b6f_control'],
                      color=set_of_c_stoichiometries[int(c)],lin
ewidth=1)
            ax20.plot(output_dict[key]['time_axis']/60,output_di
ct[key]['NPQ_array'],
                      color=set_of_c_stoichiometries[int(c)],lin
ewidth=1)



            ax2b.fill_between(output_dict[key]['time_axis']/60,
output_dict[key]['light_curve'],0,
                              color='red', alpha=0.1,zorder=2)
            ax5b.fill_between(output_dict[key]['time_axis']/60,
output_dict[key]['light_curve'],0,
                              color='red', alpha=0.1,zorder=2)
            ax8b.fill_between(output_dict[key]['time_axis']/60,
output_dict[key]['light_curve'],0,
                              color='red', alpha=0.1,zorder=2)
            ax11b.fill_between(output_dict[key]['time_axis']/60,
output_dict[key]['light_curve'],0,
                               color='red', alpha=0.1,zorder=2)
            ax14b.fill_between(output_dict[key]['time_axis']/60,
output_dict[key]['light_curve'],0,
```

```
                                color='red', alpha=0.1,zorder=2)
            ax17b.fill_between(output_dict[key]['time_axis']/60,
output_dict[key]['light_curve'],0,
                                color='red', alpha=0.1,zorder=2)
            ax20b.fill_between(output_dict[key]['time_axis']/60,
output_dict[key]['light_curve'],0,
                                color='red', alpha=0.1,zorder=2)

        if "square wave 60 min max 400 light subunits" in str(ke
y):
            ax3.plot(output_dict[key]['time_axis']/60,output_dic
t[key]['pmf'],
                     color=set_of_c_stoichiometries[int(c)],line
width=1)
            ax6.plot(output_dict[key]['time_axis']/60,output_dic
t[key]['pmf_offset'],
                     color=set_of_c_stoichiometries[int(c)],line
width=1)
            ax9.plot(output_dict[key]['time_axis']/60,output_dic
t[key]['Dy_offset'],
                     color=set_of_c_stoichiometries[int(c)],line
width=1)
            ax12.plot(output_dict[key]['time_axis']/60,output_di
ct[key]['delta_pH_V_offset'],
                      color=set_of_c_stoichiometries[int(c)],lin
ewidth=1)
            ax15.plot(output_dict[key]['time_axis']/60,output_di
ct[key]['pHlumen'],
                      color=set_of_c_stoichiometries[int(c)],lin
ewidth=1)
            ax18.plot(output_dict[key]['time_axis']/60,output_di
ct[key]['b6f_control'],
                      color=set_of_c_stoichiometries[int(c)],lin
ewidth=1)
            ax21.plot(output_dict[key]['time_axis']/60,output_di
ct[key]['NPQ_array'],
                      color=set_of_c_stoichiometries[int(c)],lin
ewidth=1)


            ax3b.fill_between(output_dict[key]['time_axis']/60,
output_dict[key]['light_curve'],0,
                               color='red', alpha=0.1,zorder=2)
            ax6b.fill_between(output_dict[key]['time_axis']/60,
output_dict[key]['light_curve'],0,
```

```
                                    color='red', alpha=0.1,zorder=2)

                ax9b.fill_between(output_dict[key]['time_axis']/60,
output_dict[key]['light_curve'],0,
                                    color='red', alpha=0.1,zorder=2)
                ax12b.fill_between(output_dict[key]['time_axis']/60,
output_dict[key]['light_curve'],0,
                                     color='red', alpha=0.1,zorder=2)
                ax15b.fill_between(output_dict[key]['time_axis']/60,
output_dict[key]['light_curve'],0,
                                     color='red', alpha=0.1,zorder=2)
                ax18b.fill_between(output_dict[key]['time_axis']/60,
output_dict[key]['light_curve'],0,
                                     color='red', alpha=0.11,zorder=2)
                ax21b.fill_between(output_dict[key]['time_axis']/60,
output_dict[key]['light_curve'],0,
                                     color='red', alpha=0.1,zorder=2)
    if c == "14":
        if "single pulse 201.3umolE subunits" in str(key):
            ax1.plot(output_dict[key]['time_axis']/60,output_dic
t[key]['pmf'],
                     color=set_of_c_stoichiometries[int(c)],line
width=1)
            ax4.plot(output_dict[key]['time_axis']/60,output_dic
t[key]['pmf_offset'],
                     color=set_of_c_stoichiometries[int(c)],line
width=1)
            ax7.plot(output_dict[key]['time_axis']/60,output_dic
t[key]['Dy_offset'],
                     color=set_of_c_stoichiometries[int(c)],line
width=1)
            ax10.plot(output_dict[key]['time_axis']/60,output_di
ct[key]['delta_pH_V_offset'],
                      color=set_of_c_stoichiometries[int(c)],lin
ewidth=1)
            ax13.plot(output_dict[key]['time_axis']/60,output_di
ct[key]['pHlumen'],
                      color=set_of_c_stoichiometries[int(c)],lin
ewidth=1)
            ax16.plot(output_dict[key]['time_axis']/60,output_di
ct[key]['b6f_control'],
                      color=set_of_c_stoichiometries[int(c)],lin
ewidth=1)
            ax19.plot(output_dict[key]['time_axis']/60,output_di
ct[key]['NPQ_array'],
```

```
                      color=set_of_c_stoichiometries[int(c)],lin
ewidth=1)
        if "single sin wave 402.71umolE subunits" in str(key):
            ax2.plot(output_dict[key]['time_axis']/60,output_dic
t[key]['pmf'],
                     color=set_of_c_stoichiometries[int(c)],line
width=1)
            ax5.plot(output_dict[key]['time_axis']/60,output_dic
t[key]['pmf_offset'],
                     color=set_of_c_stoichiometries[int(c)],line
width=1)
            ax8.plot(output_dict[key]['time_axis']/60,output_dic
t[key]['Dy_offset'],
                     color=set_of_c_stoichiometries[int(c)],line
width=1)
            ax11.plot(output_dict[key]['time_axis']/60,output_di
ct[key]['delta_pH_V_offset'],
                      color=set_of_c_stoichiometries[int(c)],lin
ewidth=1)
            ax14.plot(output_dict[key]['time_axis']/60,output_di
ct[key]['pHlumen'],
                      color=set_of_c_stoichiometries[int(c)],lin
ewidth=1)
            ax17.plot(output_dict[key]['time_axis']/60,output_di
ct[key]['b6f_control'],
                      color=set_of_c_stoichiometries[int(c)],lin
ewidth=1)
            ax20.plot(output_dict[key]['time_axis']/60,output_di
ct[key]['NPQ_array'],
                      color=set_of_c_stoichiometries[int(c)],lin
ewidth=1)
        if "square wave 60 min max 400 light subunits" in str(ke
y):
            ax3.plot(output_dict[key]['time_axis']/60,output_dic
t[key]['pmf'],
                     color=set_of_c_stoichiometries[int(c)],line
width=1)
            ax6.plot(output_dict[key]['time_axis']/60,output_dic
t[key]['pmf_offset'],
                     color=set_of_c_stoichiometries[int(c)],line
width=1)
            ax9.plot(output_dict[key]['time_axis']/60,output_dic
t[key]['Dy_offset'],
                     color=set_of_c_stoichiometries[int(c)],line
width=1)
```

```
            ax12.plot(output_dict[key]['time_axis']/60,output_di
ct[key]['delta_pH_V_offset'],
                      color=set_of_c_stoichiometries[int(c)],lin
ewidth=1)
            ax15.plot(output_dict[key]['time_axis']/60,output_di
ct[key]['pHlumen'],
                      color=set_of_c_stoichiometries[int(c)],lin
ewidth=1)
            ax18.plot(output_dict[key]['time_axis']/60,output_di
ct[key]['b6f_control'],
                      color=set_of_c_stoichiometries[int(c)],lin
ewidth=1)
            ax21.plot(output_dict[key]['time_axis']/60,output_di
ct[key]['NPQ_array'],
                      color=set_of_c_stoichiometries[int(c)],lin
ewidth=1)

ax1.set_ylabel('pmf (V)',fontsize=7)
ax4.set_ylabel('pmf\n ($\Delta$V)',fontsize=7)
ax7.set_ylabel('$\Delta$$\psi$\n ($\Delta$V)',fontsize=7)
ax10.set_ylabel('$\Delta$pH\n ($\Delta$V)',fontsize=7)
ax13.set_ylabel('Lumen pH',fontsize=7)
ax16.set_ylabel('b$_6$f Rate\n (s$^-$$^1$)',fontsize=7)
ax19.set_ylabel('NPQ',fontsize=7)
ax3b.set_ylabel('Intensity',fontsize=7)
ax6b.set_ylabel('Intensity',fontsize=7)
ax9b.set_ylabel('Intensity',fontsize=7)
ax12b.set_ylabel('Intensity',fontsize=7)
ax15b.set_ylabel('Intensity',fontsize=7)
ax18b.set_ylabel('Intensity',fontsize=7)
ax21b.set_ylabel('Intensity',fontsize=7)

axes = [ax1,ax2,ax3,ax4,ax5,ax6,ax7,ax8,ax9,ax10,ax11,ax12,ax13,
ax14,ax15,ax16,ax17,ax18,
        ax1b,ax2b,ax3b,ax4b,ax5b,ax6b,ax7b,ax8b,ax9b,ax10b,ax11b
,ax12b,ax13b,ax14b,ax15b,ax16b,
        ax17b,ax18b,ax19b,ax20b,ax21b]
for i in range(len(axes)):
    subplot=axes[i]
    subplot.set_xticklabels([])
axes = [ax2,ax3,ax5,ax6,ax8,ax9,ax11,ax12,ax14,ax15,ax17,ax18,ax
20,ax21,
        ax1b,ax2b,ax4b,ax5b,ax7b,ax8b,ax10b,ax11b,ax13b,ax14b,ax
16b,ax17b,ax19b,ax20b]
```

```
for i in range(len(axes)):
    subplot=axes[i]
    subplot.set_yticklabels([])

props = dict(boxstyle='circle', facecolor='white')
labels = ['A1','B1','C1']
axes = [ax1,ax2,ax3]
for i in range(len(axes)):
    subplot=axes[i]
    label=labels[i]
    subplot.text(-2, .45, label, fontsize=6,verticalalignment='t
op', bbox=props)
    subplot.set_ylim(bottom=0, top=0.28)
axes = [ax4,ax5,ax6]
labels = ['A2','B2','C2']
for i in range(len(axes)):
    subplot=axes[i]
    label=labels[i]
    subplot.text(-2, .25, label, fontsize=6,verticalalignment='t
op', bbox=props)
    subplot.set_ylim(bottom=-0.02, top=0.15)
axes = [ax7,ax8,ax9]
labels = ['A3','B3','C3']
for i in range(len(axes)):
    subplot=axes[i]
    label=labels[i]
    subplot.text(-2, .25, label, fontsize=6,verticalalignment='t
op', bbox=props)
    subplot.set_ylim(bottom=-0.05, top=0.15)
axes = [ax10,ax11,ax12]
labels = ['A4','B4','C4']
for i in range(len(axes)):
    subplot=axes[i]
    label=labels[i]
    subplot.text(-2, .08, label, fontsize=6,verticalalignment='t
op', bbox=props)
    subplot.set_ylim(bottom=-0.01, top=0.05)
axes = [ax13,ax14,ax15]
labels = ['A5','B5','C5']
for i in range(len(axes)):
    subplot=axes[i]
    label=labels[i]
    subplot.text(-2, 7.8, label, fontsize=6,verticalalignment='t
op', bbox=props)
    subplot.set_ylim(bottom=5.5, top=7)
```

```
axes = [ax16,ax17,ax18]

labels = ['A6','B6','C6']
for i in range(len(axes)):
    subplot=axes[i]
    label=labels[i]
    subplot.text(-2, 550, label, fontsize=6,verticalalignment='t
op', bbox=props)
    subplot.set_ylim(bottom=0, top=350)
axes = [ax19,ax20,ax21]
labels = ['A7','B7','C7']
for i in range(len(axes)):
    subplot=axes[i]
    label=labels[i]
    subplot.text(-2, 7.5, label, fontsize=6,verticalalignment='t
op', bbox=props)
    subplot.set_ylim(bottom=0, top=5)
    subplot.set_xlabel('Time (min)',fontsize=7)

axes = [ax1,ax4,ax7,ax10,ax13,ax16,ax19]
for i in range(len(axes)):
    subplot=axes[i]
    subplot.tick_params(axis='y',labelsize='small')

axes = [ax1b,ax2b,ax3b,ax4b,ax5b,ax6b,ax7b,
        ax8b,ax9b,ax10b,ax11b,ax12b,ax13b,ax14b,
        ax15b,ax16b,ax17b,ax18b,ax19b,ax20b,ax21b]
for i in range(len(axes)):
    subplot = axes[i]
    subplot.set_ylim(bottom=0, top=425)
    subplot.yaxis.label.set_color('red')
    subplot.spines['right'].set_color('red')
    subplot.tick_params(axis='y', colors='red')
    if i not in [2,5,8,11,14,17,20]:
        subplot.set_yticklabels([])

plt.tight_layout(pad=1, w_pad=0.5, h_pad=0)
plt.show()
print('Simulated responses of the light reactions were performed
as in Davis et al., 2017, with all standard '
      'conditions held constant except for the number of ATP syn
thase c-subunits. Simulations were performed using '
      '1-hour of either static light (A), sinusoidal light (B),
or square wave fluctuating light (C) with equal '
      'total photon flux over the total duration of each light t
```

```
reatment. Intervals of light excitation are '
      'indicated by shaded regions. (Panels 1-4) The light—induc
ed pmf of ATP synthases with c—stoichiometries of '
      '11 (red) or 14 (black) are shown in units of volts, so th
at a ΔpH of one is equivalent to 0.06 V. The total '
      'pmf (panel 2), Δψ (panel 3), and ΔpH (panel 4) are shown
as light—induced changes relative to the pmf dark '
      'values indicated as ΔV from dark values, to emphasize lig
ht—induced ATP synthase constraints. (5) Light—'
      'induced changes in lumen pH due to photosynthetic activit
y. Light intensities and c—ring composition as in '
      '(1). (6) The relative rate constant for plastoquinol oxid
ation at the cytochrome b6f complex and (7) the '
      'extent of nonphotochemical quenching qE component for eac
h c—ring size due to the light—induced changes in '
      'lumen pH.')
```

Supplemental Figure 3: Altered ATP synthase c11 stoichiometry impacts pmf composition and pH—mediated regulatory processes during photosynthesis under increasingly dynamic light environments.

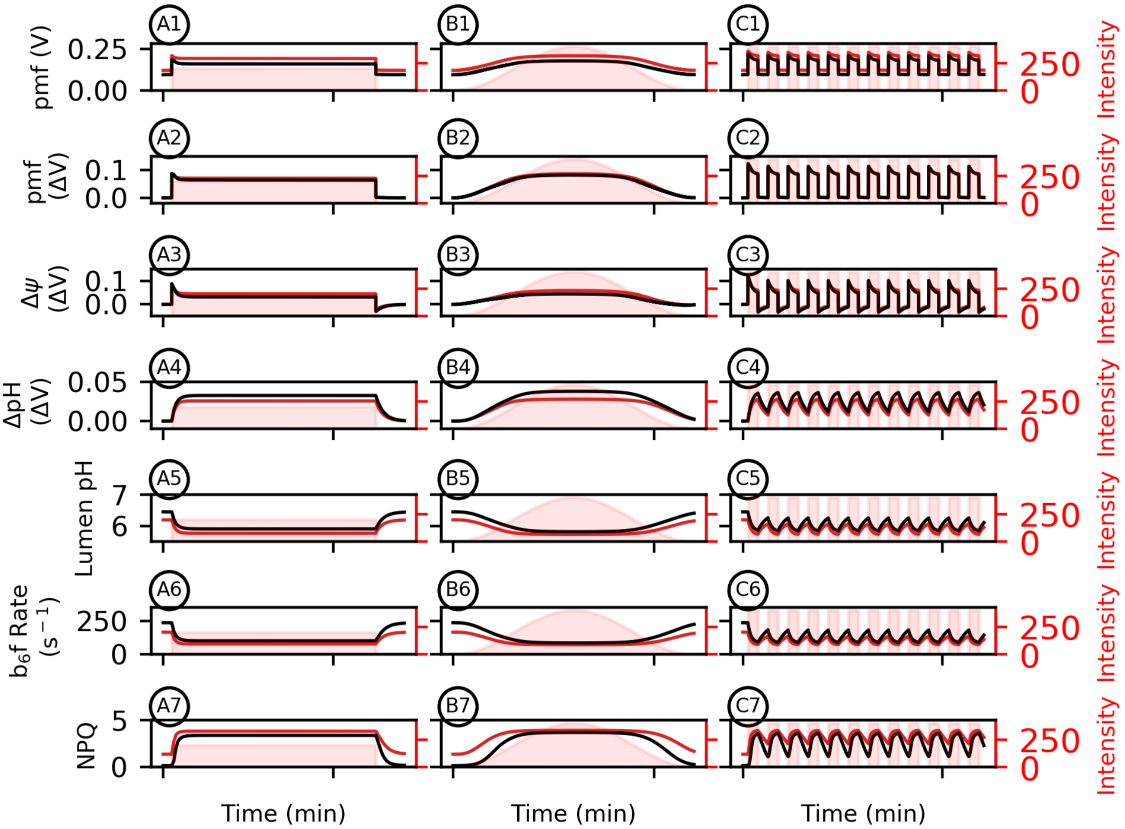

Simulated responses of the light reactions were perf
ormed as in Davis et al., 2017, with all standard co
nditions held constant except for the number of ATP
synthase c-subunits. Simulations were performed usin
g 1-hour of either static light (A), sinusoidal ligh
t (B), or square wave fluctuating light (C) with equ
al total photon flux over the total duration of each
light treatment. Intervals of light excitation are i
ndicated by shaded regions. (Panels 1-4) The light–i
nduced pmf of ATP synthases with c–stoichiometries o
f 11 (red) or 14 (black) are shown in units of volts
, so that a ΔpH of one is equivalent to 0.06 V. The
total pmf (panel 2), Δψ (panel 3), and ΔpH (panel 4)
are shown as light–induced changes relative to the p
mf dark values indicated as ΔV from dark values, to
emphasize light–induced ATP synthase constraints. (5
) Light–induced changes in lumen pH due to photosynt
hetic activity. Light intensities and c–ring composi
tion as in (1). (6) The relative rate constant for p
lastoquinol oxidation at the cytochrome b6f complex
and (7) the extent of nonphotochemical quenching qE
component for each c–ring size due to the light–indu
ced changes in lumen pH.

In [61]:

```
print('Supplemental Figure 4:  Altered ATP synthase c12 stoichio
metry impacts pmf composition and pH–mediated '
      'regulatory processes during photosynthesis under increasi
ngly dynamic light environments.')

fig = plt.figure('Supplemental Figure 4', figsize=(5,4), dpi=200
)
ax1 = fig.add_subplot(8,3,1)
ax2 = fig.add_subplot(8,3,2)
ax3 = fig.add_subplot(8,3,3)
ax4 = fig.add_subplot(8,3,4)
ax5 = fig.add_subplot(8,3,5)
ax6 = fig.add_subplot(8,3,6)
ax7 = fig.add_subplot(8,3,7)
ax8 = fig.add_subplot(8,3,8)
ax9 = fig.add_subplot(8,3,9)
ax10 = fig.add_subplot(8,3,10)
ax11 = fig.add_subplot(8,3,11)
```

```
ax12 = fig.add_subplot(8,3,12)
ax13 = fig.add_subplot(8,3,13)
ax14 = fig.add_subplot(8,3,14)
ax15 = fig.add_subplot(8,3,15)
ax16 = fig.add_subplot(8,3,16)
ax17 = fig.add_subplot(8,3,17)
ax18 = fig.add_subplot(8,3,18)
ax19 = fig.add_subplot(8,3,19)
ax20 = fig.add_subplot(8,3,20)
ax21 = fig.add_subplot(8,3,21)

ax1b = ax1.twinx()
ax2b = ax2.twinx()
ax3b = ax3.twinx()
ax4b = ax4.twinx()
ax5b = ax5.twinx()
ax6b = ax6.twinx()
ax7b = ax7.twinx()
ax8b = ax8.twinx()
ax9b = ax9.twinx()
ax10b = ax10.twinx()
ax11b = ax11.twinx()
ax12b = ax12.twinx()
ax13b = ax13.twinx()
ax14b = ax14.twinx()
ax15b = ax15.twinx()
ax16b = ax16.twinx()
ax17b = ax17.twinx()
ax18b = ax18.twinx()
ax19b = ax19.twinx()
ax20b = ax20.twinx()
ax21b = ax21.twinx()

for key in output_dict:
    c=key.split('=')[1]
    if c == "12":
        if "single pulse 201.3umolE subunits" in str(key):
            ax1.plot(output_dict[key]['time_axis']/60,output_dic
t[key]['pmf'],
                     color=set_of_c_stoichiometries[int(c)],line
width=1)
            ax4.plot(output_dict[key]['time_axis']/60,output_dic
t[key]['pmf_offset'],
                     color=set_of_c_stoichiometries[int(c)],line
width=1)
```

```
            ax7.plot(output_dict[key]['time_axis']/60,output_dic
t[key]['Dy_offset'],
                     color=set_of_c_stoichiometries[int(c)],line
width=1)
            ax10.plot(output_dict[key]['time_axis']/60,output_di
ct[key]['delta_pH_V_offset'],
                      color=set_of_c_stoichiometries[int(c)],lin
ewidth=1)
            ax13.plot(output_dict[key]['time_axis']/60,output_di
ct[key]['pHlumen'],
                      color=set_of_c_stoichiometries[int(c)],lin
ewidth=1)
            ax16.plot(output_dict[key]['time_axis']/60,output_di
ct[key]['b6f_control'],
                      color=set_of_c_stoichiometries[int(c)],lin
ewidth=1)
            ax19.plot(output_dict[key]['time_axis']/60,output_di
ct[key]['NPQ_array'],
                      color=set_of_c_stoichiometries[int(c)],lin
ewidth=1)


            ax1b.fill_between(output_dict[key]['time_axis']/60,
output_dict[key]['light_curve'],0,
                              color='red', alpha=0.1,zorder=2)
            ax4b.fill_between(output_dict[key]['time_axis']/60,
output_dict[key]['light_curve'],0,
                              color='red', alpha=0.1,zorder=2)
            ax7b.fill_between(output_dict[key]['time_axis']/60,
output_dict[key]['light_curve'],0,
                              color='red', alpha=0.1,zorder=2)
            ax10b.fill_between(output_dict[key]['time_axis']/60,
output_dict[key]['light_curve'],0,
                               color='red', alpha=0.1,zorder=2)
            ax13b.fill_between(output_dict[key]['time_axis']/60,
output_dict[key]['light_curve'],0,
                               color='red', alpha=0.1,zorder=2)
            ax16b.fill_between(output_dict[key]['time_axis']/60,
output_dict[key]['light_curve'],0,
                               color='red', alpha=0.1,zorder=2)
            ax19b.fill_between(output_dict[key]['time_axis']/60,
output_dict[key]['light_curve'],0,
                               color='red', alpha=0.1,zorder=2)
        if "single sin wave 402.71umolE subunits" in str(key):
            ax2.plot(output_dict[key]['time_axis']/60,output_dic
```

```
t[key]['pmf'],
                     color=set_of_c_stoichiometries[int(c)],line
width=1)
            ax5.plot(output_dict[key]['time_axis']/60,output_dic
t[key]['pmf_offset'],
                     color=set_of_c_stoichiometries[int(c)],line
width=1)
            ax8.plot(output_dict[key]['time_axis']/60,output_dic
t[key]['Dy_offset'],
                     color=set_of_c_stoichiometries[int(c)],line
width=1)
            ax11.plot(output_dict[key]['time_axis']/60,output_di
ct[key]['delta_pH_V_offset'],
                      color=set_of_c_stoichiometries[int(c)],lin
ewidth=1)
            ax14.plot(output_dict[key]['time_axis']/60,output_di
ct[key]['pHlumen'],
                      color=set_of_c_stoichiometries[int(c)],lin
ewidth=1)
            ax17.plot(output_dict[key]['time_axis']/60,output_di
ct[key]['b6f_control'],
                      color=set_of_c_stoichiometries[int(c)],lin
ewidth=1)
            ax20.plot(output_dict[key]['time_axis']/60,output_di
ct[key]['NPQ_array'],
                      color=set_of_c_stoichiometries[int(c)],lin
ewidth=1)



            ax2b.fill_between(output_dict[key]['time_axis']/60,
output_dict[key]['light_curve'],0,
                              color='red', alpha=0.1,zorder=2)
            ax5b.fill_between(output_dict[key]['time_axis']/60,
output_dict[key]['light_curve'],0,
                              color='red', alpha=0.1,zorder=2)
            ax8b.fill_between(output_dict[key]['time_axis']/60,
output_dict[key]['light_curve'],0,
                              color='red', alpha=0.1,zorder=2)
            ax11b.fill_between(output_dict[key]['time_axis']/60,
output_dict[key]['light_curve'],0,
                               color='red', alpha=0.1,zorder=2)
            ax14b.fill_between(output_dict[key]['time_axis']/60,
output_dict[key]['light_curve'],0,
                               color='red', alpha=0.1,zorder=2)
            ax17b.fill_between(output_dict[key]['time_axis']/60,
```

```
output_dict[key]['light_curve'],0,
                              color='red', alpha=0.1,zorder=2)
            ax20b.fill_between(output_dict[key]['time_axis']/60,
output_dict[key]['light_curve'],0,
                              color='red', alpha=0.1,zorder=2)

        if "square wave 60 min max 400 light subunits" in str(ke
y):
            ax3.plot(output_dict[key]['time_axis']/60,output_dic
t[key]['pmf'],
                     color=set_of_c_stoichiometries[int(c)],line
width=1)
            ax6.plot(output_dict[key]['time_axis']/60,output_dic
t[key]['pmf_offset'],
                     color=set_of_c_stoichiometries[int(c)],line
width=1)
            ax9.plot(output_dict[key]['time_axis']/60,output_dic
t[key]['Dy_offset'],
                     color=set_of_c_stoichiometries[int(c)],line
width=1)
            ax12.plot(output_dict[key]['time_axis']/60,output_di
ct[key]['delta_pH_V_offset'],
                      color=set_of_c_stoichiometries[int(c)],lin
ewidth=1)
            ax15.plot(output_dict[key]['time_axis']/60,output_di
ct[key]['pHlumen'],
                      color=set_of_c_stoichiometries[int(c)],lin
ewidth=1)
            ax18.plot(output_dict[key]['time_axis']/60,output_di
ct[key]['b6f_control'],
                      color=set_of_c_stoichiometries[int(c)],lin
ewidth=1)
            ax21.plot(output_dict[key]['time_axis']/60,output_di
ct[key]['NPQ_array'],
                      color=set_of_c_stoichiometries[int(c)],lin
ewidth=1)


            ax3b.fill_between(output_dict[key]['time_axis']/60,
output_dict[key]['light_curve'],0,
                             color='red', alpha=0.1,zorder=2)
            ax6b.fill_between(output_dict[key]['time_axis']/60,
output_dict[key]['light_curve'],0,
                             color='red', alpha=0.1,zorder=2)
            ax9b.fill_between(output_dict[key]['time_axis']/60,
```

```
output_dict[key]['light_curve'],0,
                              color='red', alpha=0.1,zorder=2)
            ax12b.fill_between(output_dict[key]['time_axis']/60,
output_dict[key]['light_curve'],0,
                               color='red', alpha=0.1,zorder=2)
            ax15b.fill_between(output_dict[key]['time_axis']/60,
output_dict[key]['light_curve'],0,
                               color='red', alpha=0.1,zorder=2)
            ax18b.fill_between(output_dict[key]['time_axis']/60,
output_dict[key]['light_curve'],0,
                               color='red', alpha=0.11,zorder=2)
            ax21b.fill_between(output_dict[key]['time_axis']/60,
output_dict[key]['light_curve'],0,
                               color='red', alpha=0.1,zorder=2)
    if c == "14":
        if "single pulse 201.3umolE subunits" in str(key):
            ax1.plot(output_dict[key]['time_axis']/60,output_dic
t[key]['pmf'],
                     color=set_of_c_stoichiometries[int(c)],line
width=1)
            ax4.plot(output_dict[key]['time_axis']/60,output_dic
t[key]['pmf_offset'],
                     color=set_of_c_stoichiometries[int(c)],line
width=1)
            ax7.plot(output_dict[key]['time_axis']/60,output_dic
t[key]['Dy_offset'],
                     color=set_of_c_stoichiometries[int(c)],line
width=1)
            ax10.plot(output_dict[key]['time_axis']/60,output_di
ct[key]['delta_pH_V_offset'],
                      color=set_of_c_stoichiometries[int(c)],lin
ewidth=1)
            ax13.plot(output_dict[key]['time_axis']/60,output_di
ct[key]['pHlumen'],
                      color=set_of_c_stoichiometries[int(c)],lin
ewidth=1)
            ax16.plot(output_dict[key]['time_axis']/60,output_di
ct[key]['b6f_control'],
                      color=set_of_c_stoichiometries[int(c)],lin
ewidth=1)
            ax19.plot(output_dict[key]['time_axis']/60,output_di
ct[key]['NPQ_array'],
                      color=set_of_c_stoichiometries[int(c)],lin
ewidth=1)
        if "single sin wave 402.71umolE subunits" in str(key):
```

```
            ax2.plot(output_dict[key]['time_axis']/60,output_dic
t[key]['pmf'],
                     color=set_of_c_stoichiometries[int(c)],line
width=1)
            ax5.plot(output_dict[key]['time_axis']/60,output_dic
t[key]['pmf_offset'],
                     color=set_of_c_stoichiometries[int(c)],line
width=1)
            ax8.plot(output_dict[key]['time_axis']/60,output_dic
t[key]['Dy_offset'],
                     color=set_of_c_stoichiometries[int(c)],line
width=1)
            ax11.plot(output_dict[key]['time_axis']/60,output_di
ct[key]['delta_pH_V_offset'],
                      color=set_of_c_stoichiometries[int(c)],lin
ewidth=1)
            ax14.plot(output_dict[key]['time_axis']/60,output_di
ct[key]['pHlumen'],
                      color=set_of_c_stoichiometries[int(c)],lin
ewidth=1)
            ax17.plot(output_dict[key]['time_axis']/60,output_di
ct[key]['b6f_control'],
                      color=set_of_c_stoichiometries[int(c)],lin
ewidth=1)
            ax20.plot(output_dict[key]['time_axis']/60,output_di
ct[key]['NPQ_array'],
                      color=set_of_c_stoichiometries[int(c)],lin
ewidth=1)
        if "square wave 60 min max 400 light subunits" in str(ke
y):
            ax3.plot(output_dict[key]['time_axis']/60,output_dic
t[key]['pmf'],
                     color=set_of_c_stoichiometries[int(c)],line
width=1)
            ax6.plot(output_dict[key]['time_axis']/60,output_dic
t[key]['pmf_offset'],
                     color=set_of_c_stoichiometries[int(c)],line
width=1)
            ax9.plot(output_dict[key]['time_axis']/60,output_dic
t[key]['Dy_offset'],
                     color=set_of_c_stoichiometries[int(c)],line
width=1)
            ax12.plot(output_dict[key]['time_axis']/60,output_di
ct[key]['delta_pH_V_offset'],
                      color=set_of_c_stoichiometries[int(c)],lin
```

```
ewidth=1)
            ax15.plot(output_dict[key]['time_axis']/60,output_di
ct[key]['pHlumen'],
                      color=set_of_c_stoichiometries[int(c)],lin
ewidth=1)
            ax18.plot(output_dict[key]['time_axis']/60,output_di
ct[key]['b6f_control'],
                      color=set_of_c_stoichiometries[int(c)],lin
ewidth=1)
            ax21.plot(output_dict[key]['time_axis']/60,output_di
ct[key]['NPQ_array'],
                      color=set_of_c_stoichiometries[int(c)],lin
ewidth=1)

ax1.set_ylabel('pmf (V)',fontsize=7)
ax4.set_ylabel('pmf\n ($\Delta$V)',fontsize=7)
ax7.set_ylabel('$\Delta$$\psi$\n ($\Delta$V)',fontsize=7)
ax10.set_ylabel('$\Delta$pH\n ($\Delta$V)',fontsize=7)
ax13.set_ylabel('Lumen pH',fontsize=7)
ax16.set_ylabel('b$_6$f Rate\n (s$^-$$^1$)',fontsize=7)
ax19.set_ylabel('NPQ',fontsize=7)
ax3b.set_ylabel('Intensity',fontsize=7)
ax6b.set_ylabel('Intensity',fontsize=7)
ax9b.set_ylabel('Intensity',fontsize=7)
ax12b.set_ylabel('Intensity',fontsize=7)
ax15b.set_ylabel('Intensity',fontsize=7)
ax18b.set_ylabel('Intensity',fontsize=7)
ax21b.set_ylabel('Intensity',fontsize=7)

axes = [ax1,ax2,ax3,ax4,ax5,ax6,ax7,ax8,ax9,ax10,ax11,ax12,ax13,
ax14,ax15,ax16,ax17,ax18,
        ax1b,ax2b,ax3b,ax4b,ax5b,ax6b,ax7b,ax8b,ax9b,ax10b,ax11b
,ax12b,ax13b,ax14b,ax15b,ax16b,
        ax17b,ax18b,ax19b,ax20b,ax21b]
for i in range(len(axes)):
    subplot=axes[i]
    subplot.set_xticklabels([])
axes = [ax2,ax3,ax5,ax6,ax8,ax9,ax11,ax12,ax14,ax15,ax17,ax18,ax
20,ax21,
        ax1b,ax2b,ax4b,ax5b,ax7b,ax8b,ax10b,ax11b,ax13b,ax14b,ax
16b,ax17b,ax19b,ax20b]
for i in range(len(axes)):
    subplot=axes[i]
    subplot.set_yticklabels([])
```

```
props = dict(boxstyle='circle', facecolor='white')
labels = ['A1','B1','C1']
axes = [ax1,ax2,ax3]
for i in range(len(axes)):
    subplot=axes[i]
    label=labels[i]
    subplot.text(-2, .45, label, fontsize=6,verticalalignment='top', bbox=props)
    subplot.set_ylim(bottom=0, top=0.28)
axes = [ax4,ax5,ax6]
labels = ['A2','B2','C2']
for i in range(len(axes)):
    subplot=axes[i]
    label=labels[i]
    subplot.text(-2, .25, label, fontsize=6,verticalalignment='top', bbox=props)
    subplot.set_ylim(bottom=-0.02, top=0.15)
axes = [ax7,ax8,ax9]
labels = ['A3','B3','C3']
for i in range(len(axes)):
    subplot=axes[i]
    label=labels[i]
    subplot.text(-2, .25, label, fontsize=6,verticalalignment='top', bbox=props)
    subplot.set_ylim(bottom=-0.05, top=0.15)
axes = [ax10,ax11,ax12]
labels = ['A4','B4','C4']
for i in range(len(axes)):
    subplot=axes[i]
    label=labels[i]
    subplot.text(-2, .08, label, fontsize=6,verticalalignment='top', bbox=props)
    subplot.set_ylim(bottom=-0.01, top=0.05)
axes = [ax13,ax14,ax15]
labels = ['A5','B5','C5']
for i in range(len(axes)):
    subplot=axes[i]
    label=labels[i]
    subplot.text(-2, 7.8, label, fontsize=6,verticalalignment='top', bbox=props)
    subplot.set_ylim(bottom=5.5, top=7)
axes = [ax16,ax17,ax18]
labels = ['A6','B6','C6']
for i in range(len(axes)):
    subplot=axes[i]
```

```
    label=labels[i]
    subplot.text(-2, 550, label, fontsize=6,verticalalignment='t
op', bbox=props)
    subplot.set_ylim(bottom=0, top=350)
axes = [ax19,ax20,ax21]
labels = ['A7','B7','C7']
for i in range(len(axes)):
    subplot=axes[i]
    label=labels[i]
    subplot.text(-2, 7.5, label, fontsize=6,verticalalignment='t
op', bbox=props)
    subplot.set_ylim(bottom=0, top=5)
    subplot.set_xlabel('Time (min)',fontsize=7)

axes = [ax1,ax4,ax7,ax10,ax13,ax16,ax19]
for i in range(len(axes)):
    subplot=axes[i]
    subplot.tick_params(axis='y',labelsize='small')

axes = [ax1b,ax2b,ax3b,ax4b,ax5b,ax6b,ax7b,
        ax8b,ax9b,ax10b,ax11b,ax12b,ax13b,ax14b,
        ax15b,ax16b,ax17b,ax18b,ax19b,ax20b,ax21b]
for i in range(len(axes)):
    subplot = axes[i]
    subplot.set_ylim(bottom=0, top=425)
    subplot.yaxis.label.set_color('red')
    subplot.spines['right'].set_color('red')
    subplot.tick_params(axis='y', colors='red')
    if i not in [2,5,8,11,14,17,20]:
        subplot.set_yticklabels([])

plt.tight_layout(pad=1, w_pad=0.5, h_pad=0)
plt.show()
print('Simulated responses of the light reactions were performed
as in Davis et al., 2017, with all standard '
      'conditions held constant except for the number of ATP syn
thase c-subunits. Simulations were performed using '
      '1-hour of either static light (A), sinusoidal light (B),
or square wave fluctuating light (C) with equal '
      'total photon flux over the total duration of each light t
reatment. Intervals of light excitation are '
      'indicated by shaded regions. (Panels 1-4) The light—induc
ed pmf of ATP synthases with c—stoichiometries of '
      '12 (green) or 14 (black) are shown in units of volts, so
that a ΔpH of one is equivalent to 0.06 V. The '
```

```
      'total pmf (panel 2), Δψ (panel 3), and ΔpH (panel 4) are shown as light–induced changes relative to the pmf '
      'dark values indicated as ΔV from dark values, to emphasize light–induced ATP synthase constraints. (5) '
      'Light–induced changes in lumen pH due to photosynthetic activity. Light intensities and c–ring composition '
      'as in (1). (6) The relative rate constant for plastoquinol oxidation at the cytochrome b6f complex and (7) '
      'the extent of nonphotochemical quenching qE component for each c–ring size due to the light–induced changes '
      'in lumen pH.')
```

Supplemental Figure 4: Altered ATP synthase c12 stoichiometry impacts pmf composition and pH–mediated regulatory processes during photosynthesis under increasingly dynamic light environments.

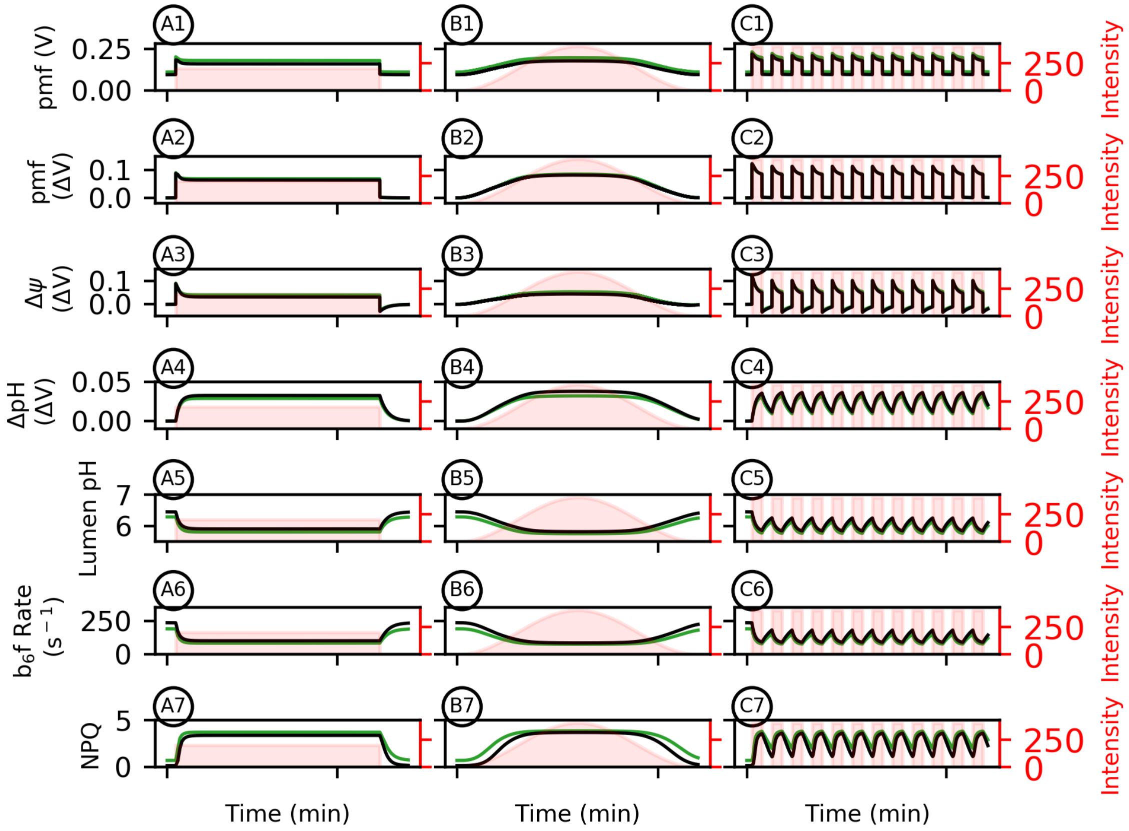

A1
B1
C1
pmf (V)
0.25
0.00
250
0
Intensity
A2
B2
C2
pmf (ΔV)
0.1
0.0
A3
B3
C3
Δψ (ΔV)
A4
B4
C4
ΔpH (ΔV)
0.05
A5
B5
C5
Lumen pH
7
6
A6
B6
C6
b6f Rate (s−1)
A7
B7
C7
NPQ
5
Time (min)

```
Simulated responses of the light reactions were perf
ormed as in Davis et al., 2017, with all standard co
nditions held constant except for the number of ATP
synthase c-subunits. Simulations were performed usin
g 1-hour of either static light (A), sinusoidal ligh
t (B), or square wave fluctuating light (C) with equ
al total photon flux over the total duration of each
light treatment. Intervals of light excitation are i
ndicated by shaded regions. (Panels 1-4) The light–i
nduced pmf of ATP synthases with c–stoichiometries o
f 12 (green) or 14 (black) are shown in units of vol
ts, so that a ΔpH of one is equivalent to 0.06 V. Th
e total pmf (panel 2), Δψ (panel 3), and ΔpH (panel
4) are shown as light–induced changes relative to th
e pmf dark values indicated as ΔV from dark values,
to emphasize light–induced ATP synthase constraints.
(5) Light–induced changes in lumen pH due to photosy
nthetic activity. Light intensities and c–ring compo
sition as in (1). (6) The relative rate constant for
plastoquinol oxidation at the cytochrome b6f complex
and (7) the extent of nonphotochemical quenching qE
component for each c–ring size due to the light–indu
ced changes in lumen pH.
```

In [64]:

```python
print('Supplemental Figure 5:  Altered ATP synthase c13 stoichio
metry impacts pmf composition and pH–mediated '
      'regulatory processes during photosynthesis under increasi
ngly dynamic light environments.')

fig = plt.figure('Supplemental Figure 5', figsize=(5,4), dpi=200
)
ax1 = fig.add_subplot(8,3,1)
ax2 = fig.add_subplot(8,3,2)
ax3 = fig.add_subplot(8,3,3)
ax4 = fig.add_subplot(8,3,4)
ax5 = fig.add_subplot(8,3,5)
ax6 = fig.add_subplot(8,3,6)
ax7 = fig.add_subplot(8,3,7)
ax8 = fig.add_subplot(8,3,8)
ax9 = fig.add_subplot(8,3,9)
ax10 = fig.add_subplot(8,3,10)
ax11 = fig.add_subplot(8,3,11)
[illegible]
```

```
ax12 = fig.add_subplot(8,3,12)
ax13 = fig.add_subplot(8,3,13)

ax14 = fig.add_subplot(8,3,14)
ax15 = fig.add_subplot(8,3,15)
ax16 = fig.add_subplot(8,3,16)
ax17 = fig.add_subplot(8,3,17)
ax18 = fig.add_subplot(8,3,18)
ax19 = fig.add_subplot(8,3,19)
ax20 = fig.add_subplot(8,3,20)
ax21 = fig.add_subplot(8,3,21)

ax1b = ax1.twinx()
ax2b = ax2.twinx()
ax3b = ax3.twinx()
ax4b = ax4.twinx()
ax5b = ax5.twinx()
ax6b = ax6.twinx()
ax7b = ax7.twinx()
ax8b = ax8.twinx()
ax9b = ax9.twinx()
ax10b = ax10.twinx()
ax11b = ax11.twinx()
ax12b = ax12.twinx()
ax13b = ax13.twinx()
ax14b = ax14.twinx()
ax15b = ax15.twinx()
ax16b = ax16.twinx()
ax17b = ax17.twinx()
ax18b = ax18.twinx()
ax19b = ax19.twinx()
ax20b = ax20.twinx()
ax21b = ax21.twinx()

for key in output_dict:
    c=key.split('=')[1]
    if c == "13":
        if "single pulse 201.3umolE subunits" in str(key):
            ax1.plot(output_dict[key]['time_axis']/60,output_dic
t[key]['pmf'],
                     color=set_of_c_stoichiometries[int(c)],line
width=1)
            ax4.plot(output_dict[key]['time_axis']/60,output_dic
t[key]['pmf_offset'],
                     color=set_of_c_stoichiometries[int(c)],line
width=1)
```

```
            ax7.plot(output_dict[key]['time_axis']/60,output_dic
t[key]['Dy_offset'],
                     color=set_of_c_stoichiometries[int(c)],line
width=1)
            ax10.plot(output_dict[key]['time_axis']/60,output_di
ct[key]['delta_pH_V_offset'],
                      color=set_of_c_stoichiometries[int(c)],lin
ewidth=1)
            ax13.plot(output_dict[key]['time_axis']/60,output_di
ct[key]['pHlumen'],
                      color=set_of_c_stoichiometries[int(c)],lin
ewidth=1)
            ax16.plot(output_dict[key]['time_axis']/60,output_di
ct[key]['b6f_control'],
                      color=set_of_c_stoichiometries[int(c)],lin
ewidth=1)
            ax19.plot(output_dict[key]['time_axis']/60,output_di
ct[key]['NPQ_array'],
                      color=set_of_c_stoichiometries[int(c)],lin
ewidth=1)



            ax1b.fill_between(output_dict[key]['time_axis']/60,
output_dict[key]['light_curve'],0,
                              color='red', alpha=0.1,zorder=2)
            ax4b.fill_between(output_dict[key]['time_axis']/60,
output_dict[key]['light_curve'],0,
                              color='red', alpha=0.1,zorder=2)
            ax7b.fill_between(output_dict[key]['time_axis']/60,
output_dict[key]['light_curve'],0,
                              color='red', alpha=0.1,zorder=2)
            ax10b.fill_between(output_dict[key]['time_axis']/60,
output_dict[key]['light_curve'],0,
                               color='red', alpha=0.1,zorder=2)
            ax13b.fill_between(output_dict[key]['time_axis']/60,
output_dict[key]['light_curve'],0,
                               color='red', alpha=0.1,zorder=2)
            ax16b.fill_between(output_dict[key]['time_axis']/60,
output_dict[key]['light_curve'],0,
                               color='red', alpha=0.1,zorder=2)
            ax19b.fill_between(output_dict[key]['time_axis']/60,
output_dict[key]['light_curve'],0,
                               color='red', alpha=0.1,zorder=2)
        if "single sin wave 402.71umolE subunits" in str(key):
            ax2.plot(output_dict[key]['time_axis']/60,output_dic
```

```
[illegible]
t[key]['pmf'],
                     color=set_of_c_stoichiometries[int(c)],line
width=1)
            ax5.plot(output_dict[key]['time_axis']/60,output_dic
t[key]['pmf_offset'],
                     color=set_of_c_stoichiometries[int(c)],line
width=1)
            ax8.plot(output_dict[key]['time_axis']/60,output_dic
t[key]['Dy_offset'],
                     color=set_of_c_stoichiometries[int(c)],line
width=1)
            ax11.plot(output_dict[key]['time_axis']/60,output_di
ct[key]['delta_pH_V_offset'],
                      color=set_of_c_stoichiometries[int(c)],lin
ewidth=1)
            ax14.plot(output_dict[key]['time_axis']/60,output_di
ct[key]['pHlumen'],
                      color=set_of_c_stoichiometries[int(c)],lin
ewidth=1)
            ax17.plot(output_dict[key]['time_axis']/60,output_di
ct[key]['b6f_control'],
                      color=set_of_c_stoichiometries[int(c)],lin
ewidth=1)
            ax20.plot(output_dict[key]['time_axis']/60,output_di
ct[key]['NPQ_array'],
                      color=set_of_c_stoichiometries[int(c)],lin
ewidth=1)



            ax2b.fill_between(output_dict[key]['time_axis']/60,
output_dict[key]['light_curve'],0,
                              color='red', alpha=0.1,zorder=2)
            ax5b.fill_between(output_dict[key]['time_axis']/60,
output_dict[key]['light_curve'],0,
                              color='red', alpha=0.1,zorder=2)
            ax8b.fill_between(output_dict[key]['time_axis']/60,
output_dict[key]['light_curve'],0,
                              color='red', alpha=0.1,zorder=2)
            ax11b.fill_between(output_dict[key]['time_axis']/60,
output_dict[key]['light_curve'],0,
                              color='red', alpha=0.1,zorder=2)
            ax14b.fill_between(output_dict[key]['time_axis']/60,
output_dict[key]['light_curve'],0,
                              color='red', alpha=0.1,zorder=2)
            ax17b.fill_between(output_dict[key]['time_axis']/60,
```

```
output_dict[key]['light_curve'],0,
                              color='red', alpha=0.1,zorder=2)
            ax20b.fill_between(output_dict[key]['time_axis']/60,
output_dict[key]['light_curve'],0,
                              color='red', alpha=0.1,zorder=2)

        if "square wave 60 min max 400 light subunits" in str(ke
y):
            ax3.plot(output_dict[key]['time_axis']/60,output_dic
t[key]['pmf'],
                     color=set_of_c_stoichiometries[int(c)],line
width=1)
            ax6.plot(output_dict[key]['time_axis']/60,output_dic
t[key]['pmf_offset'],
                     color=set_of_c_stoichiometries[int(c)],line
width=1)
            ax9.plot(output_dict[key]['time_axis']/60,output_dic
t[key]['Dy_offset'],
                     color=set_of_c_stoichiometries[int(c)],line
width=1)
            ax12.plot(output_dict[key]['time_axis']/60,output_di
ct[key]['delta_pH_V_offset'],
                      color=set_of_c_stoichiometries[int(c)],lin
ewidth=1)
            ax15.plot(output_dict[key]['time_axis']/60,output_di
ct[key]['pHlumen'],
                      color=set_of_c_stoichiometries[int(c)],lin
ewidth=1)
            ax18.plot(output_dict[key]['time_axis']/60,output_di
ct[key]['b6f_control'],
                      color=set_of_c_stoichiometries[int(c)],lin
ewidth=1)
            ax21.plot(output_dict[key]['time_axis']/60,output_di
ct[key]['NPQ_array'],
                      color=set_of_c_stoichiometries[int(c)],lin
ewidth=1)


            ax3b.fill_between(output_dict[key]['time_axis']/60,
output_dict[key]['light_curve'],0,
                              color='red', alpha=0.1,zorder=2)
            ax6b.fill_between(output_dict[key]['time_axis']/60,
output_dict[key]['light_curve'],0,
                              color='red', alpha=0.1,zorder=2)
            ax9b.fill_between(output_dict[key]['time_axis']/60,
```

```
output_dict[key]['light_curve'],0,
                              color='red', alpha=0.1,zorder=2)
            ax12b.fill_between(output_dict[key]['time_axis']/60,
output_dict[key]['light_curve'],0,
                               color='red', alpha=0.1,zorder=2)
            ax15b.fill_between(output_dict[key]['time_axis']/60,
output_dict[key]['light_curve'],0,
                               color='red', alpha=0.1,zorder=2)
            ax18b.fill_between(output_dict[key]['time_axis']/60,
output_dict[key]['light_curve'],0,
                               color='red', alpha=0.11,zorder=2)
            ax21b.fill_between(output_dict[key]['time_axis']/60,
output_dict[key]['light_curve'],0,
                               color='red', alpha=0.1,zorder=2)
    if c == "14":
        if "single pulse 201.3umolE subunits" in str(key):
            ax1.plot(output_dict[key]['time_axis']/60,output_dic
t[key]['pmf'],
                     color=set_of_c_stoichiometries[int(c)],line
width=1)
            ax4.plot(output_dict[key]['time_axis']/60,output_dic
t[key]['pmf_offset'],
                     color=set_of_c_stoichiometries[int(c)],line
width=1)
            ax7.plot(output_dict[key]['time_axis']/60,output_dic
t[key]['Dy_offset'],
                     color=set_of_c_stoichiometries[int(c)],line
width=1)
            ax10.plot(output_dict[key]['time_axis']/60,output_di
ct[key]['delta_pH_V_offset'],
                      color=set_of_c_stoichiometries[int(c)],lin
ewidth=1)
            ax13.plot(output_dict[key]['time_axis']/60,output_di
ct[key]['pHlumen'],
                      color=set_of_c_stoichiometries[int(c)],lin
ewidth=1)
            ax16.plot(output_dict[key]['time_axis']/60,output_di
ct[key]['b6f_control'],
                      color=set_of_c_stoichiometries[int(c)],lin
ewidth=1)
            ax19.plot(output_dict[key]['time_axis']/60,output_di
ct[key]['NPQ_array'],
                      color=set_of_c_stoichiometries[int(c)],lin
ewidth=1)
        if "single sin wave 402.71umolE subunits" in str(key):
```

```
            ax2.plot(output_dict[key]['time_axis']/60,output_dic
t[key]['pmf'],
                     color=set_of_c_stoichiometries[int(c)],line
width=1)
            ax5.plot(output_dict[key]['time_axis']/60,output_dic
t[key]['pmf_offset'],
                     color=set_of_c_stoichiometries[int(c)],line
width=1)
            ax8.plot(output_dict[key]['time_axis']/60,output_dic
t[key]['Dy_offset'],
                     color=set_of_c_stoichiometries[int(c)],line
width=1)
            ax11.plot(output_dict[key]['time_axis']/60,output_di
ct[key]['delta_pH_V_offset'],
                      color=set_of_c_stoichiometries[int(c)],lin
ewidth=1)
            ax14.plot(output_dict[key]['time_axis']/60,output_di
ct[key]['pHlumen'],
                      color=set_of_c_stoichiometries[int(c)],lin
ewidth=1)
            ax17.plot(output_dict[key]['time_axis']/60,output_di
ct[key]['b6f_control'],
                      color=set_of_c_stoichiometries[int(c)],lin
ewidth=1)
            ax20.plot(output_dict[key]['time_axis']/60,output_di
ct[key]['NPQ_array'],
                      color=set_of_c_stoichiometries[int(c)],lin
ewidth=1)
        if "square wave 60 min max 400 light subunits" in str(ke
y):
            ax3.plot(output_dict[key]['time_axis']/60,output_dic
t[key]['pmf'],
                     color=set_of_c_stoichiometries[int(c)],line
width=1)
            ax6.plot(output_dict[key]['time_axis']/60,output_dic
t[key]['pmf_offset'],
                     color=set_of_c_stoichiometries[int(c)],line
width=1)
            ax9.plot(output_dict[key]['time_axis']/60,output_dic
t[key]['Dy_offset'],
                     color=set_of_c_stoichiometries[int(c)],line
width=1)
            ax12.plot(output_dict[key]['time_axis']/60,output_di
ct[key]['delta_pH_V_offset'],
                      color=set_of_c_stoichiometries[int(c)],lin
```

```
ewidth=1)
            ax15.plot(output_dict[key]['time_axis']/60,output_di
ct[key]['pHlumen'],
                      color=set_of_c_stoichiometries[int(c)],lin
ewidth=1)
            ax18.plot(output_dict[key]['time_axis']/60,output_di
ct[key]['b6f_control'],
                      color=set_of_c_stoichiometries[int(c)],lin
ewidth=1)
            ax21.plot(output_dict[key]['time_axis']/60,output_di
ct[key]['NPQ_array'],
                      color=set_of_c_stoichiometries[int(c)],lin
ewidth=1)

ax1.set_ylabel('pmf (V)',fontsize=7)
ax4.set_ylabel('pmf\n ($\Delta$V)',fontsize=7)
ax7.set_ylabel('$\Delta$$\psi$\n ($\Delta$V)',fontsize=7)
ax10.set_ylabel('$\Delta$pH\n ($\Delta$V)',fontsize=7)
ax13.set_ylabel('Lumen pH',fontsize=7)
ax16.set_ylabel('b$_6$f Rate\n (s$^-$$^1$)',fontsize=7)
ax19.set_ylabel('NPQ',fontsize=7)
ax3b.set_ylabel('Intensity',fontsize=7)
ax6b.set_ylabel('Intensity',fontsize=7)
ax9b.set_ylabel('Intensity',fontsize=7)
ax12b.set_ylabel('Intensity',fontsize=7)
ax15b.set_ylabel('Intensity',fontsize=7)
ax18b.set_ylabel('Intensity',fontsize=7)
ax21b.set_ylabel('Intensity',fontsize=7)

axes = [ax1,ax2,ax3,ax4,ax5,ax6,ax7,ax8,ax9,ax10,ax11,ax12,ax13,
ax14,ax15,ax16,ax17,ax18,
        ax1b,ax2b,ax3b,ax4b,ax5b,ax6b,ax7b,ax8b,ax9b,ax10b,ax11b
,ax12b,ax13b,ax14b,ax15b,
        ax16b,ax17b,ax18b,ax19b,ax20b,ax21b]
for i in range(len(axes)):
    subplot=axes[i]
    subplot.set_xticklabels([])
axes = [ax2,ax3,ax5,ax6,ax8,ax9,ax11,ax12,ax14,ax15,ax17,ax18,ax
20,ax21,
        ax1b,ax2b,ax4b,ax5b,ax7b,ax8b,ax10b,ax11b,ax13b,ax14b,ax
16b,ax17b,ax19b,ax20b]
for i in range(len(axes)):
    subplot=axes[i]
    subplot.set_yticklabels([])
```

```
props = dict(boxstyle='circle', facecolor='white')
labels = ['A1','B1','C1']
axes = [ax1,ax2,ax3]
for i in range(len(axes)):
    subplot=axes[i]
    label=labels[i]
    subplot.text(-2, .45, label, fontsize=6,verticalalignment='top', bbox=props)
    subplot.set_ylim(bottom=0, top=0.28)
axes = [ax4,ax5,ax6]
labels = ['A2','B2','C2']
for i in range(len(axes)):
    subplot=axes[i]
    label=labels[i]
    subplot.text(-2, .25, label, fontsize=6,verticalalignment='top', bbox=props)
    subplot.set_ylim(bottom=-0.02, top=0.15)
axes = [ax7,ax8,ax9]
labels = ['A3','B3','C3']
for i in range(len(axes)):
    subplot=axes[i]
    label=labels[i]
    subplot.text(-2, .25, label, fontsize=6,verticalalignment='top', bbox=props)
    subplot.set_ylim(bottom=-0.05, top=0.15)
axes = [ax10,ax11,ax12]
labels = ['A4','B4','C4']
for i in range(len(axes)):
    subplot=axes[i]
    label=labels[i]
    subplot.text(-2, .08, label, fontsize=6,verticalalignment='top', bbox=props)
    subplot.set_ylim(bottom=-0.01, top=0.05)
axes = [ax13,ax14,ax15]
labels = ['A5','B5','C5']
for i in range(len(axes)):
    subplot=axes[i]
    label=labels[i]
    subplot.text(-2, 7.8, label, fontsize=6,verticalalignment='top', bbox=props)
    subplot.set_ylim(bottom=5.5, top=7)
axes = [ax16,ax17,ax18]
labels = ['A6','B6','C6']
for i in range(len(axes)):
    subplot=axes[i]
```

```
    label=labels[i]
    subplot.text(-2, 550, label, fontsize=6,verticalalignment='t
op', bbox=props)
    subplot.set_ylim(bottom=0, top=350)
axes = [ax19,ax20,ax21]
labels = ['A7','B7','C7']
for i in range(len(axes)):
    subplot=axes[i]
    label=labels[i]
    subplot.text(-2, 7.5, label, fontsize=6,verticalalignment='t
op', bbox=props)
    subplot.set_ylim(bottom=0, top=5)
    subplot.set_xlabel('Time (min)',fontsize=7)

axes = [ax1,ax4,ax7,ax10,ax13,ax16,ax19]
for i in range(len(axes)):
    subplot=axes[i]
    subplot.tick_params(axis='y',labelsize='small')

axes = [ax1b,ax2b,ax3b,ax4b,ax5b,ax6b,ax7b,
        ax8b,ax9b,ax10b,ax11b,ax12b,ax13b,ax14b,
        ax15b,ax16b,ax17b,ax18b,ax19b,ax20b,ax21b]
for i in range(len(axes)):
    subplot = axes[i]
    subplot.set_ylim(bottom=0, top=425)
    subplot.yaxis.label.set_color('red')
    subplot.spines['right'].set_color('red')
    subplot.tick_params(axis='y', colors='red')
    if i not in [2,5,8,11,14,17,20]:
        subplot.set_yticklabels([])

plt.tight_layout(pad=1, w_pad=0.5, h_pad=0)
plt.show()
print('Simulated responses of the light reactions were performed
as in Davis et al., 2017, with all standard '
      'conditions held constant except for the number of ATP syn
thase c-subunits. Simulations were performed using '
      '1-hour of either static light (A), sinusoidal light (B),
or square wave fluctuating light (C) with equal '
      'total photon flux over the total duration of each light t
reatment. Intervals of light excitation are '
      'indicated by shaded regions. (Panels 1-4) The light—induc
ed pmf of ATP synthases with c—stoichiometries of '
      '13 (orange) or 14 (black) are shown in units of volts, so
that a ΔpH of one is equivalent to 0.06 V. The '
```

```
      'total pmf (panel 2), Δψ (panel 3), and ΔpH (panel 4) are
shown as light—induced changes relative to the pmf '
      'dark values indicated as ΔV from dark values, to emphasiz
e light—induced ATP synthase constraints. (5) '
      'Light—induced changes in lumen pH due to photosynthetic a
ctivity. Light intensities and c—ring composition '
      'as in (1). (6) The relative rate constant for plastoquino
l oxidation at the cytochrome b6f complex and (7) '
      'the extent of nonphotochemical quenching qE component for
each c—ring size due to the light—induced changes '
      'in lumen pH.')
```

Supplemental Figure 5: Altered ATP synthase c13 stoichiometry impacts pmf composition and pH—mediated regulatory processes during photosynthesis under increasingly dynamic light environments.

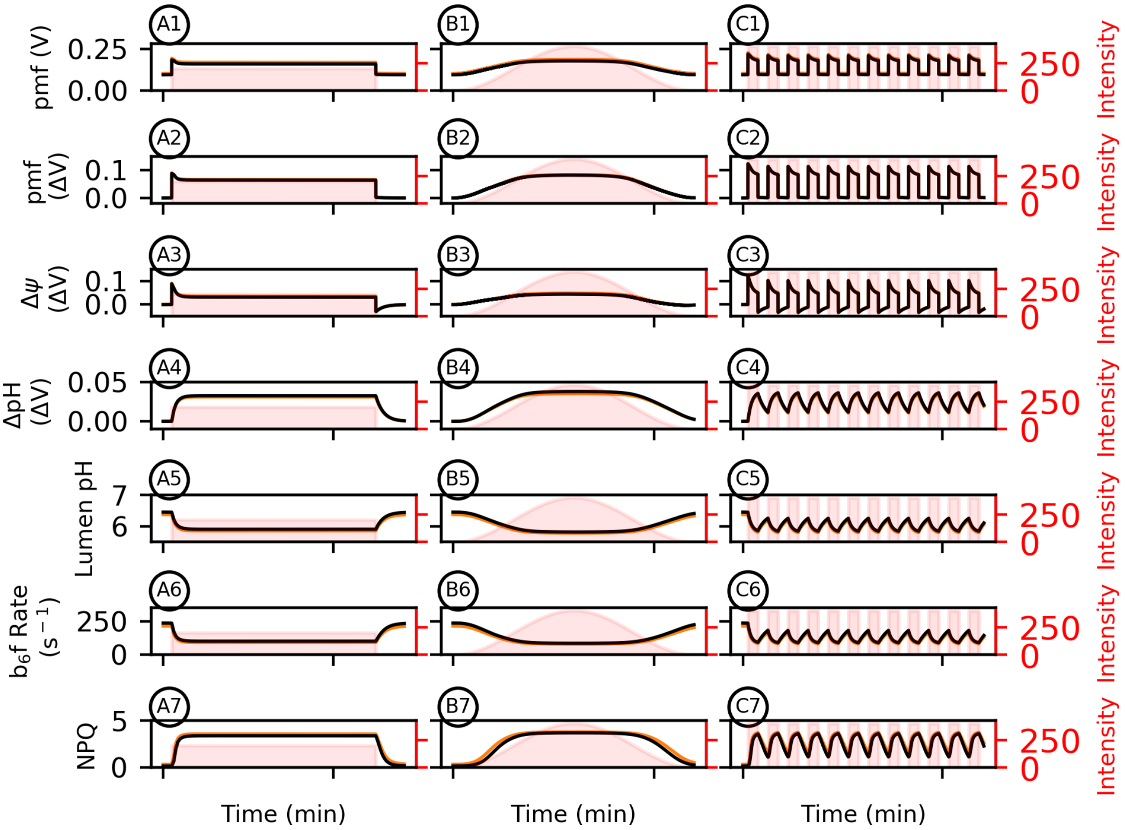

Simulated responses of the light reactions were perf
ormed as in Davis et al., 2017, with all standard co
nditions held constant except for the number of ATP
synthase c-subunits. Simulations were performed usin
g 1-hour of either static light (A), sinusoidal ligh
t (B), or square wave fluctuating light (C) with equ
al total photon flux over the total duration of each
light treatment. Intervals of light excitation are i
ndicated by shaded regions. (Panels 1-4) The light–i
nduced pmf of ATP synthases with c–stoichiometries o
f 13 (orange) or 14 (black) are shown in units of vo
lts, so that a ΔpH of one is equivalent to 0.06 V. T
he total pmf (panel 2), Δψ (panel 3), and ΔpH (panel
4) are shown as light–induced changes relative to th
e pmf dark values indicated as ΔV from dark values,
to emphasize light–induced ATP synthase constraints.
(5) Light–induced changes in lumen pH due to photosy
nthetic activity. Light intensities and c–ring compo
sition as in (1). (6) The relative rate constant for
plastoquinol oxidation at the cytochrome b6f complex
and (7) the extent of nonphotochemical quenching qE
component for each c–ring size due to the light–indu
ced changes in lumen pH.

In [65]:

```
print('Supplemental Figure 6:  Altered ATP synthase c15 stoichio
metry impacts pmf composition and pH–mediated '
      'regulatory processes during photosynthesis under increasi
ngly dynamic light environments.')

fig = plt.figure('Supplemental Figure 6', figsize=(5,4), dpi=200
)
ax1 = fig.add_subplot(8,3,1)
ax2 = fig.add_subplot(8,3,2)
ax3 = fig.add_subplot(8,3,3)
ax4 = fig.add_subplot(8,3,4)
ax5 = fig.add_subplot(8,3,5)
ax6 = fig.add_subplot(8,3,6)
ax7 = fig.add_subplot(8,3,7)
ax8 = fig.add_subplot(8,3,8)
ax9 = fig.add_subplot(8,3,9)
ax10 = fig.add_subplot(8,3,10)
ax11 = fig.add_subplot(8,3,11)
[illegible]
```

```
ax12 = fig.add_subplot(8,3,12)
ax13 = fig.add_subplot(8,3,13)

ax14 = fig.add_subplot(8,3,14)
ax15 = fig.add_subplot(8,3,15)
ax16 = fig.add_subplot(8,3,16)
ax17 = fig.add_subplot(8,3,17)
ax18 = fig.add_subplot(8,3,18)
ax19 = fig.add_subplot(8,3,19)
ax20 = fig.add_subplot(8,3,20)
ax21 = fig.add_subplot(8,3,21)

ax1b = ax1.twinx()
ax2b = ax2.twinx()
ax3b = ax3.twinx()
ax4b = ax4.twinx()
ax5b = ax5.twinx()
ax6b = ax6.twinx()
ax7b = ax7.twinx()
ax8b = ax8.twinx()
ax9b = ax9.twinx()
ax10b = ax10.twinx()
ax11b = ax11.twinx()
ax12b = ax12.twinx()
ax13b = ax13.twinx()
ax14b = ax14.twinx()
ax15b = ax15.twinx()
ax16b = ax16.twinx()
ax17b = ax17.twinx()
ax18b = ax18.twinx()
ax19b = ax19.twinx()
ax20b = ax20.twinx()
ax21b = ax21.twinx()

for key in output_dict:
    c=key.split('=')[1]
    if c == "15":
        if "single pulse 201.3umolE subunits" in str(key):
            ax1.plot(output_dict[key]['time_axis']/60,output_dic
t[key]['pmf'],
                      color=set_of_c_stoichiometries[int(c)],line
width=1)
            ax4.plot(output_dict[key]['time_axis']/60,output_dic
t[key]['pmf_offset'],
                      color=set_of_c_stoichiometries[int(c)],line
width=1)
```

```
            ax7.plot(output_dict[key]['time_axis']/60,output_dic
t[key]['Dy_offset'],
                     color=set_of_c_stoichiometries[int(c)],line
width=1)
            ax10.plot(output_dict[key]['time_axis']/60,output_di
ct[key]['delta_pH_V_offset'],
                      color=set_of_c_stoichiometries[int(c)],lin
ewidth=1)
            ax13.plot(output_dict[key]['time_axis']/60,output_di
ct[key]['pHlumen'],
                      color=set_of_c_stoichiometries[int(c)],lin
ewidth=1)
            ax16.plot(output_dict[key]['time_axis']/60,output_di
ct[key]['b6f_control'],
                      color=set_of_c_stoichiometries[int(c)],lin
ewidth=1)
            ax19.plot(output_dict[key]['time_axis']/60,output_di
ct[key]['NPQ_array'],
                      color=set_of_c_stoichiometries[int(c)],lin
ewidth=1)


            ax1b.fill_between(output_dict[key]['time_axis']/60,
output_dict[key]['light_curve'],0,
                              color='red', alpha=0.1,zorder=2)
            ax4b.fill_between(output_dict[key]['time_axis']/60,
output_dict[key]['light_curve'],0,
                              color='red', alpha=0.1,zorder=2)
            ax7b.fill_between(output_dict[key]['time_axis']/60,
output_dict[key]['light_curve'],0,
                              color='red', alpha=0.1,zorder=2)
            ax10b.fill_between(output_dict[key]['time_axis']/60,
output_dict[key]['light_curve'],0,
                               color='red', alpha=0.1,zorder=2)
            ax13b.fill_between(output_dict[key]['time_axis']/60,
output_dict[key]['light_curve'],0,
                               color='red', alpha=0.1,zorder=2)
            ax16b.fill_between(output_dict[key]['time_axis']/60,
output_dict[key]['light_curve'],0,
                               color='red', alpha=0.1,zorder=2)
            ax19b.fill_between(output_dict[key]['time_axis']/60,
output_dict[key]['light_curve'],0,
                               color='red', alpha=0.1,zorder=2)
        if "single sin wave 402.71umolE subunits" in str(key):
            ax2.plot(output_dict[key]['time_axis']/60,output_dic
```

```
t[key]['pmf'],
                     color=set_of_c_stoichiometries[int(c)],line
width=1)
            ax5.plot(output_dict[key]['time_axis']/60,output_dic
t[key]['pmf_offset'],
                     color=set_of_c_stoichiometries[int(c)],line
width=1)
            ax8.plot(output_dict[key]['time_axis']/60,output_dic
t[key]['Dy_offset'],
                     color=set_of_c_stoichiometries[int(c)],line
width=1)
            ax11.plot(output_dict[key]['time_axis']/60,output_di
ct[key]['delta_pH_V_offset'],
                      color=set_of_c_stoichiometries[int(c)],lin
ewidth=1)
            ax14.plot(output_dict[key]['time_axis']/60,output_di
ct[key]['pHlumen'],
                      color=set_of_c_stoichiometries[int(c)],lin
ewidth=1)
            ax17.plot(output_dict[key]['time_axis']/60,output_di
ct[key]['b6f_control'],
                      color=set_of_c_stoichiometries[int(c)],lin
ewidth=1)
            ax20.plot(output_dict[key]['time_axis']/60,output_di
ct[key]['NPQ_array'],
                      color=set_of_c_stoichiometries[int(c)],lin
ewidth=1)



            ax2b.fill_between(output_dict[key]['time_axis']/60,
output_dict[key]['light_curve'],0,
                              color='red', alpha=0.1,zorder=2)
            ax5b.fill_between(output_dict[key]['time_axis']/60,
output_dict[key]['light_curve'],0,
                              color='red', alpha=0.1,zorder=2)
            ax8b.fill_between(output_dict[key]['time_axis']/60,
output_dict[key]['light_curve'],0,
                              color='red', alpha=0.1,zorder=2)
            ax11b.fill_between(output_dict[key]['time_axis']/60,
output_dict[key]['light_curve'],0,
                               color='red', alpha=0.1,zorder=2)
            ax14b.fill_between(output_dict[key]['time_axis']/60,
output_dict[key]['light_curve'],0,
                               color='red', alpha=0.1,zorder=2)
            ax17b.fill_between(output_dict[key]['time_axis']/60,
```

```
output_dict[key]['light_curve'],0,
                                color='red', alpha=0.1,zorder=2)
            ax20b.fill_between(output_dict[key]['time_axis']/60,
output_dict[key]['light_curve'],0,
                                color='red', alpha=0.1,zorder=2)

        if "square wave 60 min max 400 light subunits" in str(ke
y):
            ax3.plot(output_dict[key]['time_axis']/60,output_dic
t[key]['pmf'],
                     color=set_of_c_stoichiometries[int(c)],line
width=1)
            ax6.plot(output_dict[key]['time_axis']/60,output_dic
t[key]['pmf_offset'],
                     color=set_of_c_stoichiometries[int(c)],line
width=1)
            ax9.plot(output_dict[key]['time_axis']/60,output_dic
t[key]['Dy_offset'],
                     color=set_of_c_stoichiometries[int(c)],line
width=1)
            ax12.plot(output_dict[key]['time_axis']/60,output_di
ct[key]['delta_pH_V_offset'],
                      color=set_of_c_stoichiometries[int(c)],lin
ewidth=1)
            ax15.plot(output_dict[key]['time_axis']/60,output_di
ct[key]['pHlumen'],
                      color=set_of_c_stoichiometries[int(c)],lin
ewidth=1)
            ax18.plot(output_dict[key]['time_axis']/60,output_di
ct[key]['b6f_control'],
                      color=set_of_c_stoichiometries[int(c)],lin
ewidth=1)
            ax21.plot(output_dict[key]['time_axis']/60,output_di
ct[key]['NPQ_array'],
                      color=set_of_c_stoichiometries[int(c)],lin
ewidth=1)


            ax3b.fill_between(output_dict[key]['time_axis']/60,
output_dict[key]['light_curve'],0,
                               color='red', alpha=0.1,zorder=2)
            ax6b.fill_between(output_dict[key]['time_axis']/60,
output_dict[key]['light_curve'],0,
                               color='red', alpha=0.1,zorder=2)
            ax9b.fill_between(output_dict[key]['time_axis']/60,
```

```
output_dict[key]['light_curve'],0,
                              color='red', alpha=0.1,zorder=2)
            ax12b.fill_between(output_dict[key]['time_axis']/60,
output_dict[key]['light_curve'],0,
                               color='red', alpha=0.1,zorder=2)
            ax15b.fill_between(output_dict[key]['time_axis']/60,
output_dict[key]['light_curve'],0,
                               color='red', alpha=0.1,zorder=2)
            ax18b.fill_between(output_dict[key]['time_axis']/60,
output_dict[key]['light_curve'],0,
                               color='red', alpha=0.11,zorder=2)
            ax21b.fill_between(output_dict[key]['time_axis']/60,
output_dict[key]['light_curve'],0,
                               color='red', alpha=0.1,zorder=2)
    if c == "14":
        if "single pulse 201.3umolE subunits" in str(key):
            ax1.plot(output_dict[key]['time_axis']/60,output_dic
t[key]['pmf'],
                     color=set_of_c_stoichiometries[int(c)],line
width=1)
            ax4.plot(output_dict[key]['time_axis']/60,output_dic
t[key]['pmf_offset'],
                     color=set_of_c_stoichiometries[int(c)],line
width=1)
            ax7.plot(output_dict[key]['time_axis']/60,output_dic
t[key]['Dy_offset'],
                     color=set_of_c_stoichiometries[int(c)],line
width=1)
            ax10.plot(output_dict[key]['time_axis']/60,output_di
ct[key]['delta_pH_V_offset'],
                      color=set_of_c_stoichiometries[int(c)],lin
ewidth=1)
            ax13.plot(output_dict[key]['time_axis']/60,output_di
ct[key]['pHlumen'],
                      color=set_of_c_stoichiometries[int(c)],lin
ewidth=1)
            ax16.plot(output_dict[key]['time_axis']/60,output_di
ct[key]['b6f_control'],
                      color=set_of_c_stoichiometries[int(c)],lin
ewidth=1)
            ax19.plot(output_dict[key]['time_axis']/60,output_di
ct[key]['NPQ_array'],
                      color=set_of_c_stoichiometries[int(c)],lin
ewidth=1)
        if "single sin wave 402.71umolE subunits" in str(key):
```

```
            ax2.plot(output_dict[key]['time_axis']/60,output_dic
t[key]['pmf'],
                     color=set_of_c_stoichiometries[int(c)],line
width=1)
            ax5.plot(output_dict[key]['time_axis']/60,output_dic
t[key]['pmf_offset'],
                     color=set_of_c_stoichiometries[int(c)],line
width=1)
            ax8.plot(output_dict[key]['time_axis']/60,output_dic
t[key]['Dy_offset'],
                     color=set_of_c_stoichiometries[int(c)],line
width=1)
            ax11.plot(output_dict[key]['time_axis']/60,output_di
ct[key]['delta_pH_V_offset'],
                      color=set_of_c_stoichiometries[int(c)],lin
ewidth=1)
            ax14.plot(output_dict[key]['time_axis']/60,output_di
ct[key]['pHlumen'],
                      color=set_of_c_stoichiometries[int(c)],lin
ewidth=1)
            ax17.plot(output_dict[key]['time_axis']/60,output_di
ct[key]['b6f_control'],
                      color=set_of_c_stoichiometries[int(c)],lin
ewidth=1)
            ax20.plot(output_dict[key]['time_axis']/60,output_di
ct[key]['NPQ_array'],

                      color=set_of_c_stoichiometries[int(c)],lin
ewidth=1)
        if "square wave 60 min max 400 light subunits" in str(ke
y):
            ax3.plot(output_dict[key]['time_axis']/60,output_dic
t[key]['pmf'],
                     color=set_of_c_stoichiometries[int(c)],line
width=1)
            ax6.plot(output_dict[key]['time_axis']/60,output_dic
t[key]['pmf_offset'],
                     color=set_of_c_stoichiometries[int(c)],line
width=1)
            ax9.plot(output_dict[key]['time_axis']/60,output_dic
t[key]['Dy_offset'],
                     color=set_of_c_stoichiometries[int(c)],line
width=1)
            ax12.plot(output_dict[key]['time_axis']/60,output_di
ct[key]['delta_pH_V_offset'],
```

```
                         color=set_of_c_stoichiometries[int(c)],lin
ewidth=1)
            ax15.plot(output_dict[key]['time_axis']/60,output_di
ct[key]['pHlumen'],
                         color=set_of_c_stoichiometries[int(c)],lin
ewidth=1)
            ax18.plot(output_dict[key]['time_axis']/60,output_di
ct[key]['b6f_control'],
                         color=set_of_c_stoichiometries[int(c)],lin
ewidth=1)
            ax21.plot(output_dict[key]['time_axis']/60,output_di
ct[key]['NPQ_array'],
                         color=set_of_c_stoichiometries[int(c)],lin
ewidth=1)

ax1.set_ylabel('pmf (V)',fontsize=7)
ax4.set_ylabel('pmf\n ($\Delta$V)',fontsize=7)
ax7.set_ylabel('$\Delta$$\psi$\n ($\Delta$V)',fontsize=7)
ax10.set_ylabel('$\Delta$pH\n ($\Delta$V)',fontsize=7)
ax13.set_ylabel('Lumen pH',fontsize=7)
ax16.set_ylabel('b$_6$f Rate\n (s$^-$$^1$)',fontsize=7)
ax19.set_ylabel('NPQ',fontsize=7)
ax3b.set_ylabel('Intensity',fontsize=7)
ax6b.set_ylabel('Intensity',fontsize=7)
ax9b.set_ylabel('Intensity',fontsize=7)
ax12b.set_ylabel('Intensity',fontsize=7)
ax15b.set_ylabel('Intensity',fontsize=7)
ax18b.set_ylabel('Intensity',fontsize=7)
ax21b.set_ylabel('Intensity',fontsize=7)

axes = [ax1,ax2,ax3,ax4,ax5,ax6,ax7,ax8,ax9,ax10,ax11,ax12,ax13,
ax14,ax15,ax16,ax17,ax18,
        ax1b,ax2b,ax3b,ax4b,ax5b,ax6b,ax7b,ax8b,ax9b,ax10b,ax11b
,ax12b,ax13b,ax14b,ax15b,
        ax16b,ax17b,ax18b,ax19b,ax20b,ax21b]
for i in range(len(axes)):
    subplot=axes[i]
    subplot.set_xticklabels([])
axes = [ax2,ax3,ax5,ax6,ax8,ax9,ax11,ax12,ax14,ax15,ax17,ax18,ax
20,ax21,
        ax1b,ax2b,ax4b,ax5b,ax7b,ax8b,ax10b,ax11b,ax13b,ax14b,ax
16b,ax17b,ax19b,ax20b]
for i in range(len(axes)):
    subplot=axes[i]
    subplot.set_yticklabels([])
```

```
[illegible]

props = dict(boxstyle='circle', facecolor='white')
labels = ['A1','B1','C1']
axes = [ax1,ax2,ax3]
for i in range(len(axes)):
    subplot=axes[i]
    label=labels[i]
    subplot.text(-2, .45, label, fontsize=6,verticalalignment='t
op', bbox=props)
    subplot.set_ylim(bottom=0, top=0.28)
axes = [ax4,ax5,ax6]
labels = ['A2','B2','C2']
for i in range(len(axes)):
    subplot=axes[i]
    label=labels[i]
    subplot.text(-2, .25, label, fontsize=6,verticalalignment='t
op', bbox=props)
    subplot.set_ylim(bottom=-0.02, top=0.15)
axes = [ax7,ax8,ax9]
labels = ['A3','B3','C3']
for i in range(len(axes)):
    subplot=axes[i]
    label=labels[i]
    subplot.text(-2, .25, label, fontsize=6,verticalalignment='t
op', bbox=props)
    subplot.set_ylim(bottom=-0.05, top=0.15)
axes = [ax10,ax11,ax12]
labels = ['A4','B4','C4']
for i in range(len(axes)):
    subplot=axes[i]
    label=labels[i]
    subplot.text(-2, .08, label, fontsize=6,verticalalignment='t
op', bbox=props)
    subplot.set_ylim(bottom=-0.01, top=0.05)
axes = [ax13,ax14,ax15]
labels = ['A5','B5','C5']
for i in range(len(axes)):
    subplot=axes[i]
    label=labels[i]
    subplot.text(-2, 7.8, label, fontsize=6,verticalalignment='t
op', bbox=props)
    subplot.set_ylim(bottom=5.5, top=7)
axes = [ax16,ax17,ax18]
labels = ['A6','B6','C6']
for i in range(len(axes)):
```

```python
    subplot=axes[i]
    label=labels[i]
    subplot.text(-2, 550, label, fontsize=6,verticalalignment='top', bbox=props)
    subplot.set_ylim(bottom=0, top=350)
axes = [ax19,ax20,ax21]
labels = ['A7','B7','C7']
for i in range(len(axes)):
    subplot=axes[i]
    label=labels[i]
    subplot.text(-2, 7.5, label, fontsize=6,verticalalignment='top', bbox=props)
    subplot.set_ylim(bottom=0, top=5)
    subplot.set_xlabel('Time (min)',fontsize=7)

axes = [ax1,ax4,ax7,ax10,ax13,ax16,ax19]
for i in range(len(axes)):
    subplot=axes[i]
    subplot.tick_params(axis='y',labelsize='small')

axes = [ax1b,ax2b,ax3b,ax4b,ax5b,ax6b,ax7b,
        ax8b,ax9b,ax10b,ax11b,ax12b,ax13b,ax14b,
        ax15b,ax16b,ax17b,ax18b,ax19b,ax20b,ax21b]
for i in range(len(axes)):
    subplot = axes[i]
    subplot.set_ylim(bottom=0, top=425)
    subplot.yaxis.label.set_color('red')
    subplot.spines['right'].set_color('red')
    subplot.tick_params(axis='y', colors='red')
    if i not in [2,5,8,11,14,17,20]:
        subplot.set_yticklabels([])

plt.tight_layout(pad=1, w_pad=0.5, h_pad=0)
plt.show()
print('Simulated responses of the light reactions were performed as in Davis et al., 2017, with all standard '
      'conditions held constant except for the number of ATP synthase c-subunits. Simulations were performed using '
      '1-hour of either static light (A), sinusoidal light (B), or square wave fluctuating light (C) with equal '
      'total photon flux over the total duration of each light treatment. Intervals of light excitation are '
      'indicated by shaded regions. (Panels 1-4) The light-induced pmf of ATP synthases with c-stoichiometries of '
      '15 (cyan) or 14 (black) are shown in units of volts, so t
```

```
hat a ΔpH of one is equivalent to 0.06 V. The total '
      'pmf (panel 2), Δψ (panel 3), and ΔpH (panel 4) are shown
as light—induced changes relative to the pmf dark '
      'values indicated as ΔV from dark values, to emphasize lig
ht—induced ATP synthase constraints. (5) '
      'Light—induced changes in lumen pH due to photosynthetic a
ctivity. Light intensities and c—ring composition '
      'as in (1). (6) The relative rate constant for plastoquino
l oxidation at the cytochrome b6f complex and (7) '
      'the extent of nonphotochemical quenching qE component for
each c—ring size due to the light—induced changes '
      'in lumen pH.')
```

```
Supplemental Figure 6:  Altered ATP synthase c15 sto
ichiometry impacts pmf composition and pH—mediated r
egulatory processes during photosynthesis under incr
easingly dynamic light environments.
```

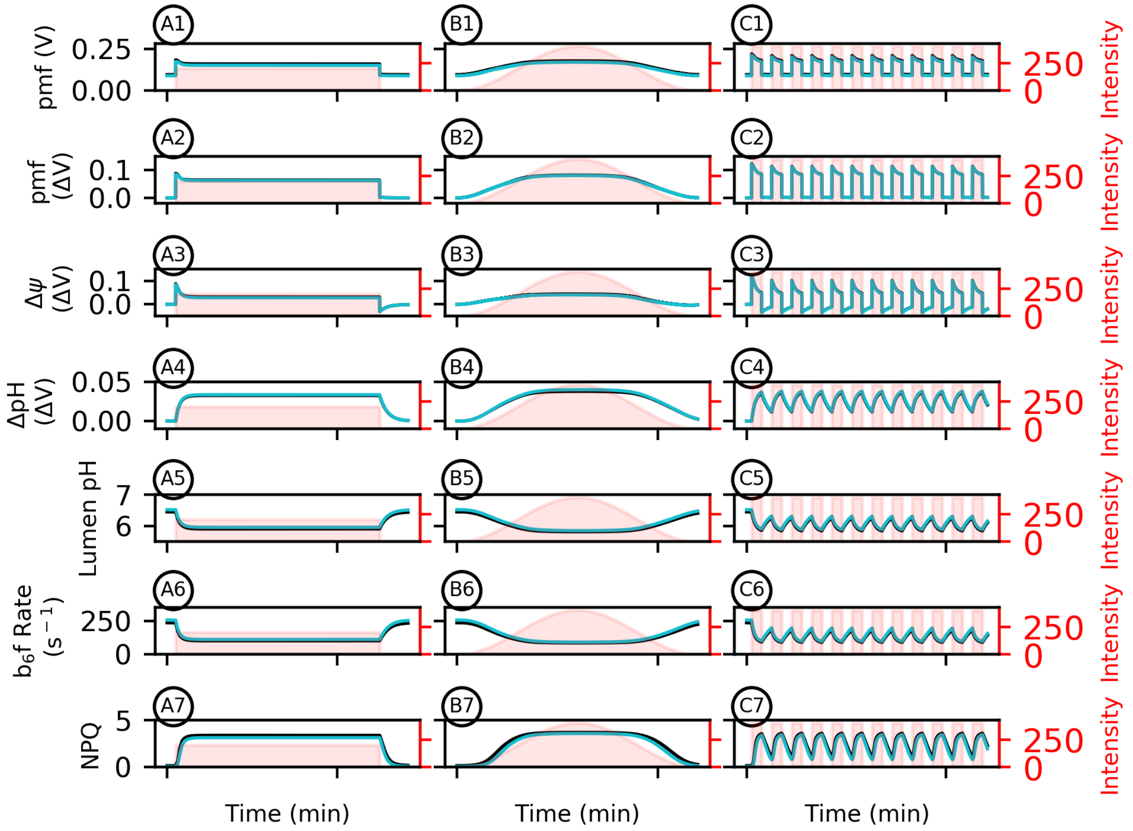
A1
B1
C1
A2
B2
C2
A3
B3
C3
A4
B4
C4
A5
B5
C5
A6
B6
C6
A7
B7
C7
pmf (V)
0.25
0.00
pmf
(ΔV)
0.1
0.0
Δψ
(ΔV)
0.1
0.0
ΔpH
(ΔV)
0.05
0.00
Lumen pH
7
6
b6f Rate
(s⁻¹)
250
0
NPQ
5
0
250
0
Intensity
Time (min)
Time (min)
Time (min)

Simulated responses of the light reactions were performed as in Davis et al., 2017, with all standard conditions held constant except for the number of ATP synthase c-subunits. Simulations were performed using 1-hour of either static light (A), sinusoidal light (B), or square wave fluctuating light (C) with equal total photon flux over the total duration of each light treatment. Intervals of light excitation are indicated by shaded regions. (Panels 1-4) The light–induced pmf of ATP synthases with c–stoichiometries of 15 (cyan) or 14 (black) are shown in units of volts, so that a ΔpH of one is equivalent to 0.06 V. The total pmf (panel 2), Δψ (panel 3), and ΔpH (panel 4) are shown as light–induced changes relative to the pmf dark values indicated as ΔV from dark values, to emphasize light–induced ATP synthase constraints. (5) Light–induced changes in lumen pH due to photosynthetic activity. Light intensities and c–ring composition as in (1). (6) The relative rate constant for plastoquinol oxidation at the cytochrome b6f complex and (7) the extent of nonphotochemical quenching qE component for each c–ring size due to the light–induced changes in lumen pH.

In [66]:

```
print('Supplemental Figure 7:  Altered ATP synthase c17 stoichiometry impacts pmf composition and pH–mediated '
      'regulatory processes during photosynthesis under increasingly dynamic light environments.')

fig = plt.figure('Supplemental Figure 7', figsize=(5,4), dpi=200)
ax1 = fig.add_subplot(8,3,1)
ax2 = fig.add_subplot(8,3,2)
ax3 = fig.add_subplot(8,3,3)
ax4 = fig.add_subplot(8,3,4)
ax5 = fig.add_subplot(8,3,5)
ax6 = fig.add_subplot(8,3,6)
ax7 = fig.add_subplot(8,3,7)
ax8 = fig.add_subplot(8,3,8)
ax9 = fig.add_subplot(8,3,9)
ax10 = fig.add_subplot(8,3,10)
ax11 = fig.add_subplot(8,3,11)
ax12 = fig.add_subplot(8,3,12)
```

```
ax12 = fig.add_subplot(8,3,12)
ax13 = fig.add_subplot(8,3,13)
ax14 = fig.add_subplot(8,3,14)
ax15 = fig.add_subplot(8,3,15)
ax16 = fig.add_subplot(8,3,16)
ax17 = fig.add_subplot(8,3,17)
ax18 = fig.add_subplot(8,3,18)
ax19 = fig.add_subplot(8,3,19)
ax20 = fig.add_subplot(8,3,20)
ax21 = fig.add_subplot(8,3,21)

ax1b = ax1.twinx()
ax2b = ax2.twinx()
ax3b = ax3.twinx()
ax4b = ax4.twinx()
ax5b = ax5.twinx()
ax6b = ax6.twinx()
ax7b = ax7.twinx()
ax8b = ax8.twinx()
ax9b = ax9.twinx()
ax10b = ax10.twinx()
ax11b = ax11.twinx()
ax12b = ax12.twinx()
ax13b = ax13.twinx()
ax14b = ax14.twinx()
ax15b = ax15.twinx()
ax16b = ax16.twinx()
ax17b = ax17.twinx()
ax18b = ax18.twinx()
ax19b = ax19.twinx()
ax20b = ax20.twinx()
ax21b = ax21.twinx()

for key in output_dict:
    c=key.split('=')[1]
    if c == "17":
        if "single pulse 201.3umolE subunits" in str(key):
            ax1.plot(output_dict[key]['time_axis']/60,output_dic
t[key]['pmf'],
                      color=set_of_c_stoichiometries[int(c)],line
width=1)
            ax4.plot(output_dict[key]['time_axis']/60,output_dic
t[key]['pmf_offset'],
                      color=set_of_c_stoichiometries[int(c)],line
width=1)
```

```
            ax7.plot(output_dict[key]['time_axis']/60,output_dic
t[key]['Dy_offset'],
                     color=set_of_c_stoichiometries[int(c)],line
width=1)
            ax10.plot(output_dict[key]['time_axis']/60,output_di
ct[key]['delta_pH_V_offset'],
                      color=set_of_c_stoichiometries[int(c)],lin
ewidth=1)
            ax13.plot(output_dict[key]['time_axis']/60,output_di
ct[key]['pHlumen'],
                      color=set_of_c_stoichiometries[int(c)],lin
ewidth=1)
            ax16.plot(output_dict[key]['time_axis']/60,output_di
ct[key]['b6f_control'],
                      color=set_of_c_stoichiometries[int(c)],lin
ewidth=1)
            ax19.plot(output_dict[key]['time_axis']/60,output_di
ct[key]['NPQ_array'],
                      color=set_of_c_stoichiometries[int(c)],lin
ewidth=1)


            ax1b.fill_between(output_dict[key]['time_axis']/60,
output_dict[key]['light_curve'],0,
                              color='red', alpha=0.1,zorder=2)
            ax4b.fill_between(output_dict[key]['time_axis']/60,
output_dict[key]['light_curve'],0,
                              color='red', alpha=0.1,zorder=2)
            ax7b.fill_between(output_dict[key]['time_axis']/60,
output_dict[key]['light_curve'],0,
                              color='red', alpha=0.1,zorder=2)
            ax10b.fill_between(output_dict[key]['time_axis']/60,
output_dict[key]['light_curve'],0,
                               color='red', alpha=0.1,zorder=2)
            ax13b.fill_between(output_dict[key]['time_axis']/60,
output_dict[key]['light_curve'],0,
                               color='red', alpha=0.1,zorder=2)
            ax16b.fill_between(output_dict[key]['time_axis']/60,
output_dict[key]['light_curve'],0,
                               color='red', alpha=0.1,zorder=2)
            ax19b.fill_between(output_dict[key]['time_axis']/60,
output_dict[key]['light_curve'],0,
                               color='red', alpha=0.1,zorder=2)
        if "single sin wave 402.71umolE subunits" in str(key):
            ax2.plot(output_dict[key]['time_axis']/60,output_dic
```

```
t[key]['pmf'],
                     color=set_of_c_stoichiometries[int(c)],line
width=1)
            ax5.plot(output_dict[key]['time_axis']/60,output_dic
t[key]['pmf_offset'],
                     color=set_of_c_stoichiometries[int(c)],line
width=1)
            ax8.plot(output_dict[key]['time_axis']/60,output_dic
t[key]['Dy_offset'],
                     color=set_of_c_stoichiometries[int(c)],line
width=1)
            ax11.plot(output_dict[key]['time_axis']/60,output_di
ct[key]['delta_pH_V_offset'],
                      color=set_of_c_stoichiometries[int(c)],lin
ewidth=1)
            ax14.plot(output_dict[key]['time_axis']/60,output_di
ct[key]['pHlumen'],
                      color=set_of_c_stoichiometries[int(c)],lin
ewidth=1)
            ax17.plot(output_dict[key]['time_axis']/60,output_di
ct[key]['b6f_control'],
                      color=set_of_c_stoichiometries[int(c)],lin
ewidth=1)
            ax20.plot(output_dict[key]['time_axis']/60,output_di
ct[key]['NPQ_array'],
                      color=set_of_c_stoichiometries[int(c)],lin
ewidth=1)



            ax2b.fill_between(output_dict[key]['time_axis']/60,
output_dict[key]['light_curve'],0,
                              color='red', alpha=0.1,zorder=2)
            ax5b.fill_between(output_dict[key]['time_axis']/60,
output_dict[key]['light_curve'],0,
                              color='red', alpha=0.1,zorder=2)
            ax8b.fill_between(output_dict[key]['time_axis']/60,
output_dict[key]['light_curve'],0,
                              color='red', alpha=0.1,zorder=2)
            ax11b.fill_between(output_dict[key]['time_axis']/60,
output_dict[key]['light_curve'],0,
                               color='red', alpha=0.1,zorder=2)
            ax14b.fill_between(output_dict[key]['time_axis']/60,
output_dict[key]['light_curve'],0,
                               color='red', alpha=0.1,zorder=2)
            ax17b.fill_between(output_dict[key]['time_axis']/60,
```

```
output_dict[key]['light_curve'],0,
                                     color='red', alpha=0.1,zorder=2)
                ax20b.fill_between(output_dict[key]['time_axis']/60,
output_dict[key]['light_curve'],0,
                                     color='red', alpha=0.1,zorder=2)

            if "square wave 60 min max 400 light subunits" in str(ke
y):
                ax3.plot(output_dict[key]['time_axis']/60,output_dic
t[key]['pmf'],
                         color=set_of_c_stoichiometries[int(c)],line
width=1)
                ax6.plot(output_dict[key]['time_axis']/60,output_dic
t[key]['pmf_offset'],
                         color=set_of_c_stoichiometries[int(c)],line
width=1)
                ax9.plot(output_dict[key]['time_axis']/60,output_dic
t[key]['Dy_offset'],
                         color=set_of_c_stoichiometries[int(c)],line
width=1)
                ax12.plot(output_dict[key]['time_axis']/60,output_di
ct[key]['delta_pH_V_offset'],
                          color=set_of_c_stoichiometries[int(c)],lin
ewidth=1)
                ax15.plot(output_dict[key]['time_axis']/60,output_di
ct[key]['pHlumen'],
                          color=set_of_c_stoichiometries[int(c)],lin
ewidth=1)
                ax18.plot(output_dict[key]['time_axis']/60,output_di
ct[key]['b6f_control'],
                          color=set_of_c_stoichiometries[int(c)],lin
ewidth=1)
                ax21.plot(output_dict[key]['time_axis']/60,output_di
ct[key]['NPQ_array'],
                          color=set_of_c_stoichiometries[int(c)],lin
ewidth=1)


                ax3b.fill_between(output_dict[key]['time_axis']/60,
output_dict[key]['light_curve'],0,
                                    color='red', alpha=0.1,zorder=2)
                ax6b.fill_between(output_dict[key]['time_axis']/60,
output_dict[key]['light_curve'],0,
                                    color='red', alpha=0.1,zorder=2)
                ax9b.fill_between(output_dict[key]['time_axis']/60,
```

```
output_dict[key]['light_curve'],0,
                              color='red', alpha=0.1,zorder=2)
            ax12b.fill_between(output_dict[key]['time_axis']/60,
output_dict[key]['light_curve'],0,
                               color='red', alpha=0.1,zorder=2)
            ax15b.fill_between(output_dict[key]['time_axis']/60,
output_dict[key]['light_curve'],0,
                               color='red', alpha=0.1,zorder=2)
            ax18b.fill_between(output_dict[key]['time_axis']/60,
output_dict[key]['light_curve'],0,
                               color='red', alpha=0.11,zorder=2)
            ax21b.fill_between(output_dict[key]['time_axis']/60,
output_dict[key]['light_curve'],0,
                               color='red', alpha=0.1,zorder=2)
    if c == "14":
        if "single pulse 201.3umolE subunits" in str(key):
            ax1.plot(output_dict[key]['time_axis']/60,output_dic
t[key]['pmf'],
                     color=set_of_c_stoichiometries[int(c)],line
width=1)
            ax4.plot(output_dict[key]['time_axis']/60,output_dic
t[key]['pmf_offset'],
                     color=set_of_c_stoichiometries[int(c)],line
width=1)
            ax7.plot(output_dict[key]['time_axis']/60,output_dic
t[key]['Dy_offset'],
                     color=set_of_c_stoichiometries[int(c)],line
width=1)
            ax10.plot(output_dict[key]['time_axis']/60,output_di
ct[key]['delta_pH_V_offset'],
                      color=set_of_c_stoichiometries[int(c)],lin
ewidth=1)
            ax13.plot(output_dict[key]['time_axis']/60,output_di
ct[key]['pHlumen'],
                      color=set_of_c_stoichiometries[int(c)],lin
ewidth=1)
            ax16.plot(output_dict[key]['time_axis']/60,output_di
ct[key]['b6f_control'],
                      color=set_of_c_stoichiometries[int(c)],lin
ewidth=1)
            ax19.plot(output_dict[key]['time_axis']/60,output_di
ct[key]['NPQ_array'],
                      color=set_of_c_stoichiometries[int(c)],lin
ewidth=1)
        if "single sin wave 402.71umolE subunits" in str(key):
```

```
            ax2.plot(output_dict[key]['time_axis']/60,output_dic
t[key]['pmf'],
                     color=set_of_c_stoichiometries[int(c)],line
width=1)
            ax5.plot(output_dict[key]['time_axis']/60,output_dic
t[key]['pmf_offset'],
                     color=set_of_c_stoichiometries[int(c)],line
width=1)
            ax8.plot(output_dict[key]['time_axis']/60,output_dic
t[key]['Dy_offset'],
                     color=set_of_c_stoichiometries[int(c)],line
width=1)
            ax11.plot(output_dict[key]['time_axis']/60,output_di
ct[key]['delta_pH_V_offset'],
                      color=set_of_c_stoichiometries[int(c)],lin
ewidth=1)
            ax14.plot(output_dict[key]['time_axis']/60,output_di
ct[key]['pHlumen'],
                      color=set_of_c_stoichiometries[int(c)],lin
ewidth=1)
            ax17.plot(output_dict[key]['time_axis']/60,output_di
ct[key]['b6f_control'],
                      color=set_of_c_stoichiometries[int(c)],lin
ewidth=1)
            ax20.plot(output_dict[key]['time_axis']/60,output_di
ct[key]['NPQ_array'],
                      color=set_of_c_stoichiometries[int(c)],lin
ewidth=1)
        if "square wave 60 min max 400 light subunits" in str(ke
y):
            ax3.plot(output_dict[key]['time_axis']/60,output_dic
t[key]['pmf'],
                     color=set_of_c_stoichiometries[int(c)],line
width=1)
            ax6.plot(output_dict[key]['time_axis']/60,output_dic
t[key]['pmf_offset'],
                     color=set_of_c_stoichiometries[int(c)],line
width=1)
            ax9.plot(output_dict[key]['time_axis']/60,output_dic
t[key]['Dy_offset'],
                     color=set_of_c_stoichiometries[int(c)],line
width=1)
            ax12.plot(output_dict[key]['time_axis']/60,output_di
ct[key]['delta_pH_V_offset'],
                      color=set_of_c_stoichiometries[int(c)],lin
```

```
ewidth=1)
            ax15.plot(output_dict[key]['time_axis']/60,output_di
ct[key]['pHlumen'],
                      color=set_of_c_stoichiometries[int(c)],lin
ewidth=1)
            ax18.plot(output_dict[key]['time_axis']/60,output_di
ct[key]['b6f_control'],
                      color=set_of_c_stoichiometries[int(c)],lin
ewidth=1)
            ax21.plot(output_dict[key]['time_axis']/60,output_di
ct[key]['NPQ_array'],
                      color=set_of_c_stoichiometries[int(c)],lin
ewidth=1)

ax1.set_ylabel('pmf (V)',fontsize=7)
ax4.set_ylabel('pmf\n ($\Delta$V)',fontsize=7)
ax7.set_ylabel('$\Delta$$\psi$\n ($\Delta$V)',fontsize=7)
ax10.set_ylabel('$\Delta$pH\n ($\Delta$V)',fontsize=7)
ax13.set_ylabel('Lumen pH',fontsize=7)
ax16.set_ylabel('b$_6$f Rate\n (s$^-$$^1$)',fontsize=7)
ax19.set_ylabel('NPQ',fontsize=7)
ax3b.set_ylabel('Intensity',fontsize=7)
ax6b.set_ylabel('Intensity',fontsize=7)
ax9b.set_ylabel('Intensity',fontsize=7)
ax12b.set_ylabel('Intensity',fontsize=7)
ax15b.set_ylabel('Intensity',fontsize=7)
ax18b.set_ylabel('Intensity',fontsize=7)
ax21b.set_ylabel('Intensity',fontsize=7)

axes = [ax1,ax2,ax3,ax4,ax5,ax6,ax7,ax8,ax9,ax10,ax11,ax12,ax13,
ax14,ax15,ax16,ax17,ax18,
        ax1b,ax2b,ax3b,ax4b,ax5b,ax6b,ax7b,ax8b,ax9b,ax10b,ax11b
,ax12b,ax13b,ax14b,ax15b,
        ax16b,ax17b,ax18b,ax19b,ax20b,ax21b]
for i in range(len(axes)):
    subplot=axes[i]
    subplot.set_xticklabels([])
axes = [ax2,ax3,ax5,ax6,ax8,ax9,ax11,ax12,ax14,ax15,ax17,ax18,ax
20,ax21,
        ax1b,ax2b,ax4b,ax5b,ax7b,ax8b,ax10b,ax11b,ax13b,ax14b,ax
16b,ax17b,ax19b,ax20b]
for i in range(len(axes)):
    subplot=axes[i]
    subplot.set_yticklabels([])
```

```
props = dict(boxstyle='circle', facecolor='white')
labels = ['A1','B1','C1']
axes = [ax1,ax2,ax3]
for i in range(len(axes)):
    subplot=axes[i]
    label=labels[i]
    subplot.text(-2, .45, label, fontsize=6,verticalalignment='t
op', bbox=props)
    subplot.set_ylim(bottom=0, top=0.28)
axes = [ax4,ax5,ax6]
labels = ['A2','B2','C2']
for i in range(len(axes)):
    subplot=axes[i]
    label=labels[i]
    subplot.text(-2, .25, label, fontsize=6,verticalalignment='t
op', bbox=props)
    subplot.set_ylim(bottom=-0.02, top=0.15)
axes = [ax7,ax8,ax9]
labels = ['A3','B3','C3']
for i in range(len(axes)):
    subplot=axes[i]
    label=labels[i]
    subplot.text(-2, .25, label, fontsize=6,verticalalignment='t
op', bbox=props)
    subplot.set_ylim(bottom=-0.05, top=0.15)
axes = [ax10,ax11,ax12]
labels = ['A4','B4','C4']
for i in range(len(axes)):
    subplot=axes[i]
    label=labels[i]
    subplot.text(-2, .08, label, fontsize=6,verticalalignment='t
op', bbox=props)
    subplot.set_ylim(bottom=-0.01, top=0.05)
axes = [ax13,ax14,ax15]
labels = ['A5','B5','C5']
for i in range(len(axes)):
    subplot=axes[i]
    label=labels[i]
    subplot.text(-2, 7.8, label, fontsize=6,verticalalignment='t
op', bbox=props)
    subplot.set_ylim(bottom=5.5, top=7)
axes = [ax16,ax17,ax18]
labels = ['A6','B6','C6']
for i in range(len(axes)):
    subplot=axes[i]
```

```
    label=labels[i]
    subplot.text(-2, 550, label, fontsize=6,verticalalignment='t
op', bbox=props)
    subplot.set_ylim(bottom=0, top=350)
axes = [ax19,ax20,ax21]
labels = ['A7','B7','C7']
for i in range(len(axes)):
    subplot=axes[i]
    label=labels[i]
    subplot.text(-2, 7.5, label, fontsize=6,verticalalignment='t
op', bbox=props)
    subplot.set_ylim(bottom=0, top=5)
    subplot.set_xlabel('Time (min)',fontsize=7)

axes = [ax1,ax4,ax7,ax10,ax13,ax16,ax19]
for i in range(len(axes)):
    subplot=axes[i]
    subplot.tick_params(axis='y',labelsize='small')

axes = [ax1b,ax2b,ax3b,ax4b,ax5b,ax6b,ax7b,
        ax8b,ax9b,ax10b,ax11b,ax12b,ax13b,ax14b,
        ax15b,ax16b,ax17b,ax18b,ax19b,ax20b,ax21b]
for i in range(len(axes)):
    subplot = axes[i]
    subplot.set_ylim(bottom=0, top=425)
    subplot.yaxis.label.set_color('red')
    subplot.spines['right'].set_color('red')
    subplot.tick_params(axis='y', colors='red')
    if i not in [2,5,8,11,14,17,20]:
        subplot.set_yticklabels([])

plt.tight_layout(pad=1, w_pad=0.5, h_pad=0)
plt.show()
print('Simulated responses of the light reactions were performed
as in Davis et al., 2017, with all standard '
      'conditions held constant except for the number of ATP syn
thase c-subunits. Simulations were performed using '
      '1-hour of either static light (A), sinusoidal light (B),
or square wave fluctuating light (C) with equal '
      'total photon flux over the total duration of each light t
reatment. Intervals of light excitation are '
      'indicated by shaded regions. (Panels 1-4) The light—induc
ed pmf of ATP synthases with c—stoichiometries of '
      '17 (purple) or 14 (black) are shown in units of volts, so
that a ΔpH of one is equivalent to 0.06 V. The '
```

```
      'total pmf (panel 2), Δψ (panel 3), and ΔpH (panel 4) are
shown as light-induced changes relative to the pmf '
      'dark values indicated as ΔV from dark values, to emphasiz
e light-induced ATP synthase constraints. (5) '
      'Light-induced changes in lumen pH due to photosynthetic a
ctivity. Light intensities and c-ring composition '
      'as in (1). (6) The relative rate constant for plastoquino
l oxidation at the cytochrome b6f complex and (7) '
      'the extent of nonphotochemical quenching qE component for
each c-ring size due to the light-induced changes '
      'in lumen pH.')
```

Supplemental Figure 7: Altered ATP synthase c17 stoichiometry impacts pmf composition and pH-mediated regulatory processes during photosynthesis under increasingly dynamic light environments.

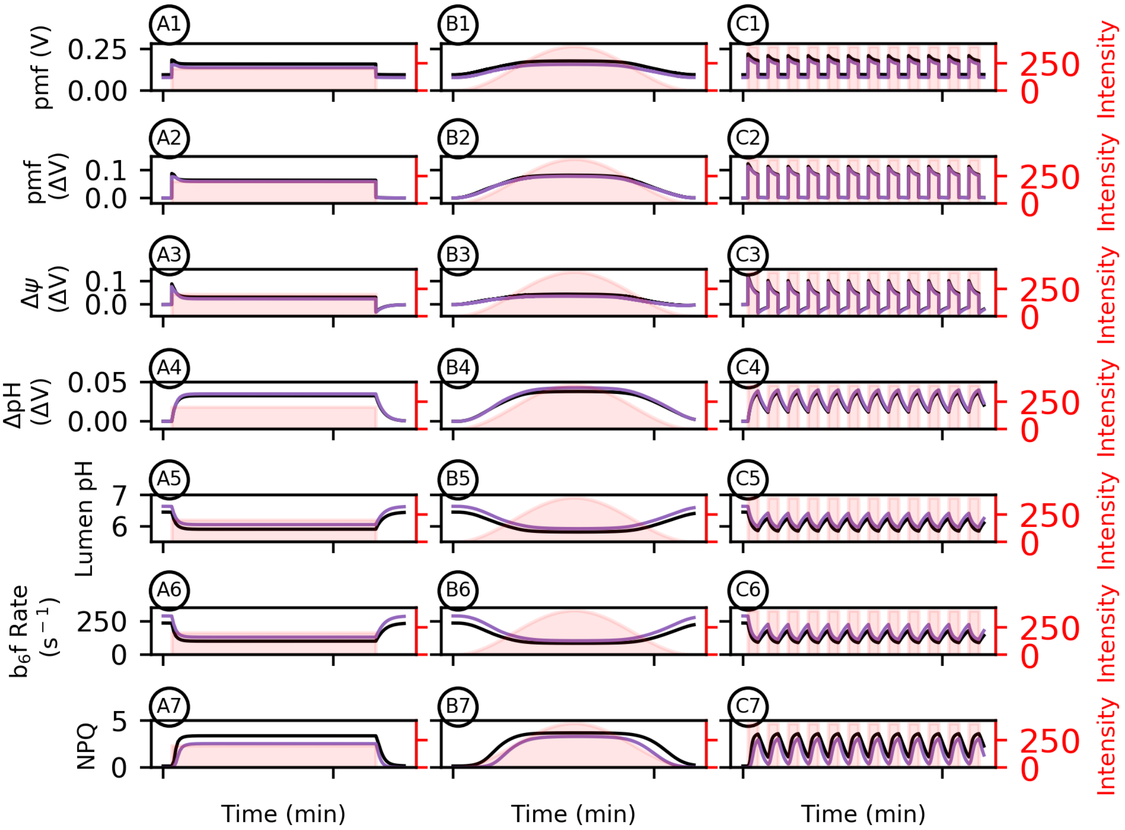

```
Simulated responses of the light reactions were perf
ormed as in Davis et al., 2017, with all standard co
nditions held constant except for the number of ATP
synthase c-subunits. Simulations were performed usin
g 1-hour of either static light (A), sinusoidal ligh
t (B), or square wave fluctuating light (C) with equ
al total photon flux over the total duration of each
light treatment. Intervals of light excitation are i
ndicated by shaded regions. (Panels 1-4) The light–i
nduced pmf of ATP synthases with c–stoichiometries o
f 17 (purple) or 14 (black) are shown in units of vo
lts, so that a ΔpH of one is equivalent to 0.06 V. T
he total pmf (panel 2), Δψ (panel 3), and ΔpH (panel
4) are shown as light–induced changes relative to th
e pmf dark values indicated as ΔV from dark values,
to emphasize light–induced ATP synthase constraints.
(5) Light–induced changes in lumen pH due to photosy
nthetic activity. Light intensities and c–ring compo
sition as in (1). (6) The relative rate constant for
plastoquinol oxidation at the cytochrome b6f complex
and (7) the extent of nonphotochemical quenching qE
component for each c–ring size due to the light–indu
ced changes in lumen pH.
```

In [67]:

```
print('Figure 4: Altered pmf composition due to c-subunit stoich
iometry limits photosynthetic productivity.')

fig = plt.figure('Figure 4', figsize=(5,4), dpi=200)
ax1 = fig.add_subplot(231)
ax1b = ax1.twinx()
ax2 = fig.add_subplot(232)
ax2b = ax2.twinx()
ax3 = fig.add_subplot(233)
ax3b = ax3.twinx()
ax4 = fig.add_subplot(234)
ax4b = ax4.twinx()
ax5 = fig.add_subplot(235)
ax5b = ax5.twinx()
ax6 = fig.add_subplot(236)
ax6b = ax6.twinx()

for key in output_dict:
    [illegible]
```

```
    c=key.split("=")[1]
    if c == "8" or c== "11" or c=='12' or c== "14" or c=="17":
        if "single pulse 201.3umolE subunits" in str(key):
            ax1.plot(output_dict[key]['time_axis']/60,output_dic
t[key]['LEF_cumulative'],
                     color=set_of_c_stoichiometries[int(c)], lab
el="c"+str(c))
            ax1b.fill_between(output_dict[key]['time_axis']/60,
output_dict[key]['light_curve'],0,
                              color='red', alpha=0.02,zorder=2)
            ax4.plot(output_dict[key]['time_axis']/60,output_dic
t[key]['singletO2_array'],
                     color=set_of_c_stoichiometries[int(c)])
            ax4b.fill_between(output_dict[key]['time_axis']/60,
output_dict[key]['light_curve'],0,
                              color='red', alpha=0.02,zorder=2)
        if "single sin wave 402.71umolE subunits" in str(key):
            ax2.plot(output_dict[key]['time_axis']/60,output_dic
t[key]['LEF_cumulative'],
                     color=set_of_c_stoichiometries[int(c)], lab
el=str(c))
            ax2b.fill_between(output_dict[key]['time_axis']/60,
output_dict[key]['light_curve'],0,
                              color='red', alpha=0.02,zorder=2)
            ax5.plot(output_dict[key]['time_axis']/60,output_dic
t[key]['singletO2_array'],
                     color=set_of_c_stoichiometries[int(c)])
            ax5b.fill_between(output_dict[key]['time_axis']/60,
output_dict[key]['light_curve'],0,
                              color='red', alpha=0.02,zorder=2)
        if "square wave 60 min max 400 light subunits" in str(ke
y):
            ax3.plot(output_dict[key]['time_axis']/60,output_dic
t[key]['LEF_cumulative'],
                     color=set_of_c_stoichiometries[int(c)])
            ax3b.fill_between(output_dict[key]['time_axis']/60,
output_dict[key]['light_curve'],0,
                              color='red', alpha=0.02,zorder=2)
            ax6.plot(output_dict[key]['time_axis']/60,output_dic
t[key]['singletO2_array'],
                     color=set_of_c_stoichiometries[int(c)])
            ax6b.fill_between(output_dict[key]['time_axis']/60,
output_dict[key]['light_curve'],0,
                              color='red', alpha=0.02,zorder=2)
```

```
ax1.ticklabel_format(axis='y',style='sci',scilimits=(0,0))
ax2.ticklabel_format(axis='y',style='sci',scilimits=(0,0))
ax3.ticklabel_format(axis='y',style='sci',scilimits=(0,0))
ax4.ticklabel_format(axis='y',style='sci',scilimits=(0,0))
ax5.ticklabel_format(axis='y',style='sci',scilimits=(0,0))
ax6.ticklabel_format(axis='y',style='sci',scilimits=(0,0))

props = dict(boxstyle='circle', facecolor='white')

axes = [ax1,ax2,ax3]
labels = ['A1','B1','C1']
axes_labels = ['LEF (Cumulative)','','']
for i in range(len(axes)):
    subplot = axes[i]
    label = labels[i]
    y_label = axes_labels[i]
    subplot.text(0, 430000, label, fontsize=8,verticalalignment=
'top', bbox=props)
    subplot.set_ylim(bottom=0, top=450000)
    subplot.set_ylabel(y_label)
    if i != 0:
        subplot.set_yticklabels([])

axes = [ax4,ax5,ax6]
labels = ['A2','B2','C2']
axes_labels = [r'$^1$O$_2$ (Cumulative)','','']
for i in range(len(axes)):
    subplot = axes[i]
    label = labels[i]
    y_label = axes_labels[i]
    subplot.text(0, 48000, label, fontsize=8,verticalalignment='
top', bbox=props)
    subplot.set_ylim(bottom=0, top=50000)
    subplot.set_xlabel('Time (min)')
    subplot.set_ylabel(y_label)
    if i != 0:
        subplot.set_yticklabels([])

axes = [ax1b,ax2b,ax3b,ax4b,ax5b,ax6b]
axes_labels = ['','','Intensity','','','Intensity']
for i in range(len(axes)):
    subplot = axes[i]
    y_label = axes_labels[i]
    subplot.set_ylim(bottom=0, top=425)
    [illegible]
```

```
    subplot.yaxis.label.set_color('red')

    subplot.spines['right'].set_color('red')
    subplot.tick_params(axis='y', colors='red')
    subplot.set_ylabel(y_label)
    if i not in [2,5]:
        subplot.set_yticklabels([])

plt.tight_layout(pad=0.4, w_pad=0.5, h_pad=.5)
ax1.legend(bbox_to_anchor=(0.4,0.4),frameon=False, prop={'size':
6})
plt.show()
print('Simulated responses of the light reactions were performed
as in figure 3. Variability in environment was '
      'simulated with 1-hour light profiles of static light (A1,
2), sinusoidal light (B1, 2), or square wave '
      'fluctuating light (C1, 2) to provide the same total illum
ination during the simulation. (1) The total '
      'outputs for linear electron flow (LEF) over the course of
the light simulations and (2) 1O2 were integrated '
      'over the light treatment to give the cumulative totals. S
haded regions indicate the light profiles for each '
      'simulation. ')
```

Figure 4: Altered pmf composition due to c-subunit s
toichiometry limits photosynthetic productivity.

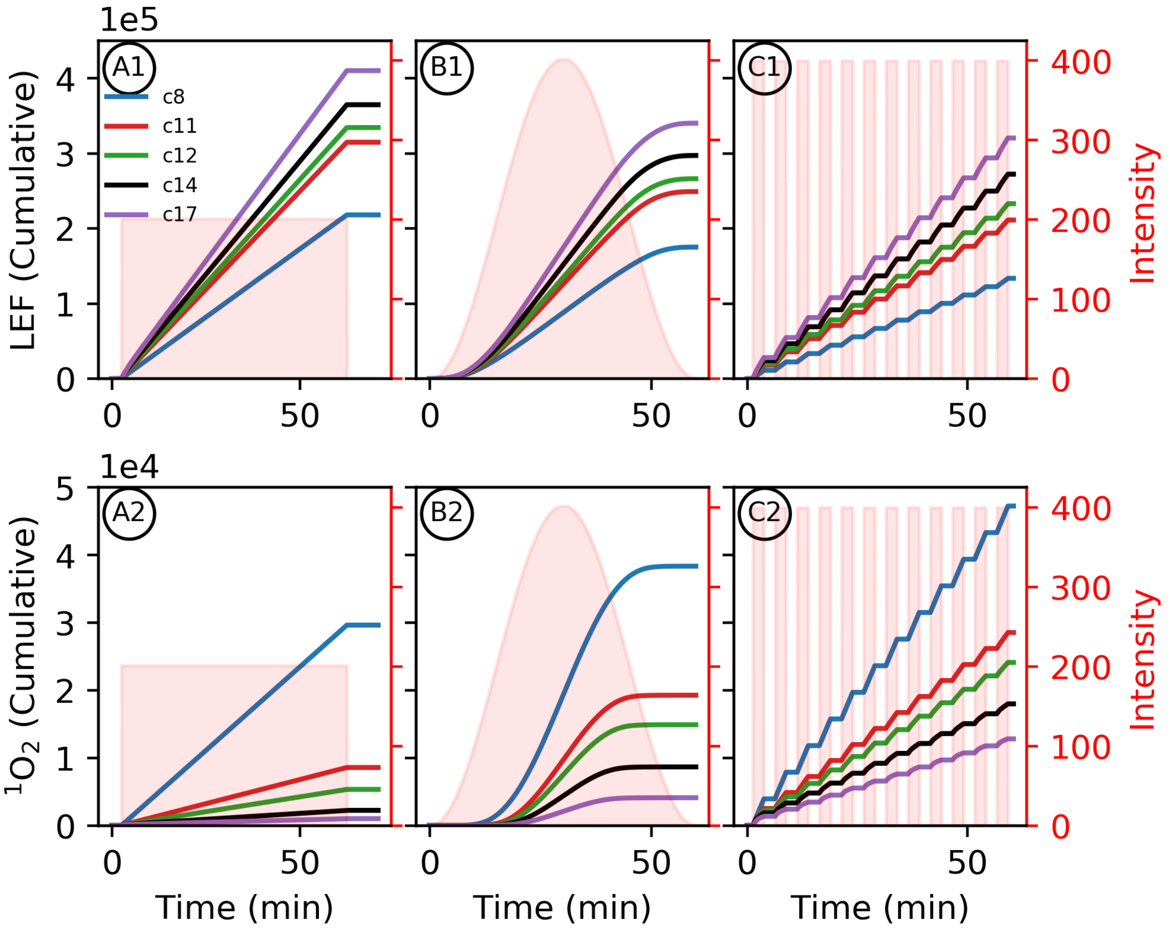

Simulated responses of the light reactions were perf
ormed as in figure 3. Variability in environment was
simulated with 1-hour light profiles of static light
(A1, 2), sinusoidal light (B1, 2), or square wave fl
uctuating light (C1, 2) to provide the same total il
lumination during the simulation. (1) The total outp
uts for linear electron flow (LEF) over the course o
f the light simulations and (2) 1O2 were integrated
over the light treatment to give the cumulative tota
ls. Shaded regions indicate the light profiles for e
ach simulation.

In [68]:

```
print('Supplemental Figure 8: Altered pmf composition due to c-s
ubunit stoichiometry limits photosynthetic '
      'productivity.')

fig = plt.figure('Supplemental Figure 8', figsize=(5,4), dpi=200
)
ax1 = fig.add_subplot(231)
ax1b = ax1.twinx()
ax2 = fig.add_subplot(232)
```

[illegible]

```
ax2b = ax2.twinx()

ax3 = fig.add_subplot(233)
ax3b = ax3.twinx()
ax4 = fig.add_subplot(234)
ax4b = ax4.twinx()
ax5 = fig.add_subplot(235)
ax5b = ax5.twinx()
ax6 = fig.add_subplot(236)
ax6b = ax6.twinx()

for key in output_dict:
    c=key.split('=')[1]
    if "single pulse 201.3umolE subunits" in str(key):
        ax1.plot(output_dict[key]['time_axis']/60,output_dict[ke
y]['LEF_cumulative'],
                 color=set_of_c_stoichiometries[int(c)], label="
c"+str(c))
        ax1b.fill_between(output_dict[key]['time_axis']/60, outp
ut_dict[key]['light_curve'],0,
                          color='red', alpha=0.02,zorder=2)
        ax4.plot(output_dict[key]['time_axis']/60,output_dict[ke
y]['singletO2_array'],
                 color=set_of_c_stoichiometries[int(c)])
        ax4b.fill_between(output_dict[key]['time_axis']/60, outp
ut_dict[key]['light_curve'],0,
                          color='red', alpha=0.02,zorder=2)
    if "single sin wave 402.71umolE subunits" in str(key):
        ax2.plot(output_dict[key]['time_axis']/60,output_dict[ke
y]['LEF_cumulative'],
                 color=set_of_c_stoichiometries[int(c)], label=s
tr(c))
        ax2b.fill_between(output_dict[key]['time_axis']/60, outp
ut_dict[key]['light_curve'],0,
                          color='red', alpha=0.02,zorder=2)
        ax5.plot(output_dict[key]['time_axis']/60,output_dict[ke
y]['singletO2_array'],
                 color=set_of_c_stoichiometries[int(c)])
        ax5b.fill_between(output_dict[key]['time_axis']/60, outp
ut_dict[key]['light_curve'],0,
                          color='red', alpha=0.02,zorder=2)
    if "square wave 60 min max 400 light subunits" in str(key):
        ax3.plot(output_dict[key]['time_axis']/60,output_dict[ke
y]['LEF_cumulative'],
                 color=set_of_c_stoichiometries[int(c)])
```

```
        ax3b.fill_between(output_dict[key]['time_axis']/60, outp
ut_dict[key]['light_curve'],0,
                          color='red', alpha=0.02,zorder=2)
        ax6.plot(output_dict[key]['time_axis']/60,output_dict[ke
y]['singletO2_array'],
                 color=set_of_c_stoichiometries[int(c)])
        ax6b.fill_between(output_dict[key]['time_axis']/60, outp
ut_dict[key]['light_curve'],0,
                          color='red', alpha=0.02,zorder=2)


ax1.ticklabel_format(axis='y',style='sci',scilimits=(0,0))
ax2.ticklabel_format(axis='y',style='sci',scilimits=(0,0))
ax3.ticklabel_format(axis='y',style='sci',scilimits=(0,0))
ax4.ticklabel_format(axis='y',style='sci',scilimits=(0,0))
ax5.ticklabel_format(axis='y',style='sci',scilimits=(0,0))
ax6.ticklabel_format(axis='y',style='sci',scilimits=(0,0))

props = dict(boxstyle='circle', facecolor='white')

axes = [ax1,ax2,ax3]
labels = ['A1','B1','C1']
axes_labels = ['LEF (Cumulative)','','']
for i in range(len(axes)):
    subplot = axes[i]
    label = labels[i]
    y_label = axes_labels[i]
    subplot.text(0, 430000, label, fontsize=8,verticalalignment=
'top', bbox=props)
    subplot.set_ylim(bottom=0, top=450000)
    subplot.set_ylabel(y_label)
    if i != 0:
        subplot.set_yticklabels([])

axes = [ax4,ax5,ax6]
labels = ['A2','B2','C2']
axes_labels = [r'$^1$O$_2$ (Cumulative)','','']
for i in range(len(axes)):
    subplot = axes[i]
    label = labels[i]
    y_label = axes_labels[i]
    subplot.text(0, 48000, label, fontsize=8,verticalalignment='
top', bbox=props)
    subplot.set_ylim(bottom=0, top=50000)
    subplot.set_xlabel('Time (min)')
```

```
    subplot.set_ylabel(y_label)
    if i != 0:
        subplot.set_yticklabels([])

axes = [ax1b,ax2b,ax3b,ax4b,ax5b,ax6b]
axes_labels = ['','','Intensity','','','Intensity']
for i in range(len(axes)):
    subplot = axes[i]
    y_label = axes_labels[i]
    subplot.set_ylim(bottom=0, top=425)
    subplot.yaxis.label.set_color('red')
    subplot.spines['right'].set_color('red')
    subplot.tick_params(axis='y', colors='red')
    subplot.set_ylabel(y_label)
    if i not in [2,5]:
        subplot.set_yticklabels([])

plt.tight_layout(pad=0.4, w_pad=0.5, h_pad=.5)
ax1.legend(bbox_to_anchor=(0.4,0.29),frameon=False, prop={'size'
:6})
plt.show()
print('Simulated responses of the light reactions were performed
as in figure 3. Variability in environment was '
      'simulated with 1-hour light profiles of static light (A1,
2), sinusoidal light (B1, 2), or square wave '
      'fluctuating light (C1, 2) to provide the same total illum
ination during the simulation. (1) The total '
      'outputs for linear electron flow (LEF) over the course of
the light simulations and (2) 1O2 were integrated '
      'over the light treatment to give the cumulative totals. S
haded regions indicate the light profiles for each '
      'simulation. ')
```

Supplemental Figure 8: Altered pmf composition due t
o c-subunit stoichiometry limits photosynthetic prod
uctivity.

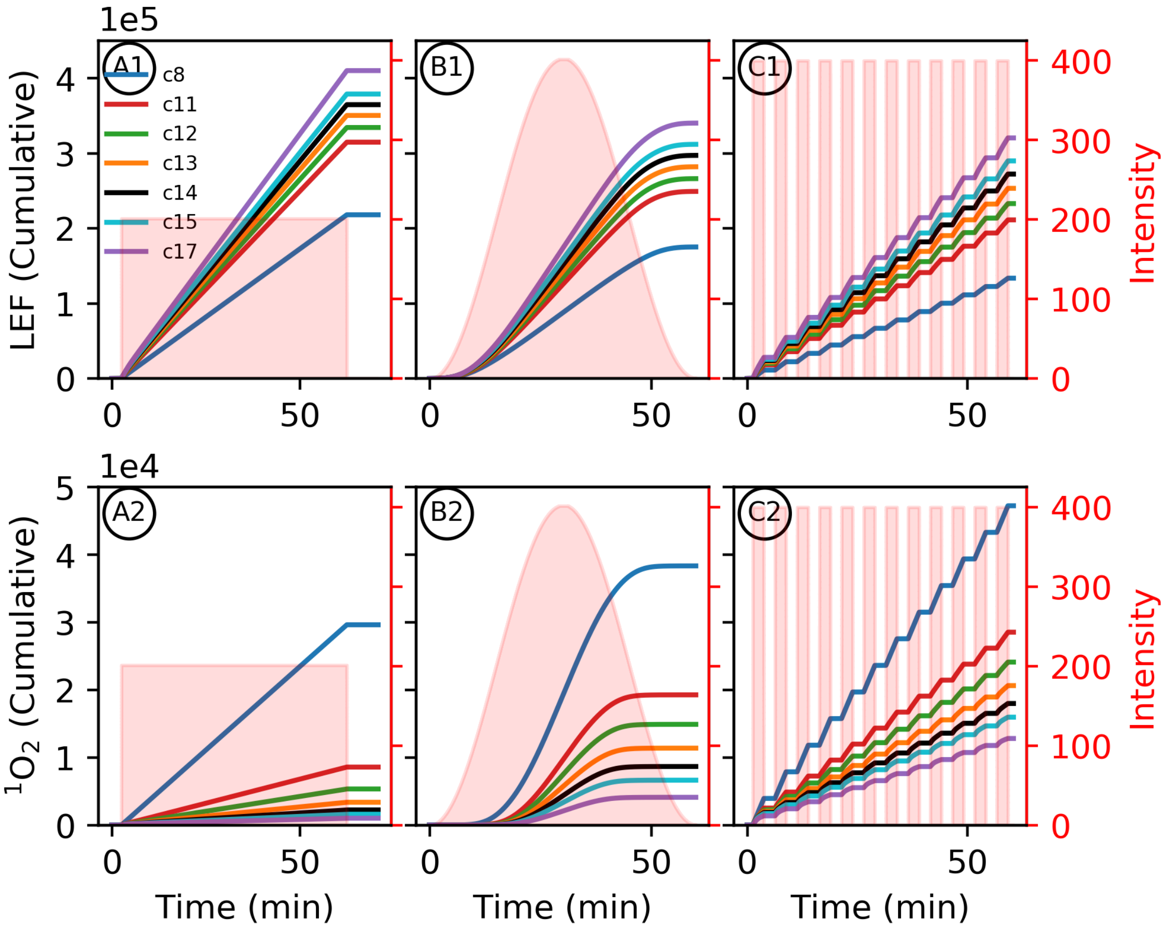

Simulated responses of the light reactions were perf ormed as in figure 3. Variability in environment was simulated with 1-hour light profiles of static light (A1, 2), sinusoidal light (B1, 2), or square wave fl uctuating light (C1, 2) to provide the same total il lumination during the simulation. (1) The total outp uts for linear electron flow (LEF) over the course o f the light simulations and (2) 1O2 were integrated over the light treatment to give the cumulative tota ls. Shaded regions indicate the light profiles for e ach simulation.
